# SRSF3 and SRSF7 modulate 3′UTR length through suppression or activation of proximal polyadenylation sites and regulation of CFIm levels

**DOI:** 10.1186/s13059-021-02298-y

**Published:** 2021-03-11

**Authors:** Oliver Daniel Schwich, Nicole Blümel, Mario Keller, Marius Wegener, Samarth Thonta Setty, Melinda Elaine Brunstein, Ina Poser, Igor Ruiz De Los Mozos, Beatrix Suess, Christian Münch, François McNicoll, Kathi Zarnack, Michaela Müller-McNicoll

**Affiliations:** 1 Institute for Molecular Bio Science, Goethe University Frankfurt, Max-von-Laue-Str. 13, 60438 Frankfurt, Germany; 2 Buchmann Institute for Molecular Life Sciences, Goethe University Frankfurt, Max-von-Laue-Str. 15, 60438 Frankfurt, Germany; 3 Faculty of Biological Sciences, Goethe University Frankfurt, 60438 Frankfurt am Main, Germany; 4 Institute of Biochemistry II, Medical School, Goethe University Frankfurt, Sandhofstr. 2-4, 60528 Frankfurt am Main, Germany; 5 Max Planck Institute of Molecular Cell Biology and Genetics, Pfotenhauerstr. 108, 01307 Dresden, Germany; 6 The Francis Crick Institute, 1 Midland Road, London, NW1 1AT UK; 7 Department of Biology, Technical University Darmstadt, Schnittspahnstr. 10, 64287 Darmstadt, Germany

**Keywords:** SRSF3, SRSF7, APA, FIP1, CFIm, pPAS, dPAS, iCLIP, MACE-seq, 3′UTR length

## Abstract

**Background:**

Alternative polyadenylation (APA) refers to the regulated selection of polyadenylation sites (PASs) in transcripts, which determines the length of their 3′ untranslated regions (3′UTRs). We have recently shown that SRSF3 and SRSF7, two closely related SR proteins, connect APA with mRNA export. The mechanism underlying APA regulation by SRSF3 and SRSF7 remained unknown.

**Results:**

Here we combine iCLIP and 3′-end sequencing and find that SRSF3 and SRSF7 bind upstream of proximal PASs (pPASs), but they exert opposite effects on 3′UTR length. SRSF7 enhances pPAS usage in a concentration-dependent but splicing-independent manner by recruiting the cleavage factor FIP1, generating short 3′UTRs. Protein domains unique to SRSF7, which are absent from SRSF3, contribute to FIP1 recruitment. In contrast, SRSF3 promotes distal PAS (dPAS) usage and hence long 3′UTRs directly by counteracting SRSF7, but also indirectly by maintaining high levels of cleavage factor Im (CFIm) via alternative splicing. Upon SRSF3 depletion, CFIm levels decrease and 3′UTRs are shortened. The indirect SRSF3 targets are particularly sensitive to low CFIm levels, because here CFIm serves a dual function; it enhances dPAS and inhibits pPAS usage by binding immediately downstream and assembling unproductive cleavage complexes, which together promotes long 3′UTRs.

**Conclusions:**

We demonstrate that SRSF3 and SRSF7 are direct modulators of pPAS usage and show how small differences in the domain architecture of SR proteins can confer opposite effects on pPAS regulation.

**Supplementary Information:**

The online version contains supplementary material available at 10.1186/s13059-021-02298-y.

## Background

Cleavage and polyadenylation (CPA) is an important step of gene expression that results in the endonucleolytic cleavage of pre-mRNAs and the subsequent addition of a non-templated poly(A) tail of ~ 150 nt by the poly(A) polymerase (PAP) [1]. CPA is mediated by the recruitment of *trans*-acting factors to *cis*-acting sequence elements within pre-mRNAs that are highly conserved and well defined [2]. At each polyadenylation site (PAS), the CPA machinery assembles from four subcomplexes—cleavage and polyadenylation specificity factor (CPSF), cleavage stimulatory factor (CstF), cleavage factor Im (CFIm), and IIm (CFIIm) [3]. The CPSF complex is composed of six subunits (CPSF160 [CPSF1], CPSF100 [CPSF2], CPSF73 [CPSF3], CPSF30 [CPSF4], WDR33, and FIP1 [FIP1L1]) and CstF of three subunits (CstF50 [CSTF1], CstF64 [CSTF2], and CstF77 [CSTF3]). CPSF and CstF are sufficient to catalyze CPA in vitro [4, 5]. CPSF30 and WDR33 recognize the hexameric central sequence element (CSE), usually AAUAAA [6, 7], CstF64 binds to GU-rich downstream sequence elements (DSEs) [8], and CPSF73 acts as the endonuclease [9]. FIP1 binds to U-rich sequence elements (USEs) upstream of PASs and recruits PAP. FIP1 binding switches PAP from a slow into a fast, processive state [10]. CFIm is a tetramer consisting of two small 25 kDa subunits (CPSF5 [NUDT21]) and two large subunits of 68 kDa or 59 kDa (CPSF6 or CPSF7) that bind CPSF5 separately or together [11–13]. The large subunits play a structural role, bringing together the two CPSF5 subunits that each bind to a UGUA sequence upstream of most PASs, thereby providing sequence specificity to the complex [14, 15]. However, while CPSF6 enhances PAS usage through FIP1 recruitment, CPSF7 does not stimulate CPA [16, 17]. CFIIm contains two subunits, PCF11 and CLP1, and has recently been shown to play a role in transcription termination [18].

Around 70% of all mammalian genes contain more than one PAS and express transcript isoforms that differ in the PASs that are used through a process termed alternative polyadenylation (APA) [19]. APA affects either the coding potential of transcripts (CR-APA), when the PAS is located upstream of the stop codon, or the length of the 3′ untranslated region (3′UTR-APA) and hence the potential of mRNAs to be regulated [20]. 3′UTR sequence elements regulate the stability, nuclear export, localization, and translation efficiency of transcripts as well as the localization of their protein products [21]. Dysfunctional APA has been implicated in various human diseases including cancer [22, 23].

3′UTR-APA is regulated in *cis* through the intrinsic strength of alternative PASs, which is determined by the sequence composition of their CSEs, DSEs, and USEs, the number of UGUA motifs and their distance to the cleavage site, as well as the presence of binding sites for additional APA regulators [24–26]. APA is also regulated in *trans* through the levels of CPA complex subunits. For example, FIP1 promotes the usage of proximal PASs (pPASs) and the generation of transcripts with short 3′UTRs, which is important during stem-cell self-renewal [27]. In contrast, CFIm binds preferentially to distal PASs (dPASs) and enhances their usage, promoting the expression of isoforms with long 3′UTRs [28]. Accordingly, depletion of the CFIm subunits CPSF5 and CPSF6 causes global 3′UTR shortening [29, 30].

Beside core CPA factors, other RNA-binding proteins (RBPs) have been identified as global 3′UTR-APA regulators. Interestingly, many of them are splicing factors, such as CELF2, TARDBP, FUS, HNRNPC, and NOVA2 [19, 24, 31]. We have previously reported that two splicing factors, SRSF3 and SRSF7, connect APA to selective mRNA export via recruitment of the mRNA export factor NXF1 [32]. The APA function of SRSF3 was subsequently shown to regulate cellular senescence [33]. While SRSF3 is already known to regulate the splicing of terminal exons and thereby CR-APA [34], how SRSF3 and SRSF7 regulate 3′UTR-APA and whether this function depends on splicing remain to be determined.

SRSF3 and SRSF7 are members of the SR protein family, which comprises 12 canonical members [35]. Both proteins are very closely related and share a similar domain architecture with one RNA recognition motif (RRM, 80% identical), a short linker region for NXF1 interaction, and a region enriched in arginine-serine di-peptides called RS domain [36–38]. Unlike SRSF3, SRSF7 contains an additional CCHC-type zinc (Zn) knuckle domain that together with the RRM determines its distinct RNA-binding specificity [39–41]. In addition, the RS domain of SRSF7 is 66 amino acids longer and harbors a distinct hydrophobic stretch [41]. RS domains mediate protein-protein interactions and, through phosphorylation and dephosphorylation of their serine residues, the splicing activity, nuclear import, subnuclear localization, and mRNA export activity of SR proteins are regulated [42].

Here, we investigated the mechanisms underlying 3′UTR-APA regulation by SRSF3 and SRSF7. We found that both proteins bind upstream of pPASs but exert opposite effects on the length of 3′UTRs. SRSF7 activates pPAS usage directly in a splicing-independent manner via recruitment of FIP1, thus promoting short 3′UTRs. In contrast, SRSF3 promotes long 3′UTRs by inhibiting pPAS usage either directly or indirectly by controlling the levels of active CFIm through alternative splicing. We found that SRSF3-regulated 3′UTRs are particularly sensitive to low CFIm levels, because CFIm inhibits pPAS usage and enhances dPAS usage in these transcripts. Given that SRSF3 and SRSF7 both recruit NXF1 for mRNA export [32], their binding and action at pPASs provide the means to sort mRNAs with different 3′UTR lengths for later steps in cytoplasmic gene expression, such as RNA localization and translation.

## Results

### SRSF3 and SRSF7 exert opposite effects on 3′UTR length

To study the mechanisms of 3′UTR-APA regulation by SRSF3 and SRSF7, we first acquired a comprehensive list of their APA targets in murine pluripotent P19 cells. We depleted each protein individually using esiRNAs (endoribonuclease-prepared siRNAs; Additional file 1: Fig. S1A) and sequenced poly(A) + RNA (50–60 million reads per sample). We used DaPars to identify pPASs and to quantify their usage based on changes in read coverage [43].

**Fig. 1:**
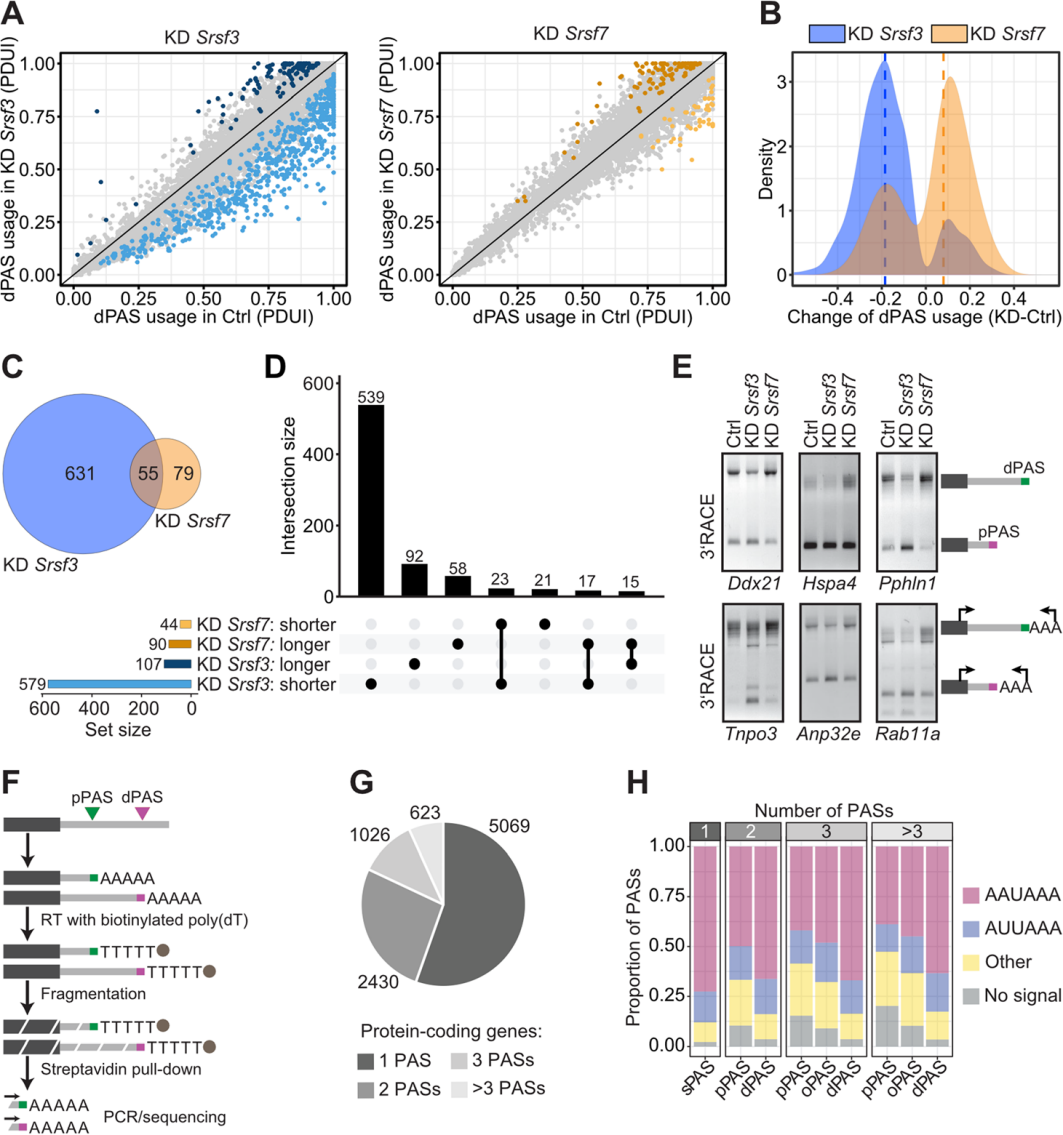
SRSF3 and SRSF7 exert opposite effects on 3′UTR length. **a** Scatter plots of *p*ercentage of *d*istal poly(A) site *u*sage *i*ndex (PDUI) in *Srsf3* (left) and *Srsf7* (right) KD and control (Ctrl) P19 cells. Significant changes highlighted in blue and orange, respectively (adjusted *P* value ≤ 0.1, |ΔPDUI| ≥ 0.05). **b** Density plot of differences in ΔPDUI for significant changes shown in **a**. Dashed lines indicate median. **c**, **d** Venn diagram (**c**) and UpSet plot (**d**) of genes with significant changes in 3′UTR-APA after *Srsf3* or *Srsf7* KD. Numbers of target genes and direction regulated by either SR protein are given below. **e** Semiquantitative 3′RACE-PCR of selected transcripts affected by *Srsf3* or *Srsf7* KD. Proximal (pPAS; pink) or distal (dPAS; green) PAS usage as well as the positions of primers are indicated next to the gels. **f** Scheme of the MACE-seq library preparation. RT, reverse transcription. **g** Pie chart of protein-coding genes harboring one, two, three, or more PASs identified by MACE-seq. **h** Stacked bar charts show the proportion of PASs associated with a given CSE in gene categories as in **c**. Analyzed PASs included single (sPASs), pPASs, dPASs, and other PASs (oPASs)

Knockdown (KD) of *Srsf3* significantly affected 3′UTR-APA of 686 genes (difference in *p*ercentage of *d*istal poly(A) site *u*sage *i*ndex [|ΔPDUI|] ≥ 0.05, false discovery rate [FDR] ≤ 0.1). Almost all targets showed an increased usage of the pPAS, resulting in 3′UTR shortening (579 genes, 84%; Fig. 1a, b, Additional file 2: Table S1). In contrast, *Srsf7* KD enhanced dPAS usage and 3′UTR lengthening (90 out of 134 genes, 67%). Despite the smaller number of genes affected by *Srsf7* KD, there was a considerable overlap (55 genes; Fig. 1c, *P* value = 2.5e−41, Fisher’s exact test), with 17 genes being regulated antagonistically by SRSF3 and SRSF7 (Fig. 1d). Analysis of the data with MISO TandemUTR annotations [44] confirmed the trend of 3′UTR shortening and lengthening upon *Srsf3* and *Srsf7* KD, respectively (Additional file 1: Fig. S1B, Additional file 3: Table S2). SRSF3 target genes with shortened 3′UTRs were enriched for functions related to cell cycle progression and chromosome segregation (Additional file 1: Fig. S1C), indicating that SRSF3 regulates cell proliferation and growth via 3′UTR length control, as suggested in a previous study [33]. Changes in 3′UTR length were validated for six genes that were antagonistically regulated by SRSF3 and SRSF7 using semi-quantitative 3′RACE-PCR (rapid amplification of cDNA ends; Fig. 1e). As observed in the DaPars/MISO analyses, *Srsf3* KD promoted the use of their pPASs and *Srsf7* KD the use of their dPASs. We concluded that SRSF3 and SRSF7 have opposite effects on 3′UTR-APA in hundreds of cellular transcripts and act antagonistically on a subset of targets.

In order to precisely map the PASs used in murine pluripotent P19 cells, we performed MACE-seq (massive amplification of cDNA ends), a high-throughput 3′end sequencing method that enables the identification of PASs at single-nucleotide resolution [45, 46] (Fig. 1f). MACE-seq was performed with RNA from control, *Srsf3* and *Srsf7* KD cells to capture all PASs that are used in these conditions (Additional file 1: Fig. S1D). For PAS mapping, we pooled all MACE-seq samples and used a customized analysis pipeline to remove priming artifacts (Additional file 1: Fig. S1E, see the “Methods” section). We identified a total of 15,866 high-confidence PASs mapping to 9148 genes. Most PASs were found in protein-coding genes (15,805), within their 3′UTRs (13,706) (Additional file 1: Fig. S1F, S1G).

The majority of protein-coding genes (5069 genes, 55%) used only a single PAS (sPAS) in murine P19 cells. However, 4079 protein-coding genes (45%) showed clear evidence for APA, with 2430, 1026, and 623 genes harboring two, three, or more PASs, respectively (Fig. 1g). Depending on their relative position, we assigned the first, last, and intermediate PASs within each 3′UTR as proximal (pPAS), distal (dPAS), and other (oPAS), respectively. The PAS positions agreed well with GENCODE annotations (version M18), as most PASs (61%) mapped within 25 nt of an annotated transcript 3′end (Additional file 1: Fig. S1H, S1I). In addition, we detected 6207 PASs that were further downstream and likely belonged to non-annotated alternative isoforms (Additional file 1: Fig. S1H, S1I).

Screening for the 18 known CSE motifs [24] upstream of all PASs used in P19 cells, we found that 93% of all PASs contained a CSE variant (Additional file 1: Fig. S1J). The canonical AAUAAA hexamer was most common (59.5%), followed by the variants AUUAAA (19.9%), UAUAAA (7.4%), AAGAAA (6.3%), and AAUAUA (5.4%). In genes with multiple PASs, AAUAAA was predominantly found at dPASs, whereas alternative CSEs were predominant at pPASs and oPASs (Fig. 1h). This is in line with previous studies suggesting that pPASs are generally weaker and subject to regulation, while dPASs are stronger and used by default [47]. Similarly, almost 90% of sPASs harbored one of the two most frequent CSEs, indicating that sPASs are strong and well-defined in motif composition. Our data represent the first atlas of PAS positions and CSE composition in mouse pluripotent P19 cells and suggest that almost half of all expressed genes generate more than one 3′UTR isoform, indicating extensive gene expression regulation at the level of 3′UTR-APA.

### SRSF7 binds preferentially at pPASs and modulates their usage in a splicing-independent manner

**Fig. 2:**
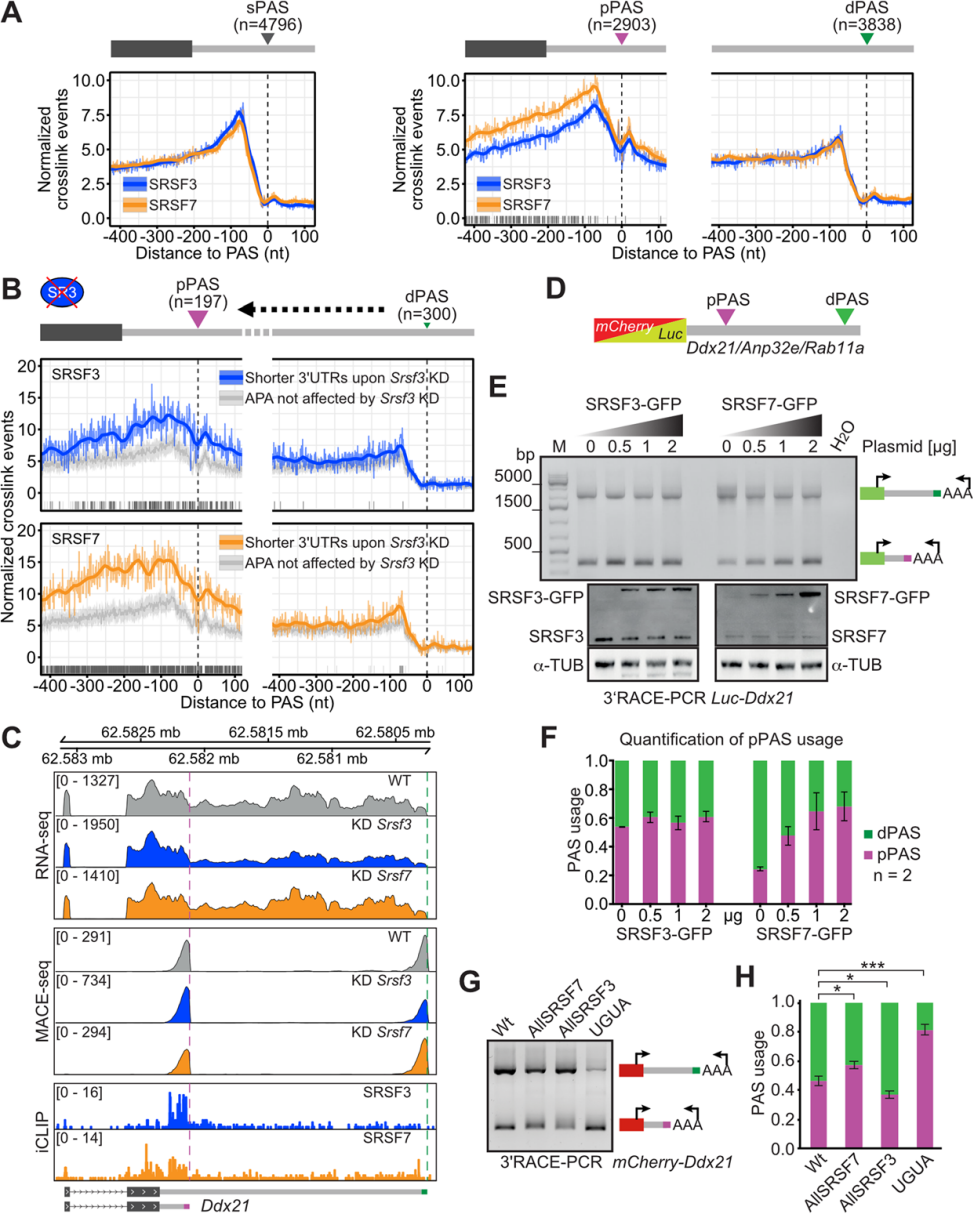
SRSF7 binds preferentially at pPASs and modulates their usage in a splicing-independent manner. **a** Metaprofiles of normalized iCLIP crosslink events of SRSF3 and SRSF7 around sPASs (left), pPASs and dPASs (right). Raw signal and loess smoothing are shown. Positions with a significant signal difference between SRSF3 and SRSF7 are marked below (false discovery rate [FDR] ≤ 0.01, two-proportions *z*-test). **b** Metaprofiles of normalized iCLIP crosslink events of SRSF3 and SRSF7 around pPASs and dPASs of transcripts with shorter 3′UTRs upon *Srsf3* KD (SRSF3 targets) compared to randomly sampled PASs that are not affected by *Srsf3* KD. Raw signal and loess smoothing are shown. Significant differences in SRSF3 and SRSF7 binding are computed by a *z*-score approach. Positions with FDR ≤ 0.01 are indicated below. **c** Genome browser view of the *Ddx21* 3′UTR: Top: RNA-seq coverage in control conditions and after *Srsf3* and *Srsf7* KD. Middle: MACE-seq coverage at the pPAS and dPAS. Bottom: iCLIP crosslink events of SRSF3 and SRSF7. Merged replicates are shown for all datasets. **d** Scheme of reporter genes. *mCherry* and *Luciferase* (*Luc*) were fused with endogenous 3′UTRs of *Ddx21*, *Anp32e*, and *Rab11a*. **e** Semiquantitative 3′RACE-PCR (top) of *Luc-Ddx21* reporter upon SRSF3-GFP or SRSF7-GFP overexpression, validated by Western blot (bottom). Antibody against α-tubulin (α-TUB) was used as loading control. **f** Quantification of 3′RACE-PCRs (*n* = 2) of *Luc*-*Ddx21* reporter transcripts upon SRSF3-GFP and SRSF7-GFP overexpression. Data are represented as mean ± standard deviation of mean. **g** Semiquantitative 3′RACE-PCR of transiently expressed mutated *mCherry-Ddx21* reporters. **h** Quantification of 3′RACE-PCRs (*n* = 3) of mutated *mCherry*-*Ddx21* reporter transcripts. Data are represented as mean ± standard deviation of mean. Student’s *t* test, *P value < 0.05, ***P value < 0.0005

To investigate whether SRSF3 and SRSF7 directly affect 3′UTR-APA, we compared their binding at our mapped sPASs, pPASs, and dPASs using our previously published iCLIP (individual-nucleotide resolution UV crosslinking and immunoprecipitation) data sets obtained from P19 cells [32]. In line with a direct involvement in CPA, both proteins showed a pronounced binding peak ~ 75 nt upstream of sPASs, pPASs, and dPASs in a metaprofile analysis (Fig. 2a). An additional, minor binding peak was present ~ 20 nt downstream of all PASs and especially conspicuous at pPASs. In genes that undergo 3′UTR-APA, both proteins showed a strong preference for pPASs, where binding of SRSF7 exceeded that of SRSF3. In contrast, binding of both proteins was similar at sPASs and dPASs. To confirm that this is also true for genes whose APA is regulated by SRSF3 and SRSF7, we integrated the PAS coordinates from MACE-seq with 3′UTR changes from the DaPars analysis and precisely mapped PASs for 361 out of 686 SRSF3 targets (Additional file 1: Fig. S2A, S2B). Indeed, binding of both SRSF3 and SRSF7 was much more frequent at SRSF3-regulated pPASs, compared to pPASs that are not affected by *Srsf3* KD, but there was no binding difference at dPASs (Fig. 2b). This suggests that the pPAS is the primary point of APA regulation by SRSF3 and SRSF7 and that both proteins might compete here for binding. To further strengthen this idea, we tested whether the binding motifs of SRSF3 (CNYC, C, cytosine; N, any nucleotide; Y, pyrimidine) and SRSF7 (GAY, G, guanine; A, adenine) are enriched around sPASs, pPASs, and dPASs [32, 48]. We generally observed an enrichment of both motifs upstream of sPASs and pPASs (Additional file 1: Fig. S2C, S2F), suggesting that both proteins can actively bind to pPASs rather than being recruited by other proteins. Of note, the SRSF7 binding motif GAY was particularly enriched at SRSF3-regulated pPASs compared to unregulated pPASs (Additional file 1: Fig. S2G), reflecting its enhanced binding at these sites in our iCLIP data (Fig. 2b). A similar pattern could not be observed for the SRSF3 binding motif CNYC motif (Additional file 1: Fig. S2D). In contrast, none of the motifs were enriched at dPASs (Additional file 1: Fig. S2E, S2H), underlining that pPASs are the regulatory hotspots of APA regulation by SRSF3 and SRSF7.

To test whether APA regulation by SRSF3 and SRSF7 is splicing-dependent, we selected three validated target genes—*Ddx21*, *Anp32e*, and *Rab11a* (Fig. 1e)—for a reporter gene study. All three genes showed increased pPAS usage upon *Srsf3* KD, increased dPAS usage upon *Srsf7* KD, and binding of both proteins upstream of the pPAS (Fig. 2c, Additional file 1: Fig. S2I, S2J). We fused the complete 3′UTRs (including 150 nt downstream of the dPASs) to two distinct reporter genes, encoding Firefly Luciferase (*Luc*) and mCherry (Fig. 2d). After transfection into P19 cells, pPAS usage was determined by 3′RACE-PCR. All reporter constructs produced alternative 3′UTR isoforms of similar length as the endogenous genes (Additional file 1: Fig. S3A, S3B). Importantly, *Srsf3* KD caused similar changes in pPAS usage in reporter transcripts and their endogenous counterparts (Additional file 1: Fig. S3C, S3D). The effect of the *Srsf7* KD could be recapitulated in some but not all cases. Since the reporter constructs did not contain any introns, the observed 3′UTR changes indicated that 3′UTR-APA regulation by SRSF3 and SRSF7 is independent of splicing of the respective transcripts.

To evaluate the dose-response relationship of this regulation, we co-transfected the *Luc-Ddx21* reporter with increasing amounts of SRSF3-GFP and SRSF7-GFP expression plasmids (Fig. 2e, f). Overexpression (OE) of SRSF7-GFP had the opposite effect of *Srsf7* KD and resulted in a concentration-dependent increase of the shorter *Luc-Ddx21* 3′UTR isoform (Fig. 2e, f). OE of SRSF3-GFP did not further increase the levels of the longer *Luc-Ddx21* isoform, but here the levels of SRSF3 might not have been sufficient.

We next tested whether the extent of binding upstream of pPASs by either SRSF3 or SRSF7 directly affects pPAS usage. To do this, we converted all SRSF3 binding motifs into SRSF7 binding motifs (allSRSF7) and vice versa (allSRSF3) within a region of 110 nt immediately upstream of the pPAS in the *mCherry-* and *LUC-Ddx21* reporter genes (Additional file 1: Fig. S3E). Of note, this region does not contain UGUA motifs. Consistent with the results presented above, shifting the binding potential towards SRSF7 (allSRSF7) increased pPAS usage and shifting it to SRSF3 (allSRSF3) decreased pPAS usage (Fig. 2g, h, Additional file 1: Fig. S3F). Importantly, single point mutations in the alternative CSE of the pPAS that strengthened it (AGUAAA to AAUAAA) completely abrogated dPAS usage, whereas its weakening (AGUAAA to AGUAAG) [49] abrogated pPAS usage (Fig. 2h, Additional file 1: Fig. S3F). Moreover, inserting one UGUA motif in the absence of any SRSF3 and SRSF7 binding sites also strongly increased pPAS usage (Fig. 2g, h). This suggests that an intermediate CSE and the absence of UGUA motifs at the pPAS allow SRSF7 and SRSF3 to modulate its usage in opposite directions. Whereas SRSF7 enhances pPAS usage, SRSF3 inhibits pPAS usage in a binding- and concentration-dependent manner.

### SRSF7 interacts with FIP1 independently of RNA via its hypo-phosphorylated RS domain

The binding of SRSF3 and SRSF7 upstream of pPASs suggests that they might interact with the CPA machinery. In line with this possibility, quantitative mass spectrometry (MS) using TMT-labeling of immunopurified RNPs containing SRSF3-GFP identified several CPA factors, including components of the CPSF complex (FIP1, CPSF2, CPSF3, and WDR33) and CPSF5 from the CFIm complex (Additional file 1: Fig. S4A, Additional file 4: Table S3). To confirm these interactions, we performed semi-quantitative co-immunoprecipitations (Co-IPs) with and without RNase treatment. We focused hereby on CPSF5 and CPSF6 from the CFIm complex [28] and on FIP1 as representative of the CPSF complex, since CPSF6 and FIP1 both contain an RS-like domain (Additional file 1: Fig. S4B, S4C). We generated P19 cell lines expressing GFP-tagged CPSF5, CPSF6, and FIP1 from genomic loci (Additional file 1: Fig. S4D-G). Since CPSF5-GFP performed much better than CPSF6-GFP in initial pulldown tests (data not shown), we used CPSF5-GFP for the IP experiments.

**Fig. 3:**
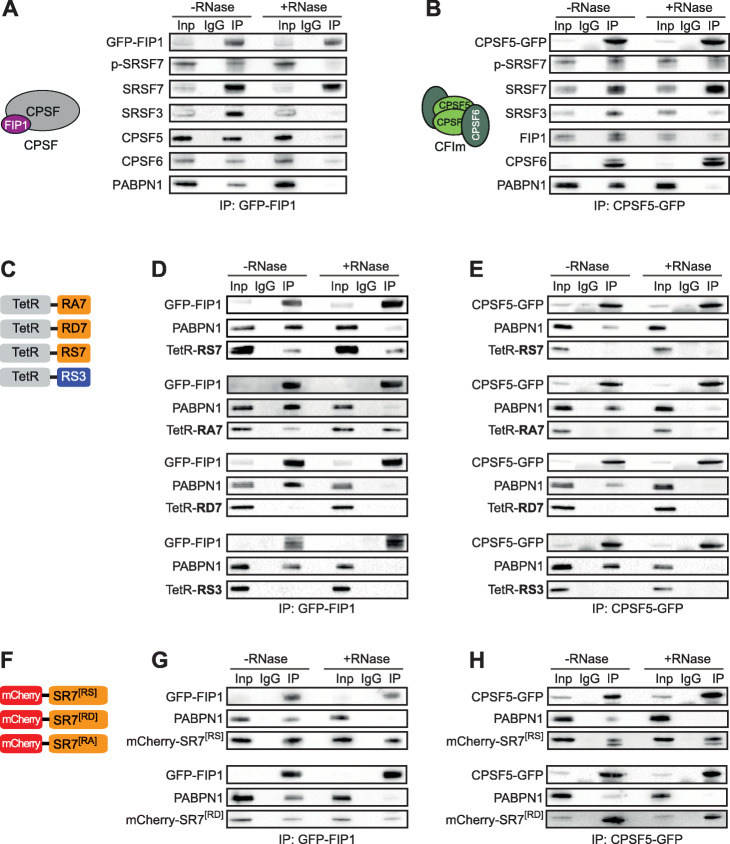
SRSF7 interacts RNA-independently with FIP1 via its hypo-phosphorylated RS domain. **a**, **b** Co-immunoprecipitations (Co-IPs) using GFP-tagged cleavage factors FIP1 (**a**) and CPSF5 (**b**): proteins were pulled-down by α-GFP antibodies and probed for interaction partners using specific antibodies. Lysates were treated with (+RNase) or without (−RNase) RNase A prior to IPs. PABPN1 served as a control for RNA degradation. Inp, input; IP, immunoprecipitation; IgG, unspecific antibody control. **c** Scheme of tetracyclin repressor (TetR) protein fused to RS domains of SRSF3 (RS3), SRSF7 (RS7), or phosphomimetic (RD7) and non-phosphorylatable (RA7) versions of SRSF7. **d**, **e** Co-IPs for TetR using GFP-FIP1 (**d**) and CPSF5-GFP (**e**) cell lines: Proteins were pulled-down by α-GFP antibodies and probed for interaction with TetR-fusion proteins using α-TetR antibodies. RNase A treatment and loading control as in **a**. **f** Scheme of mCherry fused to the complete coding sequence of WT SRSF7[RS] or its variants SRSF7[RD]/[RA]. **g**, **h** Co-IPs using GFP-FIP1 (**g**) and CPSF5-GFP (**h**) cell lines: Proteins were pulled-down by α-GFP antibodies and probed for interaction with mCherry fusion proteins using an α-mCherry antibody. RNase A treatment and loading control as in (**a**)

CPSF5-GFP efficiently co-immunoprecipitated its CFIm complex partner CPFS6, while the association of both proteins with the CPSF complex protein FIP1 was RNase-sensitive (Fig. 3a, b). Both CPA factors co-immunoprecipitated SRSF3, but surprisingly, the signal disappeared after RNase treatment, suggesting an indirect association via co-bound (pre-)mRNAs. In contrast, SRSF7 was efficiently co-immunoprecipitated in the presence of RNase, supporting a direct interaction of SRSF7 with both CPSF5 and FIP1. The results were confirmed by reverse Co-IPs using GFP-tagged SRSF3 and SRSF7 [32] as baits (Additional file 1: Fig. S4H, S4I). These data suggest that binding of SRSF7 upstream of pPASs might enhance their usage through the recruitment of CPA factors, whereas competitive binding to the same sites by SRSF3 might impair their recruitment.

It was recently shown that the RS domain of CPSF6 is required for FIP1 recruitment and dPAS activation [17]. RS domains are also the main protein-protein interaction platforms of SR proteins [42], and their dimerization is regulated through phosphorylation/dephosphorylation of their serine residues [50]. To test whether SRSF7 recruits CFIm and FIP1 via its RS domain, we generated C-terminal fusions of the tetracycline repressor protein (TetR) with the RS domain of either SRSF3 (RS3) or SRSF7 (RS7). We included variants of SRSF7’s RS domain in which all serine residues were exchanged for alanine (RA7) or aspartate (RD7) residues, thereby mimicking hypo- and hyper-phosphorylated RS domains, respectively (Fig. 3c). TetR constructs were transfected into GFP-FIP1 and CPSF5-GFP cell lines, and expression and phosphorylation of all protein chimeras were verified by Western blot following phosphatase treatment (Additional file 1: Fig. S5A, S5B).

Co-IPs confirmed that the RS domain of SRSF7 (RS7) was sufficient to mediate interaction of TetR with GFP-FIP1 (Fig. 3d). Strikingly, this interaction was maintained with the RA7 variant but lost with the RD7 variant, suggesting that FIP1 interacts preferentially with the hypo-phosphorylated RS domain of SRSF7. In line with this, SRSF7 that co-immunoprecipitated with GFP-FIP1 appeared hypo-phosphorylated as it did not react with mAb104, an antibody that recognizes exclusively hyper-phosphorylated domains of SR proteins (see p-SRSF7 in Fig. 3a) [51]. In contrast, the isolated RS domain of SRSF7 was not sufficient to promote interaction of TetR with CPSF5, regardless of its phosphorylation state (Fig. 3e). This suggested that interaction of SRSF7 with CPSF5 requires its RNA-binding domain. In line with the results described above, the isolated RS domain of SRSF3 (RS3) did not promote interaction of TetR with GFP-FIP1 or CPSF5-GFP in P19 cells (Fig. 3d, e).

To assess whether binding of SRSF7 to CPSF5 is also phosphorylation-dependent, we made full-length SRSF7 constructs (mCherry-SRSF7^[RS]^) and replaced all serine residues within the RS domain with either alanine (mCherry-SRSF7^[RA]^) or glutamate (mCherry-SRSF7^[RD]^) residues (Fig. 3f). Expression of the fusion proteins was verified by confocal fluorescence microscopy and Western blot (Additional file 1: Fig. S5C, S5D). As expected, mCherry-SRSF7^[RS]^ and mCherry-SRSF7^[RD]^ localized to the nucleus, where they were enriched in nuclear speckles. However, mCherry-SRSF7^[RA]^ predominantly mislocalized to nucleoli likely due to the high positive charge of the RA domain and was therefore excluded from the Co-IP analyses (Additional file 1: Fig. S5D). Confirming that FIP1 preferentially associates with hypo-phosphorylated SRSF7, GFP-FIP1 co-immunoprecipitated with mCherry-SRSF7^[RD]^ less efficiently than mCherry-SRSF7^[RS]^ (Fig. 3g). Surprisingly, however, CPSF5-GFP co-immunoprecipitated more efficiently with mCherry-SRSF7^[RD]^ than mCherry-SRSF7^[RS]^, suggesting that CPSF5 rather associates with hyper-phosphorylated SRSF7 (Fig. 3h, see also p-SRSF7 in Fig. 3b).

The CFIm complex is a hetero-tetramer of two CPSF5 and two CPSF6/7 subunits [11]. We hypothesized that SRSF7 might replace CPSF6 in a subpopulation of CFIm complexes, because (i) CPSF6 and SRSF7 have similar domain structures, (ii) CPSF6 interaction with CPSF5 also requires its RRM domain, and (iii) CPSF6 also recruits FIP1 via its hypo-phosphorylated RS domain [16, 17]. To test this, we depleted CPSF6 from GFP-FIP1-expressing cell lines and performed Co-IPs. The interaction between FIP1 and SRSF7 was unchanged, while the interaction with CPSF5 was lost (Additional file 1: Fig. S5E-G). This confirmed that CPSF5 interacts with FIP1 via CPSF6 and suggested that SRSF7 and FIP1 interact directly and do not require CFIm.

Taken together, these results suggest that the RS domain of SRSF7 is sufficient for recruiting FIP1. The interaction does not require CFIm or bound RNA but dephosphorylation of SRSF7’s RS domain. In contrast, interaction of SRSF7 with CPSF5 requires its RNA-binding domain and hyper-phosphorylation of its RS domain.

### Two SRSF7-specific protein features promote its interaction with CPA factors

**Fig. 4:**
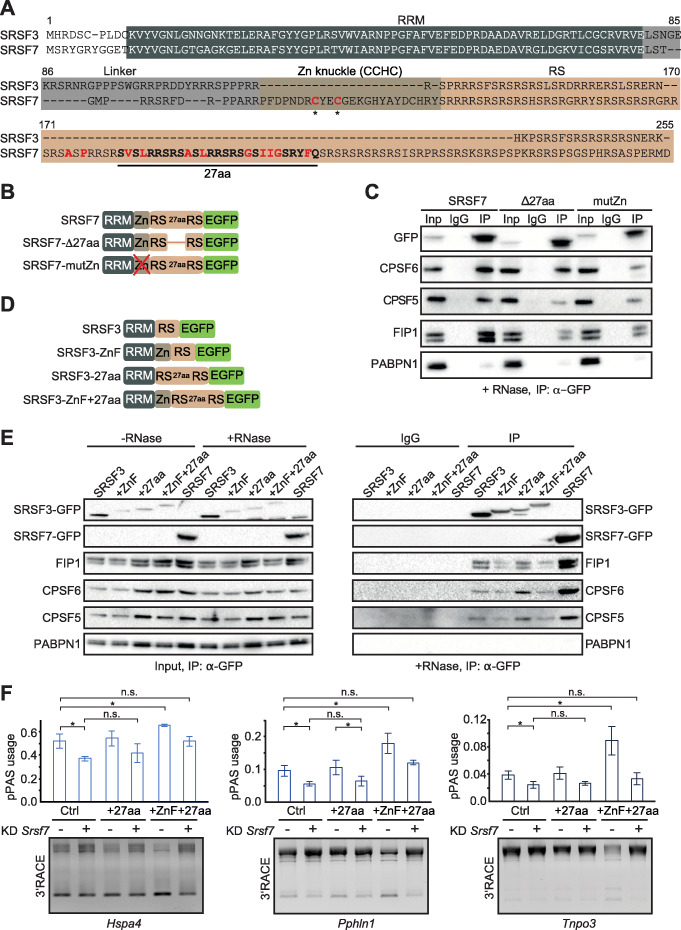
Two SRSF7-specific protein features promote its interaction with CPA factors. **a** Alignment (Clustal Omega) of SRSF3 (amino acids [aa] 1–164) and SRSF7 proteins (aa 1–238). Protein domains are highlighted in dark gray (RRM), light gray (linker domain), olive (CCHC-type Zn knuckle), and light brown (RS domain). The 27 aa stretch is highlighted in bold with hydrophobic residues marked in red. Cysteine residues mutated to alanine in Zn knuckle in SRSF7-mutZn are marked with asterisks. **b** Scheme of GFP-tagged SRSF7 mutants stably expressed in P19 cells. **c** Co-IPs using GFP-tagged SRSF7/SRSF7-Δ27aa/SRSF7-mutZn cells with RNase A treatment: Proteins were pulled-down by α-GFP antibodies and probed for CPA factors using specific antibodies. PABPN1 served as a control for RNA degradation. IP, immunoprecipitation; IgG, unspecific antibody control. **d** Scheme of GFP-tagged SRSF3 chimeras harboring Zn knuckle, 27 aa stretch of SRSF7, or both. **e** Co-IPs using GFP-tagged SRSF3 chimeras: Proteins were pulled-down by α-GFP antibodies and probed for CPA factors using specific antibodies. Lysates were treated with (+RNase) or without (−RNase) RNase A prior to IPs. PABPN1 served as a control for RNA degradation. Inp, input. Samples without RNase A treatment are shown in Fig. S7A. **f** Quantification of 3′RACE-PCRs (*n* = 3) of *Hspa4*, *Pphln1*, and *Tnpo3* transcripts in Ctrl and *Srsf7* knockdown P19 cells transiently overexpressing SRSF3-27aa and SRSF3-27aa + ZnF chimeric proteins. Data are represented as mean ± standard deviation of mean. Student’s *t* test, *P value < 0.05

To understand why SRSF7 interacts directly with CPA factors while SRSF3 does not, we asked whether some features specific to SRSF7 might confer this recruitment. SRSF3 and SRSF7 are closely related and structurally similar, with nearly identical RRM domains [36]. However, SRSF7 contains an additional Zn knuckle for RNA binding [40] and has a longer RS domain, which is interrupted by a stretch of 27 amino acids (27aa) enriched in hydrophobic residues [52] (Fig. 4a). We have recently shown that inclusion of this 27aa stretch is regulated by alternative splicing and promotes oligomerization of SRSF7, leading to the formation of nuclear bodies involved in auto-regulation of SRSF7 expression [41]. Moreover, inactivation of the Zn knuckle in SRSF7 changes its RNA-binding preference from GAY to CNYC, the binding motif of SRSF3 [39, 41]. To assess the impact of these SRSF7-specific protein features (27aa stretch, Zn knuckle) on the interaction with CPA factors, we used stable P19 cell lines expressing GFP-tagged SRSF7 variants either lacking the 27aa stretch (Δ27aa) or containing an inactive Zn knuckle (mutZn) [41] (Fig. 4b). Expression, subnuclear localization and phosphorylation of the SRSF7 variants were verified by Western blot and confocal fluorescence microscopy (Additional file 1: Fig. S6A, B, D).

Strikingly, both mutants interacted much less with CPSF5 and FIP1 as determined by Co-IPs with RNase treatment (Fig. 4c), suggesting that the 27aa stretch and the Zn knuckle both contribute to SRSF7’s interaction with the CPA factors. To test whether these features are sufficient to promote the interactions, we transferred the 27aa stretch and the Zn knuckle (separately and in combination) to the corresponding positions in SRSF3 in a domain-swap experiment (Fig. 4d). The chimeric constructs were transiently transfected into P19 cells, and their expression, subcellular localization, and phosphorylation were verified (Additional file 1: Fig. S6C, E, F). Chimeric SRSF3 proteins were often expressed at much lower levels than wild-type SRSF3-GFP in Co-IP transfection experiments (Fig. 4e), but taking this into account, the Co-IPs suggest that inserting the 27aa stretch alone into SRSF3 (SRSF3-27aa) increases its association with CPSF5, CPSF6, and FIP1 (Fig. 4e, Additional file 1: Fig. S7A). Insertion of the Zn knuckle alone (SRSF3-Zn) or in combination with the 27aa stretch (SRSF3-ZnF + 27aa) did not enhance CPA factor interactions visibly (Fig. 4e, Additional file 1: Fig. S7A). Despite this, transient OE of SRSF3-ZnF + 27aa chimeras increased pPAS usage in SRSF7 APA targets, similar to OE of SRSF7 (Figs. 2e and 4f, Additional file 1: Fig. S7C). When endogenous *Srsf7* was concomitantly depleted, this pPAS enhancement was abolished (Fig. 4f, Additional file 1: Fig. S7B, C). In contrast, OE of SRSF3 containing the 27aa hydrophobic stretch alone had no effect on pPAS usage, indicating that both sequence-specific binding via the Zn knuckle and enhanced FIP1 interaction via the 27aa stretch are required for pPAS activation. Based on these targeted deletions and domain-swap experiments, we conclude that the hydrophobic 27aa stretch and the Zn knuckle, which are absent in SRSF3, contribute to the recruitment of CPA factors and pPAS enhancement and therefore to the functional diversification of SRSF3 and SRSF7 in APA.

### SRSF7 levels decrease and 3′UTRs are globally extended during neuronal differentiation

**Fig. 5:**
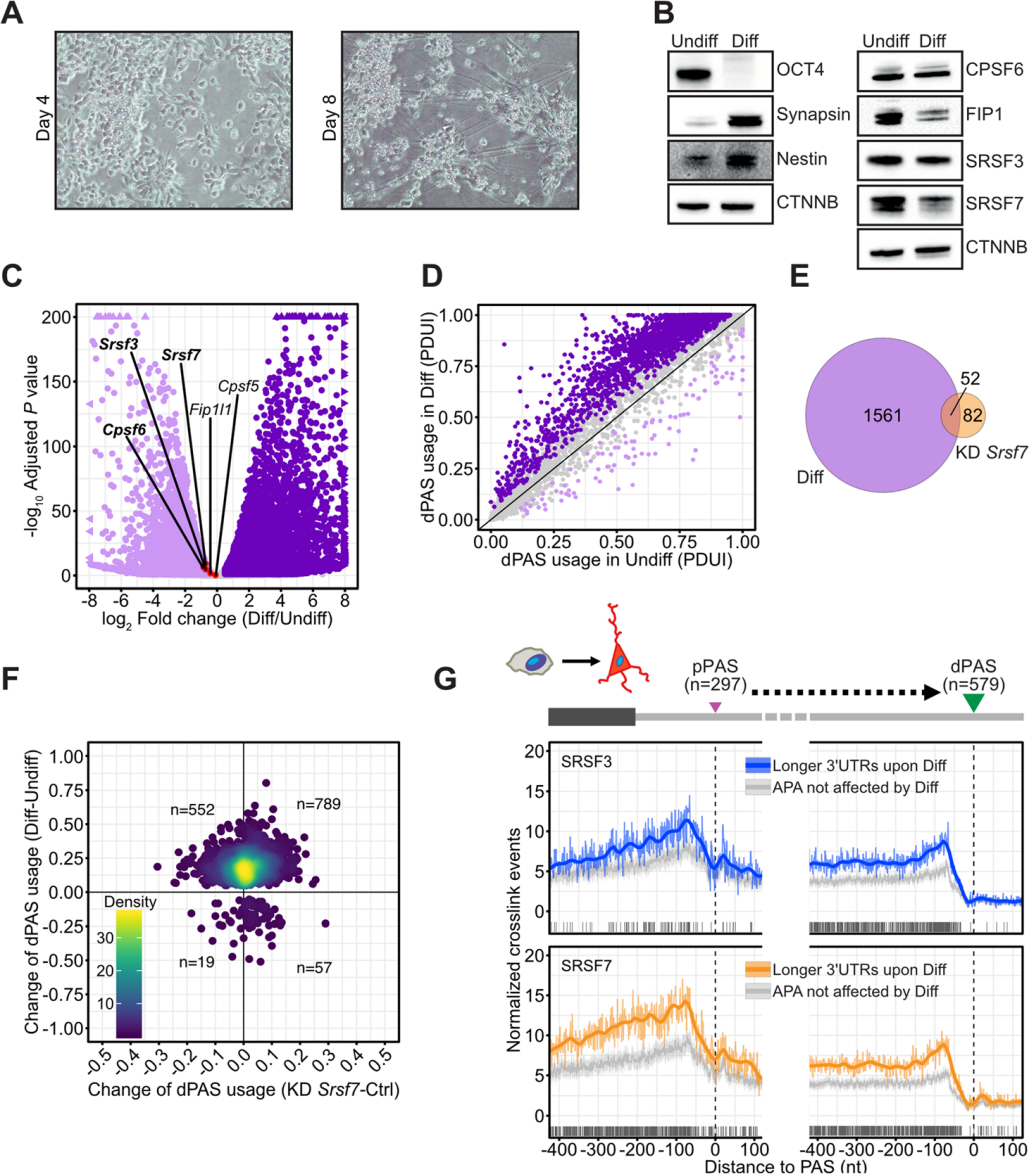
SRSF7 levels decrease and 3′UTRs are globally extended during neuronal differentiation. **a** Morphological changes of P19 cells during neuronal differentiation monitored by bright field microscopy at days 4 and 8 after initiation. **b** Western blot to monitor P19 differentiation using antibodies specific for the pluripotency factor OCT4 and the neuronal markers Synapsin 1 and Nestin. Protein levels of SRSF3, SRSF7, FIP1, and CPSF6 were analyzed using specific antibodies. β-catenin (CTNNB) was used as loading control. **c** Volcano plot of differential gene expression upon P19 differentiation analyzed by DESeq2. Significant genes (adjusted *P* value ≤ 0.1, log_2_-transformed fold change ≥ 0.5) are highlighted in purple. Genes of CPA factors, *Srsf3* and *Srsf7*, are indicated with significant changes highlighted in bold. **d** Scatterplot of PDUIs in undifferentiated and differentiated P19 cells. Genes with significant changes in 3′UTR-APA (adjusted *P* value ≤ 0.1, |ΔPDUI| ≥ 0.05) are highlighted in purple. **e** Venn diagram showing overlap of genes with changes in 3′UTR length upon differentiation or *Srsf7* KD. **f** Scatter plot comparing dPAS usage upon differentiation and *Srsf7* KD for all genes with significant changes in 3′UTR-APA upon differentiation as in **d**, irrespective of their regulation upon *Srsf7* knockdown. Number of genes given in each quadrant, excluding data points on axes. **g** Metaprofiles of normalized iCLIP crosslink events of SRSF3 and SRSF7 around pPASs and dPASs of transcripts with extended 3′UTRs upon differentiation compared to randomly sampled PASs that are not affected. Raw signal and loess smoothing are shown. Significant differences in SRSF3 and SRSF7 binding are computed by a *z*-score approach. Positions with FDR ≤ 0.01 are indicated below

The CPA factor FIP1 was recently implicated in the maintenance of pluripotency and renewal of mouse embryonic stem cells. During differentiation, FIP1 is downregulated with a concomitant global 3′UTR lengthening [27]. To test whether SRSF3 and SRSF7 might also regulate pluripotency via APA, we differentiated pluripotent P19 cells into neuronal cells [53]. On day 8 of differentiation, the cells had adopted the characteristic neuronal morphology reflected by the presence of multiple neurites (Fig. 5a), expression of the neuronal marker Synapsin 1, and complete loss of the pluripotency transcription factor OCT4 (Fig. 5b). Importantly, the protein levels of FIP1 and SRSF7 decreased by ~ 3-fold, while CPSF6 and SRSF3 levels appeared unchanged (Fig. 5b). We performed RNA-seq from undifferentiated (Undiff) and differentiated (Diff) cells (3 replicates) and analyzed the data with DESeq2 [54] and DaPars [43]. *Srsf7* and *Fip1l1* (encoding the protein FIP1) mRNA levels were only slightly decreased in differentiated cells (1.65- and 1.33-fold; Fig. 5c, Additional file 5: Table S4), suggesting that their low protein levels are caused by reduced translation and/or protein stability. In agreement with previous studies [55, 56], we observed a global shift to dPAS usage in differentiated P19 cells (Fig. 5d, Additional file 6: Table S5). This suggested that in pluripotent P19 cells, pPASs are preferentially used. Consistent with SRSF7 being a pPAS-enhancing protein, the majority of transcripts with extended 3′UTRs in differentiated cells showed a similar trend after *Srsf7* KD, although most changes did not pass the significance thresholds of our DaPars analysis (Fig. 5e, f). Importantly, transcripts with extended 3′UTRs in differentiated cells were highly enriched for binding of SRSF7 at their pPASs in undifferentiated cells (Fig. 5g). In contrast, SRSF3 binding was not enriched at these pPASs and the targets seemed anti-correlated after *Srsf3* KD (Fig. 5g, Additional file 1: Fig. S8A).

We conclude that high concentrations of factors that enhance pPAS usage, such as FIP1 and SRSF7, promote their usage under certain conditions, such as pluripotency. The downregulation of these factors in differentiated cells leads to the increased usage of the stronger dPASs and the expression of transcripts with longer 3′UTRs.

### SRSF3 promotes dPAS usage by maintaining high levels of CFIm

Our data so far suggest that SRSF3 might prevent SRSF7 from recruiting CPA factors to some pPASs through competitive binding. But this is likely not the sole mechanism by which SRSF3 regulates 3′UTR-APA, since the number of genes with changes in 3′UTR length is 5-fold higher upon *Srsf3* KD than *Srsf7* KD (Fig. 1c). One possibility would be that SRSF3 regulates 3′UTR-APA also indirectly through modulating the levels of CPA factors.

**Fig. 6:**
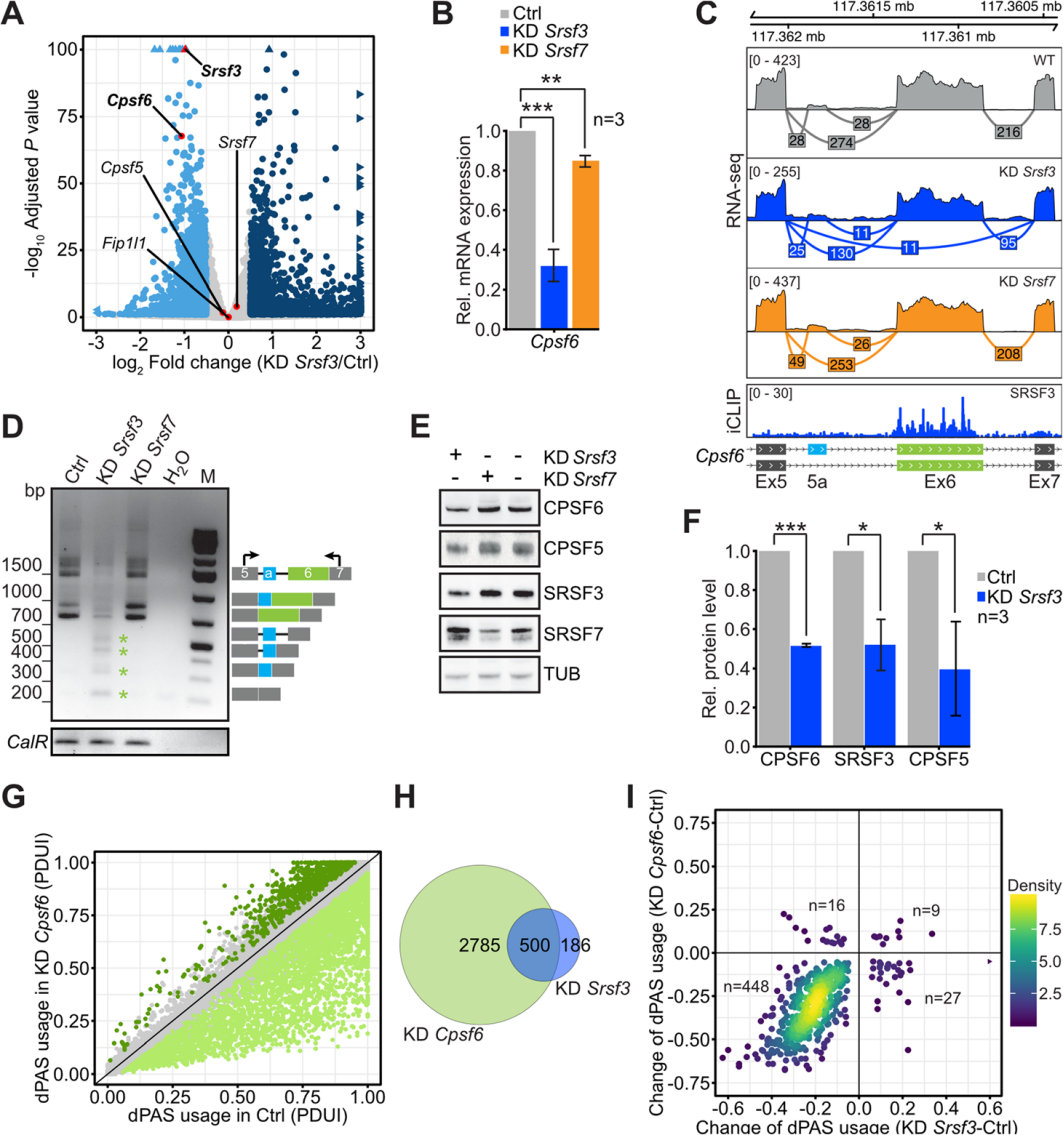
SRSF3 promotes dPAS usage by maintaining high levels of CFIm. **a** Volcano plot of differential gene expression after *Srsf3* KD analyzed by DESeq2. Significant genes (adjusted *P* value ≤ 0.1, log_2_-transformed fold change ≥ 0.5) are highlighted in blue. Genes of CPA factors, *Srsf3* and *Srsf7*, are indicated with significant changes highlighted in bold. **b** Quantification of *Cpsf6* mRNA levels after KD of *Srsf3* and *Srsf7* by RT-qPCR. Data are represented as mean ± stDev. *n* = 3, Student’s *t* test. ***P value < 0.005, **P value < 0.01. **c** Top: Sashimi plots and coverage of RNA-seq reads mapping to *Cpsf6* exons 5 to 7 in control samples (WT, gray) and after KD of *Srsf3* (blue) and *Srsf7* (orange). Bottom: Genome browser view of iCLIP crosslink events of SRSF3 from P19 cells in the same region. Small alternative exon within intron 5 (termed ‘exon 5a’, 111 nt) indicated in light blue. **d** RT-PCR after KD of *Srsf3* and *Srsf7* using primers in exons 5 and 7 of *Cpsf6*. *Cpsf6* isoforms corresponding to the PCR amplicons are indicated beside gel. Shorter exon 6-skipped isoforms are indicated with asterisks. *CalR* amplicons served as control for cDNA integrity. **e** Western blot using P19 cell lysates after KD of *Srsf3* and *Srsf7*. Protein levels of CPSF5, CPSF6, SRSF3, and SRSF7 were assayed using specific antibodies. Alpha-tubulin was used as loading control. **f** Quantification of CPSF6, CPSF5, and SRSF3 protein levels from Western blots. *n* = 3. Student’s *t* test. ***P value < 0.001, *P value < 0.05. **g** Scatterplot of PDUIs in P19 control cells (Ctrl) and after *Cpsf6* KD*.* Genes with significant changes in 3′UTR-APA (adjusted *P* value ≤ 0.1) are highlighted in green. **h** Overlap of genes with shortened 3′UTRs after KD of *Srsf3* or *Cpsf6*. **i** Scatter plot comparing dPAS usage of KD *Srsf3* and *Cpsf6*

To investigate this, we quantified changes in the expression of CPA factors in our RNA-seq data using DESeq2 (Additional file 1: Fig. S8B). Strikingly, *Srsf3* KD reduced the levels of *Cpsf6* transcripts by 2-fold, while no other CPA factor was affected (Fig. 6a). Decreased *Cpsf6* transcript levels were confirmed by RT-qPCR (~ 3-fold; Fig. 6b). *Srsf7* KD had only a minor effect on *Cpsf6* levels and did not affect the levels of any other CPA factor (Fig. 6b, Additional file 1: Fig. S8B).

To investigate how SRSF3 depletion decreases *Cpsf6* mRNA levels, we analyzed its splicing pattern using RNA-seq data (Fig. 6c). We observed multiple splicing alterations within the *Cpsf6* transcripts upon *Srsf3* KD, such as increased inclusion of a small alternative exon within intron 5 (termed ‘exon 5a’, 111 nt), the skipping of exon 6, and the retention of intron 6 (Fig. 6c). Transcripts including exon 5a likely encode an alternative CPSF6 protein isoform of 72 kDa (CFIm-72) whose functional difference from the main isoform of 68 kDa (CFIm-68) is not known. Skipping of exon 6 introduces a frameshift resulting in non-productive transcripts. iCLIP data showed that SRSF3 bound massively to exon 6, suggesting that it directly promotes its inclusion (Fig. 6c).

The appearance of various shorter *Cpsf6* transcript isoforms with skipped exon 6 after *Srsf3* KD was confirmed using semi-quantitative RT-PCR and sequencing (see asterisks in Fig. 6d). This was accompanied by a drastic reduction in the levels of both transcript isoforms encoding full-length CPSF6 proteins (68 and 72 kDa). Importantly, *Srsf3* KD—but not *Srsf7* KD—also resulted in ~ 2-fold decreased CPSF6 protein levels (Fig. 6e, f). CPSF5 was downregulated to a similar extent, in line with previous studies reporting that both proteins stabilize each other [11, 14].

In human cells, it was shown that CFIm binds preferentially upstream of dPASs and enhances their usage through a direct recruitment of FIP1 [17]. As a result, CPSF5 and CPSF6 depletion both lead to a global switch to pPAS usage [29, 30]. RNA-seq after KD of *Cpsf6* from mouse P19 cells also revealed a globally enhanced pPAS usage (Fig. 6g, Additional file 1: Fig. S8C, Additional file 8: Table S7) and clearly mimicked *Srsf3* KD by affecting the majority of SRSF3 APA targets in the same way (457 out of 686, 67%; Fig. 6h, i). Yet, a far greater number of transcripts got shorter 3′UTRs upon *Cpsf6* KD than *Srsf3* KD (2711 vs. 579; Additional file 1: Fig. S8D), presumably due to a more efficient depletion of CPSF6 upon *Cpsf6* KD compared to *Srsf3* KD (80% vs. 50% depletion). The similar regulation of 3′UTR-APA by SRSF3 and CPSF6 was validated for three target genes by 3′RACE-PCR (Additional file 1: Fig. S8E).

To confirm that SRSF3 affects 3′UTR length indirectly via CPSF6, we transiently overexpressed CPSF6-myc in SRSF3-depleted cells to rescue CPSF6 levels (Additional file 1: Fig. S8F). 3′RACE-PCR revealed that 3′UTR shortening of *Ddx21* and *Anp32e* could not be rescued by ectopic CPSF6 expression, suggesting that these transcripts are direct SRSF3 targets. *Rab11a*, *Phpln1*, and *Tnpo3* reacted to CPSF6 OE with increased dPAS usage, which partially counteracted the 3′UTR shortening triggered by the *Srsf3* KD (Additional file 1: Fig. S8G). However, the effects were small and the rescue was incomplete, likely because the CPSF5 levels remained low in the transient CPSF6 OE (Additional file 1: Fig. S8F).

These observations argue for two distinct modes of 3′UTR-APA regulation by SRSF3. On the one hand, SRSF3 inhibits pPAS usage directly through competitive binding with pPAS enhancer proteins. On the other hand, SRSF3 indirectly promotes dPAS usage by ensuring the productive splicing of mRNAs encoding CPSF6, a key component of the dPAS enhancer CFIm.

### CFIm inhibits pPAS usage through unproductive FIP1 recruitment in SRSF3-regulated transcripts

**Fig. 7:**
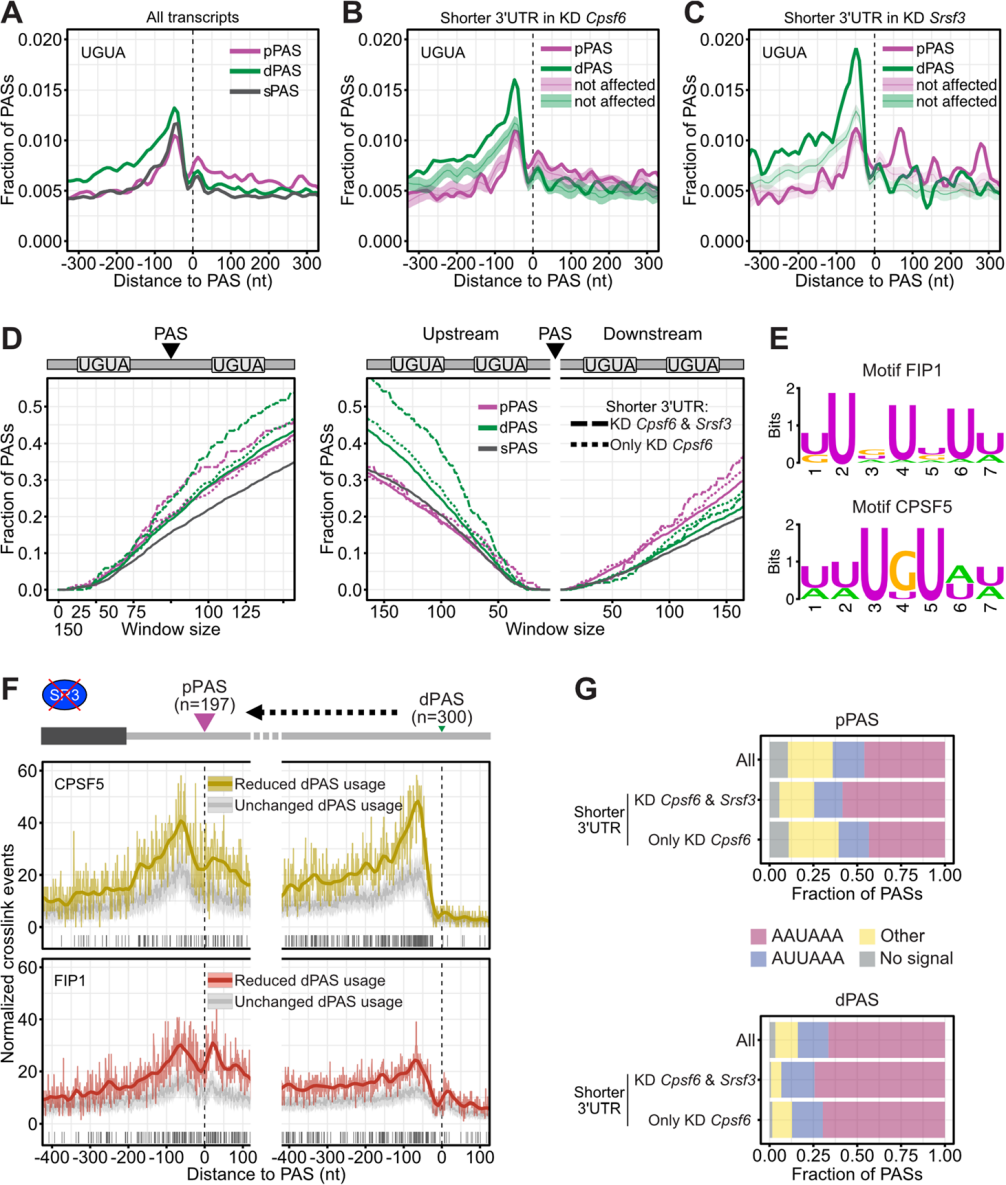
CFIm inhibits pPAS usage through unproductive FIP1 recruitment in SRSF3-regulated transcripts. **a** Enrichment of UGUA motifs around sPASs, pPASs, and dPASs used in P19 cells. **b** UGUA motif enrichment around the pPAS and dPAS in CPSF6 targets and non-affected transcripts. **c** UGUA motif enrichment around the pPAS and dPAS in SRSF3 targets and non-affected transcripts. **d** Cumulative fraction of dual UGUA motifs upstream, downstream, and enclosing sPAS, pPAS and dPAS. **E** Binding motifs of FIP1 and CPSF5 derived from iCLIP in P19 cells. **f** Metaprofiles of normalized crosslink sites of CPSF5 and FIP1 around the pPAS and dPAS of SRSF3 targets compared to random PAS. Significant differences in CPSF5 and FIP1 binding are computed by a z-score approach. Positions with FDR ≤ 0.01 are indicated below. **g** Stacked bar charts showing the proportion of pPASs (upper panel) and dPASs (lower panel) that are associated with known CSE motifs. SRSF3-regulated 3′UTRs are compared to CPSF6-only targets and all 3′UTRs

SRSF3 3′UTR-APA targets appear especially sensitive to reduced levels of CPSF6. To better understand their regulation by CFIm, we analyzed some of their features. We first compared CFIm binding motifs at pPASs and dPASs, since CFIm binding to UGUA motifs upstream of dPASs (− 80 nt to − 40 nt) had been shown to enhance their usage [17]. In line with this, CPSF6 targets had a greater tendency than all transcripts to harbor at least one UGUA motif upstream of their dPAS (Fig. 7a, b, Additional file 1: Fig. S9A). But this tendency was even higher for SRSF3 targets, suggesting that they rely on CFIm to enhance dPAS usage (Fig. 7c).

Strikingly, in SRSF3 targets, UGUA motifs appeared equally distributed on either side of the pPASs, with a strong enrichment around 65 nt downstream. Given that each of the two CPSF5 subunits in a CFIm complex can bind one UGUA motif while the intervening RNA loops around the CFIm heterotetramer [15, 57], this raised the intriguing possibility that CFIm blocks these pPASs by excluding some of the sequences necessary for their activation such as USEs, CSEs, and DSEs. In line with this notion, we found that SRSF3 targets indeed had a greater tendency to have UGUA pairs flanking their pPASs compared to CPSF6 targets and all transcripts (Fig. 7d, Additional file 1: Fig. S9B). SRSF3 targets also more frequently harbored UGUA pairs downstream of their pPASs, which could inhibit their usage, for instance by excluding DSEs [15, 57]. Finally, the SRSF3 targets also showed a much greater tendency to contain UGUA pairs upstream of their dPASs, which should enhance dPAS usage [17]. Altogether, the UGUA motif distribution suggests that SRSF3 targets are especially sensitive to reduced CFIm levels because CFIm might play a dual role on these transcripts: it activates their dPAS and at the same time blocks their pPAS.

To assess whether the UGUA motif distribution is reflected by CFIm binding and FIP1 recruitment, we performed iCLIP of GFP-FIP1 and CPSF5-GFP from P19 cell lines (6–7 replicates; Additional file 1: Fig. S10A, S10B). Merging replicates, we obtained 1,851,266 unique crosslink events for CPSF5 and 3,759,237 for FIP1 (Additional file 1: Table S8). Pentamer enrichment analysis retrieved the expected binding motifs—UGUA for CPSF5, and UG-rich sequences for FIP1 (Fig. 7e). Indeed, crosslinking of both CPSF5 and FIP1 mirrored the UGUA motif distribution, with peaks directly upstream of pPASs and dPASs, in line with previous studies [14, 29]. But they also displayed an additional peak downstream of pPASs, which has not been reported previously (Additional file 1: Fig. S10C). Importantly, binding of both CPSF5 and FIP1 was strongly enriched on either side of pPASs and upstream of dPASs in SRSF3 targets, whereas CPSF6 targets showed only a slight enrichment for both proteins upstream of dPASs (Fig. 7f, Additional file 1: Fig. S10D). Given that many pPASs in SRSF3 targets are suppressed under normal conditions (where iCLIP was performed), recruitment of FIP1 to those pPASs might be unproductive, i.e., does not result in cleavage. To confirm this, we divided all transcripts with long 3′UTRs (unused pPAS) and short 3′UTRs (used pPAS) in control cells and compared their binding patterns of FIP1 and CPSF5 (Additional file 1: Fig. S10E). Interestingly, both CPSF5 and FIP1 bound downstream of suppressed pPASs, suggesting a mechanism of pPAS suppression through unproductive CFIm and FIP1 binding. Our data suggested that for many CPSF6 APA targets the promotion of dPAS usage is sufficient to generate transcripts with long 3′UTRs. However, in a subset of these (i.e., SRSF3 APA targets), additional mechanisms appear to be used to also suppress pPAS usage.

To test whether those suppressed pPASs are stronger than usual, we compared the frequency of CSE variants. Indeed, SRSF3 APA targets more often harbored the two main CSE variants (AAUAAA and AUUAAA) at their pPASs compared to CPSF6 APA targets (75% vs. 60%; Fig. 7g). Although the same was true for their dPASs (95%), pPASs are transcribed first; hence, they need to be suppressed to generate transcripts with long 3′UTRs.

Altogether, our data provide evidence for two modes by which CFIm can regulate 3′UTR-APA. CFIm binding can either inhibit or enhance PAS usage depending on the positioning of UGUA motifs and CFIm binding. CFIm complexes bound to suppressed pPASs still recruit FIP1 and likely other CPA factors, but cleavage does not occur, suggesting that the assembled cleavage complexes are inactive. Indirect SRSF3-regulated targets are more sensitive to low CFIm levels because they rely on pPAS inhibition and dPAS enhancement by CFIm to ensure the generation of long 3′UTRs. Additional pPAS inhibition might be necessary because SRSF3-sensitive pPASs are stronger compared to CPSF6-only targets, which do not show this dual CFIm dependency.

## Discussion

The usage of alternative PASs can be modulated, either positively or negatively, by regulatory RBPs. RBPs either directly compete with core CPA factors for binding to regulatory elements or they bind to sequences outside of the PAS region (reviewed in [19, 22, 58]). We show here that SRSF3 and SRSF7 modulate PAS usage directly by binding upstream of regulated pPASs. Despite being very closely related, SRSF3 and SRSF7 affect 3′UTR length in opposite directions. This is due to slight differences in the domain architecture of both SR proteins. We find that the direct modulation of pPAS usage by SRSF3 and SRSF7 requires CSEs of intermediate strength, is independent of splicing and occurs in a concentration- and binding-dependent manner. In addition, SRSF3 affects 3′UTR-APA also indirectly, by maintaining high levels of CFIm through alternative splicing of the *Cpsf6* mRNA. Based on the data presented here, we propose the following model for 3′UTR-APA modulation by SRSF3 and SRSF7.

**Fig. 8:**
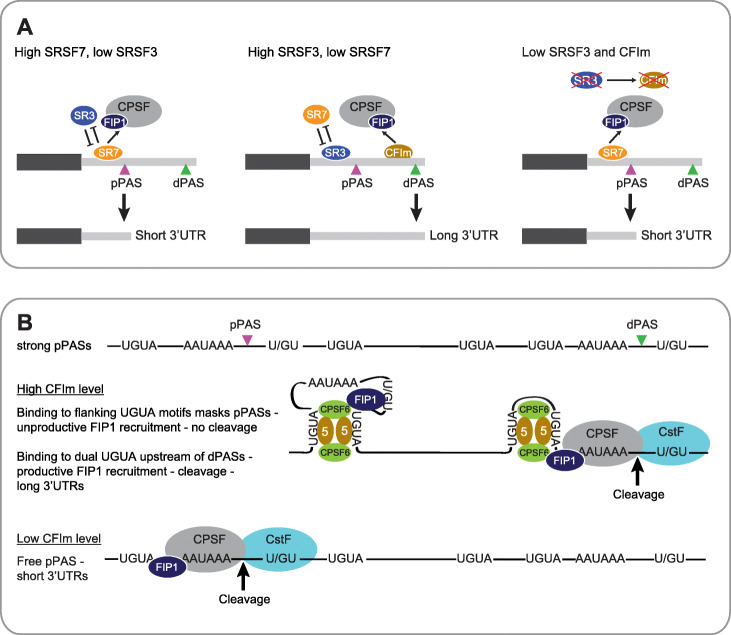
Model of pPAS usage regulated by SRSF7, SRSF3, and CFIm. **a** Model of regulation of pPAS usage and 3′UTR-APA by SRSF7 and SRSF3. Left panel: High SRSF7 levels and binding upstream of pPASs enhance pPAS usage through the recruitment of FIP1—transcripts with short 3′UTRs. Middle panel: High SRSF3 levels and binding upstream of pPASs prevent SRSF7 binding and pPAS enhancement. High levels and binding of CFIm upstream of dPASs enhances dPAS usage instead—transcripts with long 3′UTRs. Right panel: *Srsf3* KD results in reduced CFIm levels. The dPAS is no longer enhanced by CFIm binding, instead SRSF7 binding at pPASs enhances their usage – transcripts with short 3′UTRs. **b** Model of pPAS inhibition by CFIm. Top: CFIm binding immediately up- and downstream of the pPAS inhibits its usage by looping out the CSEs or DSEs, leading to unproductive FIP1 recruitment – no cleavage – transcripts with long 3′UTRs. 5—CPSF5. Bottom: Depletion of SRSF3 or CPSF6 leads to low CFIm levels, which unmasks pPASs and allows their activation—transcripts with short 3′UTRs.

When SRSF7 is present at high levels, such as in pluripotent cells or certain cancer cells [59], it binds upstream of pPASs and recruits FIP1, thereby acting as a sequence-specific enhancer of pPAS usage and promoting transcripts with short 3′UTRs (Fig. 8a, left panel). SRSF3 also binds upstream of pPASs but, unlike SRSF7, it cannot recruit FIP1. Thus, SRSF3 binding prevents activation of these pPASs by impairing the association of SRSF7. When SRSF7 levels are low, e.g., in differentiated cells, increased SRSF3 binding to pPASs favors the usage of dPASs and the generation of long 3′UTRs (Fig. 8a, middle panel). When SRSF3 levels are low, as it occurs for example in progressive liver disease [60], SRSF7 can bind more to pPASs. In addition, the levels of CFIm decrease due to unproductive splicing of *Cpsf6*, which prevents the activation of dPASs. Together, this favors pPAS usage and the generation of transcripts with short 3′UTRs (Fig. 8a, right panel).

Apart from only preventing the association of SRSF7 with pPASs, SRSF3 binding might also actively inhibit their usage, similar to U1 snRNP [61, 62]. U1 snRNP inhibits polyadenylation through the inactivation of PAP by U1-70K and U1A, which both contain a PAP-inhibitory domain (PID) [63]. Interestingly, SRSF3 also contains a predicted PID within its RS domain and might inhibit PAP in these inactive CPA complexes [64], but further studies are required to test this hypothesis.

We show that FIP1 recruitment by SRSF7 occurs via direct protein-protein interaction. It is independent of CFIm, but dependent on the Zn knuckle and the hydrophobic 27aa stretch within the RS domain of SRSF7, which are absent in SRSF3. The interaction requires the RS domain of SRSF7 to be hypo-phosphorylated, but is independent of its RNA-binding domain, similar to what was shown for CPSF6 [17]. This means that SRSF7 can simultaneously bind to pPASs in pre-mRNAs and to FIP1 and thereby recruit FIP1 to bound pPASs, e.g., those without UGUA motifs like the *Ddx21* 3′UTR. Dephosphorylation of SRSF7 might occur during CPA, since our reporter experiments suggest that 3′UTR-APA regulation by SRSF7 is independent of splicing. In line with this, the SR protein phosphatase PP1 was also found in purified CPA complexes [65, 66].

We also find that indirectly SRSF3-regulated pPASs are more sensitive to reduced CFIm levels. This might be due to the fact that these pPASs are stronger than average pPASs and contain mostly the canonical CSE variants (Figs. 1h and 7g) [24, 47]. Since pPASs are transcribed first, strong pPASs should in principle be used by default when the core CPA machinery is present in sufficient levels. However, most of these SRSF3 targets do harbor long 3′UTRs in P19 cells under normal conditions. This suggests that in addition to the enhancement of their dPASs, their pPASs must also be inhibited.

Three models have been proposed for CFIm-mediated pPAS suppression. (i) CFIm binds to UGUA motifs that overlap with the cleavage site, thereby preventing PAS usage [17, 67]. (ii) CFIm binds one UGUA motif upstream of a pPAS and a second UGUA upstream of a dPAS, thereby looping out a big part of the 3′UTR [15, 29]. (iii) CFIm binds to suboptimal target sites, e.g., non-UGUA motifs, that are more prevalent at pPASs, which would block productive CPSF recruitment [14]. Only the first model was experimentally verified [17, 67].

Our data suggest a fourth mode by which CFIm can inhibit pPAS usage when the pPAS is enclosed by or directly followed by a pair of UGUA motifs. This is in line with the second model implying that large regions of the 3′UTR including the pPAS loop around the CFIm tetramer [15, 29]. Our data suggest that CFIm binding at pPASs can also hide short 3′UTR regions that are important for pPAS activation, e.g., CSEs, DSEs, or the cleavage site (Fig. 8b). This is based on our findings that (i) SRSF3-sensitive pPASs are often flanked or followed by UGUA pairs, (ii) SRSF3-sensitive pPASs have enriched CPSF5 crosslinks flanking them, and (iii) these pPASs are used with low efficiency under the conditions where iCLIP was performed, but become activated when SRSF3 and CFIm levels are reduced. This implies that for some transcripts CFIm plays a dual role in promoting the generation of long 3′UTRs; it binds to pairs of UGUA motifs upstream of dPASs and enhances their usage by recruiting FIP1 [17] (Fig. 8a, middle panel), but it also binds to UGUA pairs downstream of or enclosing strong pPASs to inhibit their cleavage (Fig. 8b). This differential sensitivity of pPASs to fluctuations in CFIm levels provides cells with the possibility to regulate 3′UTR-APA of specific subsets of transcripts, e.g., to regulate senescence [33].

Surprisingly, we found that FIP1 and likely other CPA factors are also recruited to unused pPASs, likely via CFIm, but cleavage does not occur at these sites. Thus, these pPAS-bound CPA complexes must be inactive. In favor of this model, it was recently shown that in HSV-1-infected cells, the immediate early protein ICP27 interacts with FIP1 and induces the assembly of a dead-end 3′ processing complex on the mRNA [68]. Moreover, the U1 snRNP complex that assembles at intronic PASs to suppress premature polyadenylation also contains all CPA factors including FIP1 and CFIm, but cleavage also does not occur at these sites [69]. This suggests that the particular configuration of CFIm bound to suppressed pPASs causes the assembly of inactive CPA complexes that are unable to complete cleavage and polyadenylation, but they protect pPASs from recognition by pPAS enhancers (Fig. 8b). One possibility would be that suppressive pPAS CFIm complexes contain CPSF7, similar to suppressive U1-CPA complexes that are enriched in CPSF7 [69]. CPSF7 does not enhance CPA and its binding sites are much less predictive of PAS usage than CPSF6 binding [14, 29]. Moreover, CPSF7 has a slightly different mode of RNA binding than CPSF6 and does not interact with the pPAS enhancer SRSF7 [70]. Finally, depletion of CPSF7 does not cause a global switch to pPAS usage in HEK293 cells [13, 69]. Although this remains to be confirmed in other cellular systems, our data suggest that CPSF6 regulates fewer targets through pPAS inhibition than through dPAS enhancement.

Our study identifies SRSF3 as a critical regulator of CFIm levels. We show that SRSF3 binds massively to exon 6 in the *Cpsf6* pre-mRNA and enhances its inclusion. Upon *Srsf3* depletion, exon 6 is skipped and the resulting transcripts containing premature stop codons are unstable. Low levels of functional *Cpsf6* transcripts also reduce the levels of CPSF6 protein. Moreover, we find that CPSF5 is co-depleted, indicating that both proteins need to form a tetramer to stabilize each other [11, 14].

Both SRSF3 and SRSF7 were previously identified as interactors of CPSF6 using its RS-like domain in a yeast two-hybrid screen [16]. We confirm here the protein-protein interaction between CFIm and SRSF7 in P19 cells, but its interaction with SRSF3 requires simultaneous binding to the same (pre-)mRNAs. The interaction of CFIm with SRSF7 seems to be different from its interaction with FIP1. Here, binding requires the RNA-binding domain of SRSF7 and RS domain hyper-phosphorylation. This implies that SRSF7 cannot interact with CFIm when it is bound to RNA, and hence cannot recruit CFIm to pPASs. But given that SRSF7 and CPSF6 have a very similar domain structure, hyperphosphorylated SRSF7 might form heterotetramers with CPSF6 and CPSF5, whose function remains to be determined.

SRSF7 was previously implicated in the enhancement of polyadenylation of subgenomic transcripts of retroviruses, such as human immunodeficiency virus (HIV) and Rous sarcoma virus (RSV) [71, 72]. We show here that SRSF7 also enhances pPAS usage in cellular mRNAs in a sequence-specific and concentration-dependent manner through the recruitment of FIP1. This is reminiscent of its functions in splicing, where sequence-specific binding of SRSF7 is required to recruit the spliceosome to splice sites to enhance their usage in a concentration-dependent manner [39]. The apparent discrepancy between the considerable pPAS and FIP1 binding of SRSF7 and relative few APA targets upon *Srsf7* KD is likely due to the partial redundancy of SR proteins that bind to purine-rich motifs, e.g., SRSF6 and SRSF1, which have also been suggested to enhance PAS usage [32, 71, 73].

## Conclusion

Altogether, our data reveal novel mechanistic insights into the direct and indirect regulation of 3′UTR-APA by SRSF3 and SRSF7 in opposite directions, and into how CFIm regulates pPAS usage. Binding of SRSF3 at suppressed pPASs and binding of SRSF7 at activated pPASs followed by NXF1 recruitment might sort mRNAs with short and long 3′UTRs into distinct export-competent mRNPs. These mRNPs could follow distinct routes in the cytoplasm, for example being transported at different subcellular locations for their translation.

## Methods

### Generation and cultivation of stable BAC P19 cell lines

Murine P19 WT cells were purchased (Sigma-Aldrich) and grown under humidified conditions at 5% CO_2_ and 37 °C in DMEM GlutaMAX (ThermoFisher Scientific), supplemented with 100 U/ml Penicillin-Streptomycin (ThermoFisher Scientific) and 10% (v/v) heat inactivated fetal bovine serum (ThermoFisher Scientific), on dishes coated with 0.1% gelatine-PBS (Sigma-Aldrich). Mouse BACs harboring GFP-tagged *Nudt21, Cpsf6* or *Fip1l1* genes were isolated from *Escherichia coli* DH10 cells using the NucleoBond PC 20 kit (Macherey-Nagel). P19 cells were transfected with 1 μg purified BAC DNA using Effectene Transfection Reagent (Qiagen). Cells with stably integrated BACs were selected with 500 μg/ml Geneticin (G418, ThermoFisher Scientific) and regularly checked for mycoplasma contaminations.

### P19 differentiation

P19 wild type cells were differentiated into neuronal cells using retinoic acid according to [53]. Briefly, 10 cm culture dishes were coated with 10 μg laminin diluted in 4 ml PBS overnight. Laminin solution was removed and the dishes were washed tree times with 1x PBS (Sigma-Aldrich) before seeding P19 cells into 10 ml Gibco™ DMEM/F-12, GlutaMAX™ (ThermoFischer Scientific), supplemented with 1x Gibco™ N-2 Supplement (ThermoFischer Scientific) and 100 U/ml penicillin-streptomycin (ThermoFischer Scientific). To start the differentiation the medium was supplemented with 10 ng/ml FGF8 (Sigma-Aldrich), 10 μM DAPT (Sigma-Aldrich) and 500 mM retinoic acid (Sigma-Aldrich). Cells were grown under humidified conditions at 5% CO_2_ at 37 °C. After 4 days, synaptogenesis was induced with 10 ml Gibco™ CTS™ Neurobasal® Medium (ThermoFischer Scientific) supplemented with 1x Gibco™ B-27™ Supplement (ThermoFischer Scientific). To remove all dividing cells, 8 μM Cytosine β-D-arabinofuranoside hydrochloride (Ara-C, Sigma-Aldrich) was added to the cultures. Cells were grown for another 4 days and fully differentiated cells were harvested at day 8. Differentiation progress was monitored every second day by bright field microscopy.

### Co-IPs, Western blot and antibodies

For Western blot experiments, protein concentrations were measured using Bradford 1x Dye Reagent (Bio-Rad) on a NanoDrop2000 (Thermo Scientific). Protein lysates were mixed with 5x Laemmli buffer, boiled at 95 °C for 5 min and 10–20 μg total protein per lane were separated either on homemade 10% SDS-PAGE (Bio-Rad) or on NuPAGE 4–12% Bis-Tris PAGE (ThermoFisher Scientific) gel electrophoresis. Proteins were transferred onto 0.1 μm nitrocellulose membrane (GE Healthcare). The transfer was evaluated by staining with Ponceau S (Amresco).

For Co-IPs, approximately 4 × 10^7^ P19 cells were lysed using NET-2 buffer (150 mM NaCl, 0.05% NP-40, 50 mM Tris-HCl pH 7.5), supplemented with EDTA-free cOmplete Protease Inhibitor Cocktail (Sigma-Aldrich) and 10 mM β-glycerophosphate (Fluka BioChemica) and sonicated on ice (Branson). Lysates were cleared, split in two, and treated with or without 100 μg/ml RNase A for 20 min at 21 °C. 0.2% of total lysate served as input. 10 μg of goat IgG (Sigma-Aldrich) or goat α-GFP (provided by D. Drechsel, MPI-CBG, Dresden, Germany) were pre-incubated with Gammabind G Sepharose beads (GE Healthcare) for 2 h at 4 °C. Subsequently, they were mixed with RNase A-treated or untreated lysates and incubated for 1.5 h at 4 °C. The beads were washed and co-precipitated proteins were eluted with 1.32x NOVEX sample mix with 1/10 reducing agent (ThermoFisher Scientific). Proteins were separated on NuPAGE 4–12% Bis-Tris gels (ThermoFisher Scientific), blotted on nitrocellulose membranes (GE Healthcare), and probed with the following antibodies: rabbit α-CTNNB (Abcam ab2365), rabbit α-CPSF6 (Abcam ab99347), mouse α-FIP1L1 (Santa Cruz Biotechnology sc-398392), goat α-GFP (MPI-CBG), mouse α-CPSF5 (NUDT21; Santa Cruz Biotechnology sc-81109), rabbit α-PABPN1 (Abcam ab75855), mouse α-phosphoSR (mAb104, kindly provided by K. Neugebauer), mouse α-SRSF3 (m7B4, kindly provided by K. Neugebauer), rabbit α-SRSF7 (Assay Biotechnology C18943), rabbit α-mCherry (Invitrogen PA5-34974), and rabbit α-Tet-Repressor (MoBiTec TET01). Donkey α-mouse IgG-HRP (AP192P, Sigma-Aldrich), donkey α-rabbit IgG-HRP (AP182P, EMD Millipore), donkey α-goat IgG-HRP (AB324P, Sigma-Aldrich), and goat α-mouse IgM-HRP (A8786, Sigma-Aldrich) secondary antibodies were used respectively for immuno-detection in combination with ECL Prime Western Blotting Detection Reagent (GE Healthcare). Western blots were additionally probed with the following antibodies: rabbit α-tubulin (Abcam ab176560) and mouse α-GAPDH (Santa Cruz Biotechnology sc-32,233).

### Shrimp alkaline phosphatase treatment

Total protein was extracted from approximately 1 × 10^7^ P19 cells as described before using ice-cold NET2 buffer with 10 mM MgCl_2_ supplemented with EDTA-free cOmplete Protease Inhibitor Cocktail (Sigma-Aldrich) and 10 mM β-glycerophosphate (Fluka BioChemica). Protein concentrations were measured using Bradford 1x Dye Reagent (Bio-Rad) on a NanoDrop2000 (Thermo Scientific). Ten micrograms total protein was added to two 1.5 ml reaction tubes and mixed with either − SAP-Mix (1x CutSmart Buffer [New England Biolabs], 10 mM beta-phosphoglycerate) or + SAP-Mix (1x CutSmart Buffer [New England Biolabs], 5 U rSAP [New England Biolabs]). The volume was adjusted to 20 μl using NET2/MgCl_2_-buffer and the samples were incubated for 30 min at 37 °C with agitation (300 rpm). Subsequently, the samples were mixed with 5x Laemmli buffer, with and without glycerol, boiled at 90 °C for 5 min, and subjected to Western blot.

### SRSF3 immunoprecipitation, sample preparation, and quantitative mass spectrometry (TMT)

P19 cells stably expressing SRSF3-GFP at physiological levels [32] or nuclear GFP (GFP-NLS) as control [48] were grown in 14-cm dishes and harvested after washing with ice-cold 1x PBS. Immunoprecipitations (IPs) were performed as described above. The IPs were eluted three times from magnetic beads with each 50 μl 6 M Guanidin-Hydrochloride (in Tris pH 8.0) at room temperature (RT). Eluted IP-Samples were reduced with DTT (final 5 mM) for 30 min at 55 °C and alkylated using chloroacetamide (final 15 mM) for 30 min in the dark at RT. The reaction was quenched by DTT (final 10 mM) for 15 min at RT. Samples were cleaned by methanol chloroform precipitation and dried protein pellets were taken up in 0.2 M EPPS pH 8.2 and 10% acetonitrile (ACN). For digestion, 0.4 μg trypsin (Promega) was added and the samples were incubated over night at 37 °C. The amount of ACN was adjusted to 20% and peptides were incubated with 20 μg of TMT-reagents (ThermoFisher Scientific) for 1 h at RT. TMT-labeling reaction was quenched by addition of hydroxylamine to a final concentration of 0.5% for 15 min at RT. Samples were pooled, acidified, and dried for further processing. Peptides were cleaned by stage tip using Empore C18 (Octadecyl) resin material (3 M Empore) and taken up in 0.1% formaldehyde and analyzed by LC-MS^2^ using an Easy nLC 1200 (ThermoFisher Scientific) unit with a 22 cm long, 75 μm ID fused-silica column, which has been packed in house with 1.9 μm C18 particles (ReproSil-Pur, Dr. Maisch), and kept at 45 °C using an integrated column oven (Sonation). Peptides were eluted with a non-linear gradient from 5 to 38% ACN over 120 min and directly sprayed into a QExactive HF mass-spectrometer equipped with a nanoFlex ion source (ThermoFisher Scientific) at a spray voltage of 2.3 kV. Full scan MS spectra (350–1400 m/z) were acquired at a resolution of 120,000 at m/z 200, a maximum injection time of 100 ms, and an AGC target value of 3 × 10^6^. Up to 20 most intense peptides per full scan were isolated using a 1 Th window and fragmented using higher energy collisional dissociation (normalized collision energy of 35). MS/MS spectra were acquired with a resolution of 45,000 at m/z 200, a maximum injection time of 80 ms and an AGC target value of 1 × 10^5^. Ions with charge states of 1 and > 6 as well as ions with unassigned charge states were not considered for fragmentation. Dynamic exclusion was set to 20 s to minimize repeated sequencing of already acquired precursors.

### Analysis of mass spectrometry (MS) data

Raw data were analyzed using Proteome Discoverer (PD) 2.4 software (ThermoFisher Scientific). Files were recalibrated using the *Mus musculus* SwissProt database (TaxID = 10090, v. 2017-07-05) with methionine oxidation (+ 15.995) as dynamic modification and carbamidomethyl (Cys,+ 57.021464), TMT6 (N-terminal, + 229.1629) and TMT6 (+ 229.1629) at lysines as fixed modifications. Spectra were selected using default settings and database searches were performed using SequestHT node in PD. Database searches were performed against trypsin digested *Mus musculus* SwissProt database and FASTA files of common contaminants (“contaminants.fasta” provided with MaxQuant) for quality control. Fixed modifications were set as TMT6 at lysine residues, TMT6 (N-terminal), and carbamidomethyl at cysteine residues. As dynamic modifications acetylation (N-terminal) and methionine oxidation were set. After search, posterior error probabilities were calculated and PSMs filtered using Percolator using default settings. The Consensus Workflow for reporter ion quantification was performed with default settings. Results were then exported to Excel and protein levels were normalized to GFP (UniProtKB - P42212).

### Cloning and transfection

#### TetR cloning

Fusion constructs of the tetracycline repressor (TetR) protein and the RS domains of SRSF3 and SRSF7 as well as their phosphomimetics were generated via restriction and ligation cloning. For the phosphomimetics, each serine was replaced by either alanine or aspartic acid residues. The sequences were purchased as gBlocks DNA fragments (Integrated DNA Technologies) and amplified using specific primers (Additional file 1: Table S9) to add overhangs including *Xma*I restriction sites. The plasmid containing a single-chain TetR gene and the PCR amplicons were digested using *Xma*I (New England Biolabs) and ligated in a 1:6 ratio using T4 DNA Ligase (Promega).

Multiple sequence alignments to identify domain boundaries were performed using Clustal Omega (https://www.ebi.ac.uk/Tools/msa/clustalo/).

#### mCherry/eGFP cloning

Fusion constructs of the complete coding sequence of *SRSF3* and *SRSF7* with eGFP or mCherry, including phosphomimetics and chimeric constructs were generated by restriction and ligation cloning. All sequences were purchased as gBlocks DNA fragments (Integrated DNA Technologies) and amplified using specific primers (Additional file 1: Table S9) to add overhangs including *Nhe*I and *Kpn*I restriction sites. Plasmid containing either eGFP-N1 (Clontech) or mCherry-N1 (Clontech) and the respective PCR amplicons were digested using *Nhe*I-HF and *Kpn*I-HF (New England Biolabs) and ligated in a 1:6 ratio using T4 DNA Ligase (Promega).

#### Gibson Assembly cloning

Gibson assembly was used to generate mCherry and Luciferase reporter genes with different 3′UTR variants. Therefore, the backbones of the mCherry-N1 (Clontech) and Luciferase (Promega) plasmids were linearized and inserts were amplified by PCR using specific primers (Additional file 1: Table S9). Backbones and purified PCR inserts were mixed in a 1:3 ratio and ligated using a Gibson Assembly Mastermix (1.3x Isothermal Mastermix [100 mM Tris-HCl pH 7.5, 10 mM MgCl_2_, 200 nM dNTPs, 10 mM DTT, 1 mM NAD, 5% (w/v) PEG-8000], 0.1 U T5 exonuclease, 0.5 U Phusion DNA Polymerase, 0.1 U Taq DNA Ligase) at 50 °C for 15 min.

All ligation products were transformed into *E. coli* TOP10 cells and positive colonies were identified by Sanger sequencing (Eurofins). For the reporter gene assays 5 μg of each plasmid were transfected per 10 cm cell culture dish for 24 h using JetOPTIMUS Transfection Reagent (Polyplus) according to the manufacturer’s instructions. For CPSF6-myc (SinoBiological) and chimera expression experiments 1.5 μg of each plasmid were transfected per 6 cm cell culture dish for 24 h using Lipofectamine 2000 (ThermoFisher Scientific). Cells were starved with Opti-MEM® (ThermoFisher Scientific) for 4 h prior to transfection.

### Design, preparation, and transfection of esiRNAs

Suitable esiRNA target regions of approximately 400 nt were chosen using the DEQOR2 algorithm [74]. Template regions were amplified using primers that add a T7 promoter sequence (Additional file 1: Table S9) and in vitro transcribed using the HiScribe T7 High Yield RNA Synthesis Kit (NEB). Double-stranded RNAs were digested using RNase III (MPI-CBP, Dresden, Germany) at 37 °C for 2 h with agitation (1100 rpm). Digested esiRNAs were purified using Q Sepharose Fast Flow (Sigma-Aldrich), resuspended in TE buffer (pH 7.9), and stored at − 80 °C. Per 10 cm cell culture dish, 5 μg purified esiRNAs were transfected for 36 h (SRSF3&SRSF7) and 48 h (CPSF6) using JetPRIME Transfection Reagent (Polyplus) according to the manufacturer’s instructions. EsiRNAs against GFP (SRSF3 and SRSF7) or Luciferase (CPSF6) were used as controls.

### Reverse transcription (RT), rapid amplification of 3′cDNA ends (3′RACE), and qPCR

Total RNA was isolated using the TRIzol method (Invitrogen). Genomic DNA was removed by TURBO DNase (Invitrogen). RNA concentrations were measured using a NanoDrop2000 (ThermoFisher Scientific). For 3′RACE-PCR, 1 μg RNA was reverse transcribed into cDNA using an oligo (dT) primer or an anchored oligo (dT) primer including a platform sequence (Additional file 1: Table S9) and SuperScript III Reverse Transcriptase (Invitrogen). 3′RACE PCRs were done using gene-specific forward primers (Additional file 1: Table S9) and a reverse primer complementary to the platform sequence of the RT primer with 28 cycles. For qPCR, primers were chosen using Primer-BLAST (https://www.ncbi.nlm.nih.gov/tools/primer-blast/). qPCRs were performed using ORA qPCR Green ROX H Mix, 2x (HighQu) on a PicoReal 96 (ThermoFisher Scientific).

### RNA-seq and MACE-seq

For RNA-seq, 7 μg total RNA were subjected to poly(A) + selection and RNA-seq library preparation (Novogene). The libraries were sequenced on an Illumina HiSeq4000 instrument with either 75-bp single-end or 150-bp paired-end reads and 50–60 million reads per replicate.

MACE-seq libraries were prepared and sequenced at GenXPro GmbH (Frankfurt am Main, Germany) as described by [45, 46]. Total RNA was isolated as described before and 1 μg was submitted to GenXPro for downstream procedures. Briefly, first poly(A) + RNA was isolated from total RNA using the Invitrogen™ Dynabeads™ mRNA Purification Kit (ThermoFischer Scientific) followed by reverse transcription into cDNA using the Invitrogen™ SuperScript™ Double-Stranded cDNA Synthesis Kit (ThermoFischer Scientific) with an anchored biotinylated poly (dT) primer. Next, the cDNA was randomly fragmented to an average size of 250 bp by sonication using a Bioruptor (Diagenode). The biotinylated cDNA ends were captured by Invitrogen™ Dynabeads™ M-270 Streptavidin Beads (ThermoFischer Scientific) and ligated with T4 DNA Ligase 1 (NEB) to modified TrueQuant adapters (GenXPro). Libraries were amplified using KAPA HiFi Hot-Start Polymerase (KAPA Biosystems), followed by purification using Agencourt AMPure XP beads (Beckman Coulter). The libraries were sequenced on a Illumina HiSeq2000 platform yielding 75-bp single-end reads.

### RNA-seq data analysis

RNA-seq reads were obtained from Novogene and mapped against the mouse genome (version mm10) with GENCODE gene annotation (version M18) using STAR (v2.6.1d) [75] with the following parameters: --outSAMattributes All --outSAMtype BAM SortedByCoordinate --runThreadN 2 --outFilterMismatchNmax 2 --readFilesCommand zcat --quantMode GeneCounts. Mapped reads were counted with the summarizeOverlaps function of the GenomicAlignments (version v1.18.1) R/Bioconductor package using the “Union” mode and the exons of all genes in the GENCODE annotation (version M18) as features. The count tables were used for differential gene expression analysis with DESeq2 (version 1.22.2) [54]. Bam files were converted to bedGraph files using bedtools2 (v2.26.0) [76] and changes in 3′UTR length were analyzed with DaPars (version 0.9.1) using the GENCODE annotation [43]. For each reported gene with a change in 3′UTR-APA, the location of the pPAS and dPAS was extracted as well as the adjusted *P* value (false discovery rate, FDR) and the change in dPAS usage (ΔPDUI). Changes with an FDR ≤ 0.1 and a ΔPDUI ≥ 0.05 (longer 3′UTR) or ΔPDUI ≤ 0.05 (shorter 3′UTR) were considered significant.

### MACE-seq data analysis

The analysis of MACE-seq data was performed as described in [45]. In brief, low-quality regions were trimmed from both ends (Phred score < 16). PCR duplicates were removed based on unique molecular identifiers introduced during MACE-seq library preparation. Trailing A’s were then detected using a 5-nt sliding window, allowing one non-adenosine per window to account for sequencing errors. Reads with at least 10 trailing A’s (A_10_) were considered to arise from a poly(A) tail, which was subsequently trimmed off. Reads were then mapped against the mouse reference genome (mm10) using Novoalign (http://novocraft.com), keeping only uniquely mapped reads, controlled by “-r none” and without soft clipping (“-o FULLNW”).

To detect inadvertent internal priming events, homopolymeric A-stretches were determined by mapping A_10_ to the mouse reference genome (mm10) with Bowtie [77], allowing two errors (“-v 2, -r all”), transformed to BED format and merging overlapping intervals with BEDtools [76]. Putative poly(A) tail reads were then excluded if adjacent to a homopolymeric A-stretch. To identify clusters of reads for subsequent PAS definition, we used the combined 3′end coordinates of the poly(A) tail-trimmed reads from all three conditions (WT, *Srsf3* KD, *Srsf7* KD, two replicates each). These were assigned to clusters by iteratively extending the cluster to the next downstream 3′end coordinate if ≤ 25 nt away from the median 3′end coordinate of the cluster.

### Identification of PASs from MACE-seq data

In order to precisely assign PAS positions, we piled up all cleavage events (i.e. the positions 1 nt downstream of the 3′end coordinates of the poly(A) tail-trimmed reads) and resized the clusters to 15-nt windows centered on mode of cleavage events per nucleotide within the cluster. Cleavage events from each condition and replicate were then recounted into the 15-nt windows (CE_window_), and replicates averaged for each condition (CE_average_). Next, the percent usage (PU) was calculated for each 15-nt window by dividing CE_window_ by to all cleavage events in the complete gene * 100, and averaged between replicates for each condition (PU_average_). Next, a transcripts per million (TPM)-type metric was calculated for each 15-nt window, by dividing CE_window_ by window length (in kilobases; i.e., 0.015) by 1 million reads, averaged between replicates for each condition (TPM_average_), and then taken as maximum across all windows in a given gene (TPM_maximum_). For each 15-nt window, the maximum CE_window_, PU_average_, and TPM_maximum_ across the three conditions was used to apply the following filters: CE_window_ > 4 cleavage events AND PU_average_ > 5% AND TPM_maximum_ > 0.25. Finally, we annotated the positions of the detected PASs based on GENCODE gene annotation (version M18). In this context, 15-nt windows, whose center overlapped more than one gene or were more than 50 nt downstream of a gene were removed. This procedure yielded a total of 15,866 PASs mapping to 9148 genes, whereby the center of the 15-nt windows was considered as the actual cleavage site.

To assign the PASs to transcript regions, each PAS was checked for overlaps with 5′UTR, CDS, 3′UTR, or intronic regions retrieved from the GENCODE annotation. Except for PASs overlapping with 3′UTR and intronic regions, all PASs overlapping with more than one region (e.g., 5′UTR and CDS) were assigned to category “Other.” In addition, PASs located 1–50 nt downstream of a gene were assigned to the 3′UTR of that gene.

The spatial allocation of PASs as proximal PAS (pPAS), distal PAS (dPAS) or intermediate/other PAS (oPAS) was achieved by defining the most upstream PAS as pPAS for a given gene, the most downstream PAS as dPAS, and the remaining PASs located in between as oPAS. In the case of only a single PAS for a given gene, the PAS was defined as sPAS. This procedure yielded 5095 pPASs, 2589 oPASs, 4091 dPASs, and 5095 sPASs, whereby 2903 of the pPASs, 2169 of the oPASs, 3838 of the dPASs, and 4796 of the sPASs were located in annotated 3′UTRs.

### Motif analysis around PASs

To identify the associated CSEs (Additional file 1: Fig. S1J), the start positions of all CSE hexamers reported by [24] were determined in a window of 50 nt upstream of each PAS. A unique CSE was then assigned by applying the following hierarchy: AAUAAA > AUUAAA > other hexamers.

The integration of PAS coordinates from MACE-seq and APA changes (3′UTRs getting shorter or longer) reported by DaPars was done by matching PASs obtained by MACE-seq to PASs reported by DaPars. A DaPars PAS was considered as a match to a MACE-seq PAS if it was located at maximum 250 nt upstream or 50 nt downstream of the MACE-seq PAS (Additional file 1: Fig. S2A). In the case of multiple DaPars PASs matching the same MACE-seq PAS, the closer one was considered. Further, we restricted that the matched PASs were of the same type (pPAS-pPAS or dPAS-dPAS).

CNYC, GAY, and UGUA motif analyses were performed on the 13,706 PASs located in 3′UTRs. For each of these PASs, motif starts in a +/− 300-nt or +/− 500-nt window were identified, which served as basis for the subsequent analyses. Metaprofiles of motif distributions were generated by calculating the fraction of PASs with a motif start in a specific distance to the PAS, followed by a loess smoothing. In addition, for the metaprofiles of transcripts with shorter 3′UTRs in *Srsf3* or *Cpsf6* KD, matched metaprofiles of non-affected transcripts were generated. For this purpose, we randomly selected 100 times similar set sizes of non-affected transcripts and determined for each nucleotide around the PASs the average fraction of PASs with a motif start. The shown metaprofiles are loess smoothed, whereby the shaded area reflects the confidence interval. Regarding the fraction of PASs enclosed by tandem UGUAs (UGUA-PAS-UGUA) and those preceded (UGUA-UGUA-PAS) or followed (PAS-UGUA-UGUA) by tandem UGUAs were determined in windows of incrementing size (Win_size_; 1-nt steps). For PASs enclosed by tandem UGUAs, two windows were simultaneously incremented on either side of the PAS (Win_size_ 1–75 nt), whereby the first UGUA had to start in a range of [−Win_size_; − 4 nt] and the second UGUA in a range of [− 3 nt; Win_size_-3]. Regarding PASs preceded or followed by tandem UGUAs, the Win_size_ was in a range of 1–150 nt, whereby both UGUAs had to start in a range of [−Win_size_; − 4 nt] (preceded) or [1 nt; Win_size_-3] (followed). For each PAS, the minimum distance between two UGUAs is reported.

### iCLIP library preparation

Approximately 4 × 10^7^ P19 cells were irradiated once with 300 mJ/cm^2^ UV light at 254 nm (CL-1000, UVP) on ice. iCLIP was performed as described in [78] with minor modifications. Briefly, Dynabeads Protein G (Invitrogen) were coupled with goat anti-GFP antibody (provided by D. Drechsel, MPI-CBG, Dresden, Germany) and used for immunoprecipitation. Crosslinked RNA from the immunoprecipitated RNPs was digested into smaller fragments using RNase I (Invitrogen) and purified. RNA fragments were ligated to pre-adenylated DNA 3′adapters (Integrated DNA Technologies) and reverse-transcribed using barcoded RT primers by Invitrogen Superscript IV (ThermoFisher Scientific). cDNA fragments were size-selected and circularized by CircLigase II (Epicentre/Lucigene) before re-linearization using *Bam*HI HF (New England Biolabs). The final libraries were amplified using AccuPrime SuperMix I (ThermoFischer Scientific) and subjected to Illumina sequencing on a HighSeq2000 instrument with 75-bp single-end reads.

### Analysis of iCLIP data

Analysis of iCLIP sequencing data was done using the iCount package (http://icount.biolab.si). Briefly, adapters and barcodes were removed from all reads before mapping to the mouse mm9 genome assembly (Ensembl59 annotation) using the Bowtie aligner (version 0.12.7). To determine protein-RNA contact sites, all uniquely mapping reads were used, PCR duplicates were removed and crosslink events (X-links) were extracted (1st nucleotide of the read). To determine statistically significant X-links, a false discovery rate (FDR < 0.05) was calculated using normalized numbers of input X-links and randomized within co-transcribed regions [79–81]. To obtain comparable numbers of significant binding sites, replicates that correlated well were pooled according to their overall number of crosslink events.

For motif searching, a *z*-score analysis for enriched k-mers was performed as described previously [80]. Sequences surrounding significant X-links were extended in both directions by 30 nucleotides (windows: − 30 nt to − 5 nt, and 5 nt to 30 nt). All occurring k-mers within the evaluated interval were counted and weighted. Then, a control dataset was generated by randomly shuffling 100 times significant X-links within the same genes, and a *z*-score was calculated relative to the randomized genomic positions (https://github.com/tomazc/iCount). The top 25 k-mers were aligned to determine the in vivo binding consensus motif. Sequence logos were produced using WebLogo (http://weblogo.berkeley.edu/logo.cgi).

For metaprofiles of crosslink sites around pPASs, dPASs and sPASs, the positions with crosslink events in a window of − 400 nt to 100 nt around the PAS were extracted. Afterwards, for each PAS type and protein (SRSF3, SRSF7, CPSF5, and FIP1), two normalization steps were conducted. In the first normalization step, the summed crosslink sites were normalized to the number of PASs in this category to make signals around pPASs, dPASs, and sPASs comparable. In the second step, we normalized for the total number positions with crosslink sites in the iCLIP library to make signals of different iCLIP libraries comparable. Binding signals were smoothed with the loess function. Signal differences between two iCLIP libraries (e.g., SRSF3 and SRSF7) were determined nucleotide-wise by two-proportions *z*-tests, followed by a correction for multiple hypothesis testing using the Benjamini-Hochberg procedure. Positions with FDR ≤ 0.01 were considered as significant. To account for different sequencing depths, the proportions were normalized via a scaling factor before the test. The scaling factor was calculated as number of positions with crosslink sites in the first iCLIP library divided by the number of positions with crosslink sites in the second iCLIP library.

Metaprofiles around PASs with reduced or increased usage in KDs or during differentiation were compared to metaprofiles of matched background sets of PASs with unchanged usage. This procedure is exemplified for the iCLIP signal of SRSF3 around 197 pPASs with increased usage upon *Srsf3* KD (Fig. 2b, upper panel). For the background set, we randomly selected 100 times 197 pPASs with unchanged usage upon *Srsf3* KD and determined for each of the 100 sets the iCLIP signal of SRSF3 as described above. Based on the resulting 100 profiles, we calculated for each position the mean and standard deviation and used the two measures to calculate a *z*-score for each position in the SRSF3 binding signal of the 197 pPAS with increased usage. *z*-scores were then transferred into *P* values and corrected for multiple hypothesis testing using the Benjamini-Hochberg procedure. *z*-scores with an FDR ≤ 0.01% are shown and reflect a significant difference in iCLIP signal between pPAS with increased and unchanged usage upon *Srsf3* KD.

### Immunofluorescence microscopy and image acquisition

Adherent cells were fixed with 4% paraformaldehyde (PFA; Sigma-Aldrich) for 20 min at RT, washed and permeabilized in 5% BSA, 0.1% Triton in PBS for 30 min at RT. DNA was counterstained with Hoechst 34580 (Sigma) in TBST (20 mM Tris-HCl, 150 mM NaCl, 0.1% Tween 20 pH 7.5; 1:4000) at RT for 30 min. After washing, the cells were dried and mounted on slides using ProLong™ Diamond Antifade Mountant (Thermo Fisher Scientific) and stored at 4 °C until imaging. Images were acquired using a confocal laser-scanning microscope (LSM780; ZEISS) with a Plan-Apochromat × 63 1.4 numerical aperture oil differential interference contrast objective equipped with two photomultiplier tubes and a gallium arsenite phosphate (GaAsPPMT) detector system. Fluorescence signal was detected with an Argon laser (GFP, 488 nm). Fiji was used to process all acquired images [82]. Pictures were cropped with the Image crop function and scale bars were added.

## Supplementary Information

**Additional file 1.**

**Additional file 2.**

**Additional file 3.**

**Additional file 4.**

**Additional file 5.**

**Additional file 6.**

**Additional file 7.**

**Additional file 8.**

**Additional file 9.**

**Additional file 10.**

**Additional file 11.**

## Data Availability

All iCLIP, RNA-seq, and MACE-seq data generated and/or analyzed during the current study have been submitted to Gene Expression Omnibus (GEO) under the SuperSeries accession GSE151724. The mass spectrometry proteomics data have been deposited to the ProteomeXchange Consortium via the PRIDE partner repository with the dataset identifier PXD018090. The computational code for the motif analyses and the RBP binding maps is available via the GitHub repository [83].
