# Supplementary material for: SRSF3 and SRSF7 modulate 3′UTR length through suppression or activation of proximal polyadenylation sites and regulation of CFIm levels
Source: Genome Biol. 2021 Mar 11;22:82. doi: 10.1186/s13059-021-02298-y (PMC7948361; doi:10.1186/s13059-021-02298-y)
Supplement: Supplementary file 1 — Additional file 1. [file 13059_2021_2298_MOESM1_ESM.pdf]

SRSF3 and SRSF7 modulate 3'UTR length through suppression or activation of proximal polyadenylation sites and regulation of CFIm levels

Oliver Daniel Schwich[1,2,§], Nicole Blümel[1,§], Mario Keller[2,§], Marius Wegener[1,2], Samarth Thonta Setty[2], Melinda Elaine Brunstein[3], Ina Poser[4], Igor Ruiz De Los Mozos[5], Beatrix Suess[6], Christian Münch[3], François McNicoll[1], Kathi Zarnack[2,#], Michaela Müller-McNicoll[1,#]

[1]Institute of Cell Biology & Neuroscience, Goethe University Frankfurt, Max-von-Laue-Str. 13, 60438 Frankfurt, Germany; [2]Buchmann Institute for Molecular Life Sciences, Goethe University Frankfurt, Max-von-Laue-Str. 15, 60438 Frankfurt, Germany; [3]Institute of Biochemistry II, Medical School, Goethe University Frankfurt, Sandhofstr. 2-4, 60528 Frankfurt am Main, Germany; [4]Max Planck Institute of Molecular Cell Biology and Genetics, Pfotenhauerstr. 108, 01307 Dresden, Germany; [5]The Francis Crick Institute, 1 Midland Road, London NW1 1AT, United Kingdom; [6]Technical University Darmstadt, Schnittspahnstr. 10, 64287 Darmstadt, Germany

[§] These authors contributed equally; [#] Co-corresponding authors

SUPPLEMENTARY MATERIAL

Content:

Supplementary Figures 2

Supplementary Tables 20

Additional Files 24

Supplementary Figures

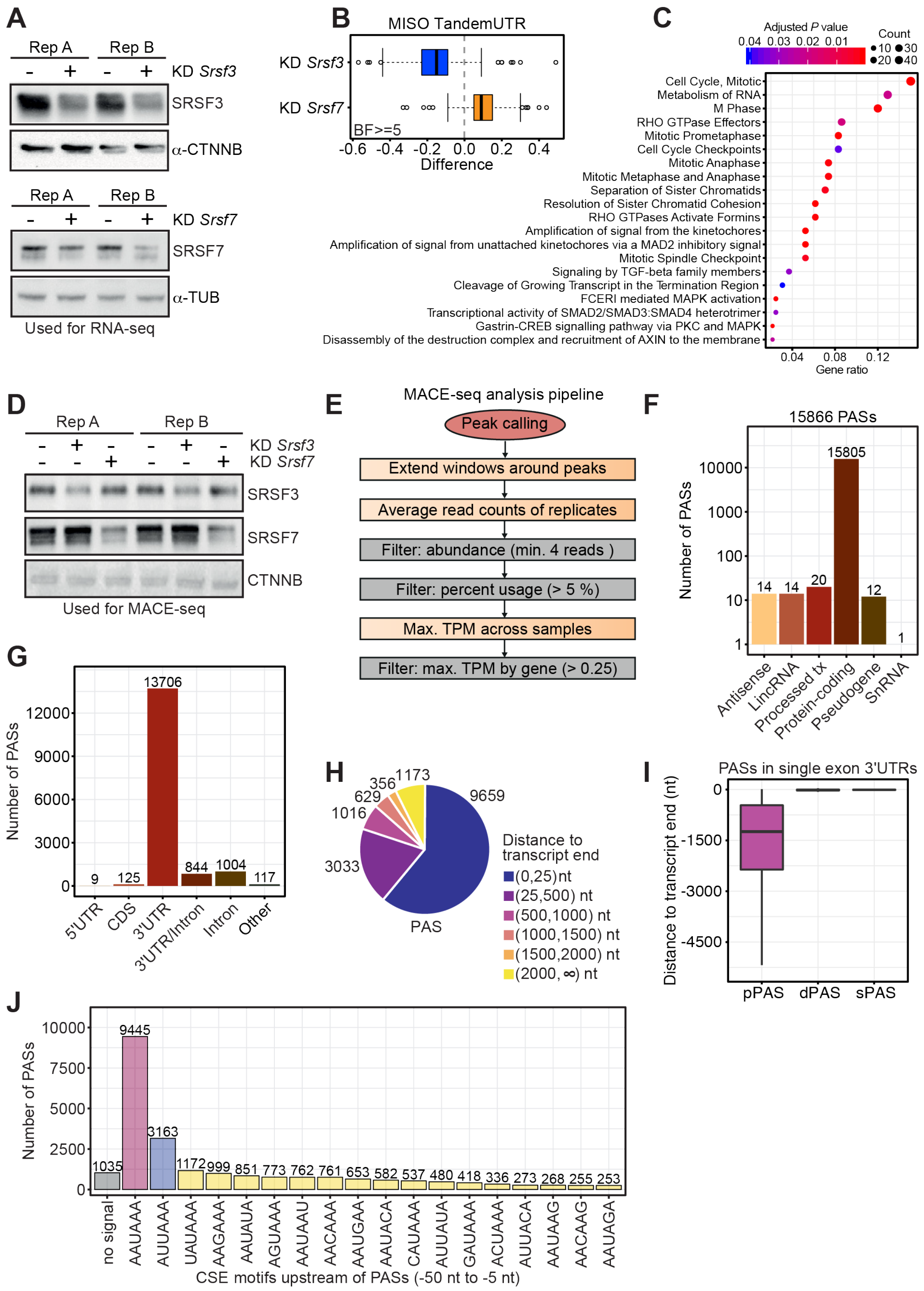

Fig. S1: SRSF3 and SRSF7 exert opposite effects on 3′UTR length. (A) Western blot after knockdown (KD) of *Srsf3* (top) or *Srsf7* (bottom) in P19 wt cells used for RNA-seq. An antibody against α-tubulin (α-TUB) or β-catenin (CTNNB) was used as loading control. **(B)** Analysis of changes in the lengths of annotated tandem UTRs using MISO. **(C)** Pathway analysis of transcripts with shortened 3′UTRs after KD of *Srsf3*. **(D)** Western blot after KD of *Srsf3* (top) or *Srsf7* (bottom) in P19 wt cells used for MACE-seq. An antibody against β-catenin (CTNNB) was used as loading control. **(E)** Scheme of the MACE-seq analysis pipeline. TPM, transcripts per million. **(F)** Number of used poly(A) sites (PASs) identified in different RNA biotypes. lincRNA, long intergenic noncoding RNA; snRNA, small nuclear RNA. **(G)** Number of used PASs per transcript region. PASs overlapping with both 3′UTR and intronic regions in distinct isoforms were collected in a separate category 3′UTR/Intron. **(H)** Distance of used PASs to annotated transcript ends. Distances were binned as indicated on the right. **(I)** Distances of proximal PASs (pPASs), distal PASs (dPASs), and single PASs (sPASs) to annotated transcript ends. **(J)** Number of used PASs with known central sequence elements (CSEs) and no signals.

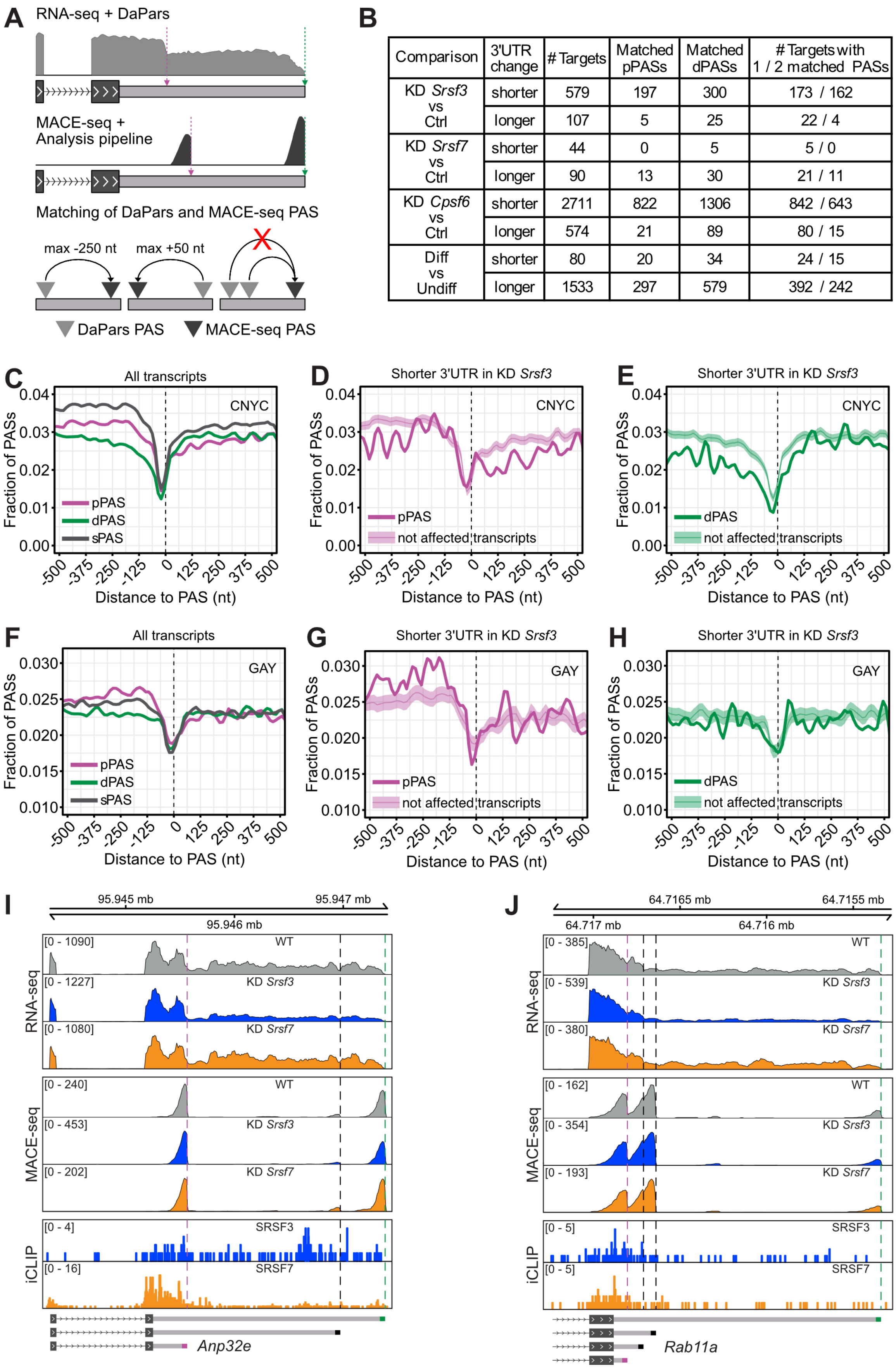

Comparison	3'UTR change	# Targets	Matched pPASs	Matched dPASs	# Targets with 1 / 2 matched PASs
KD *Srsf3* vs Ctrl	shorter	579	197	300	173 / 162
	longer	107	5	25	22 / 4
KD *Srsf7* vs Ctrl	shorter	44	0	5	5 / 0
	longer	90	13	30	21 / 11
KD *Cpsf6* vs Ctrl	shorter	2711	822	1306	842 / 643
	longer	574	21	89	80 / 15
Diff vs Undiff	shorter	80	20	34	24 / 15
	longer	1533	297	579	392 / 242

Fig. S2: Extraction of PAS coordinates. (A) Workflow for the matching of DaPars and MACE-seq data. DaPars identifies the approximate position of pPASs by searching for significant changes in the RNA-seq coverage and infers the position of dPASs from the provided annotation (upper panel). Our analysis pipeline identifies the exact position of pPASs and dPASs by the identification of clusters of poly(A) tail-trimmed reads from MACE-seq (middle panel). Information about PAS usage changes provided by DaPars and the exact PAS positions of our analysis pipeline were combined by matching DaPars PAS coordinates to MACE-seq PAS coordinates (lower panel). The maximum allowed distance of a DaPars PAS to a MACE-seq PAS was 250 nt upstream and 50 nt downstream. In the case of two DaPars PASs matching the same MACE-seq PAS, the closer one was assigned. **(B)** Statistics for matched PASs in different datasets used in this study. For each DaPars comparison (column 1) the number of targets (column 3) with a certain change in 3′UTR length (column 2) is shown. The number of targets whose pPAS and dPAS could be matched to a MACE-seq PAS are indicated in columns 4 and 5. The last column indicates the number of targets with one (pPAS or dPAS) or two matched PASs (pPAS and dPAS). **(C)** Enrichment of CNYC motifs around sPASs, pPASs and dPASs used in P19 cells. **(D&E)** CNYC motif enrichment around the pPAS **(D)** and dPAS **(E)** in SRSF3-regulated targets and non-affected transcripts. **(F)** Enrichment of GAY motifs around sPASs, pPASs and dPASs used in P19 cells. **(G&H)** GAY motif enrichment around the pPAS (G) and dPAS (H) in SRSF3-regulated targets and non-affected transcripts. **(I&J)** Browser shots of the *Anp32e* (I) and *Rab11a* (J) 3′UTRs: Top: RNA-seq read coverage in control conditions (WT) and after KD of *Srsf3* or *Srsf7*. Middle: Coverage of MACE-seq reads mapping to the pPAS and dPAS. Bottom: iCLIP crosslink events of SRSF3 and SRSF7.

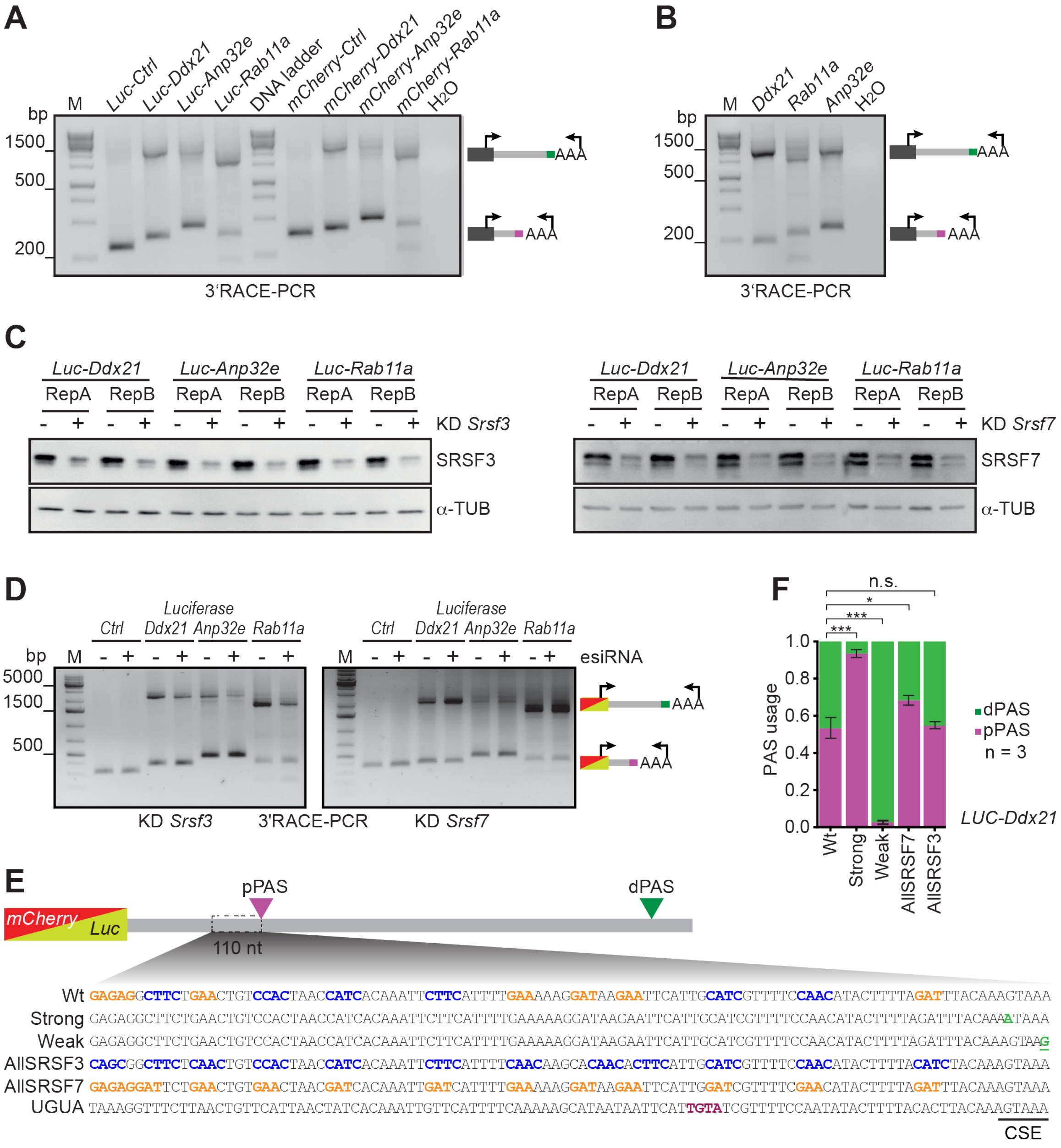
A
bp
M
Luc-Ctrl
Luc-Ddx21
Luc-Anp32e
Luc-Rab11a
DNA ladder
mCherry-Ctrl
mCherry-Ddx21
mCherry-Anp32e
mCherry-Rab11a
H2O
1500
500
200
AAA
AAA
3'RACE-PCR
B
bp
M
Ddx21
Rab11a
Anp32e
H2O
1500
500
200
AAA
AAA
3'RACE-PCR
C
Luc-Ddx21
Luc-Anp32e
Luc-Rab11a
RepA RepB RepA RepB RepA RepB
- + - + - + - + - + - +
KD Srsf3
SRSF3
α-TUB
Luc-Ddx21
Luc-Anp32e
Luc-Rab11a
RepA RepB RepA RepB RepA RepB
- + - + - + - + - + - +
KD Srsf7
SRSF7
α-TUB
D
Luciferase
Ctrl Ddx21 Anp32e Rab11a
bp
M
- + - + - + - +
5000
1500
500
KD Srsf3
3'RACE-PCR
Luciferase
Ctrl Ddx21 Anp32e Rab11a
M
- + - + - + - +
esiRNA
AAA
AAA
KD Srsf7
F
n.s.
*
***
***
1.0
0.8
0.6
0.4
0.2
0.0
PAS usage
dPAS
pPAS
n = 3
LUC-Ddx21
Wt
Strong
Weak
AllSRSF7
AllSRSF3
E
pPAS
dPAS
mCherry
Luc
110 nt
Wt GAGAGGCTTCTGAACTGTCCACTAACCATCACAAATTCTTCATTTTGAAAAAGGATAAGAATTCATTGCATCGTTTTCCAACATACTTTTAGATTTACAAAGTAAA
Strong GAGAGGCTTCTGAACTGTCCACTAACCATCACAAATTCTTCATTTTGAAAAAGGATAAGAATTCATTGCATCGTTTTCCAACATACTTTTAGATTTACAAAATAAA
Weak GAGAGGCTTCTGAACTGTCCACTAACCATCACAAATTCTTCATTTTGAAAAAGGATAAGAATTCATTGCATCGTTTTCCAACATACTTTTAGATTTACAAAGTAAG
AllSRSF3 CAGCGGCTTCTCAACTGTCCACTAACCATCACAAATTCTTCATTTTCAACAAGCACAACACTTCATTGCATCGTTTTCCAACATACTTTTACATCTACAAAGTAAA
AllSRSF7 GAGAGGATTCTGAACTGTGAACTAACGATCACAAATTGATCATTTTGAAAAAGGATAAGAATTCATTGGATCGTTTTCGAACATACTTTTAGATTTACAAAGTAAA
UGUA TAAAGGTTTCTTAACTGTTCATTAACTATCACAAATTGTTCATTTTCAAAAAGCATAATAATTCATTGTATCGTTTTCCAATATACTTTTACACTTACAAAGTAAA
CSE

Fig. S3: SRSF3 and SRSF7 bind at pPASs and modulate their usage in a splicing-independent manner. (A) 3′RACE-PCR of *Luciferase (Luc)* and *mCherry* reporter transcripts in P19 wt cells. **(B)** 3′RACE-PCR of endogenous *Ddx21*, *Anp32e* and *Rab11a* 3′UTR-APA isoforms. **(C)** Western blot of P19 cells transfected with *Luc* reporter constructs after KD of *Srsf3* (left) or *Srsf7* (right). An antibody against α-tubulin (α-TUB) was used as loading control. **(D)** 3′RACE-PCR of *Luc* reporter transcripts after KD of *Srsf3* or *Srsf7*. **(E)** Scheme of the *Luc-Ddx21* or *mCherry-Ddx21* reporter mutants. The pPAS was mutated either to the canonical CSE AAUAAA (strong pPAS) or to the inactive motif AGTAAG (weak pPAS) by point mutations (bold/underlined). All SRSF7-binding motifs (orange) in a window of 110 nt upstream of the pPAS were converted to SRSF3-binding motifs (AllSRSF3, blue). All SRSF3-binding motifs (blue) were converted to SRSF7-binding motifs (AllSRSF7, orange). All SRSF3- and SRSF7-binding motifs were removed and an UGUA motif was inserted (UGUA, purple). **(F)** Quantification of 3′RACE PCRs of transiently expressed *Luc-Ddx21* reporters with mutated pPAS regions (n = 3), Student's t-test, * P value < 0.05, *** P value < 0.005).

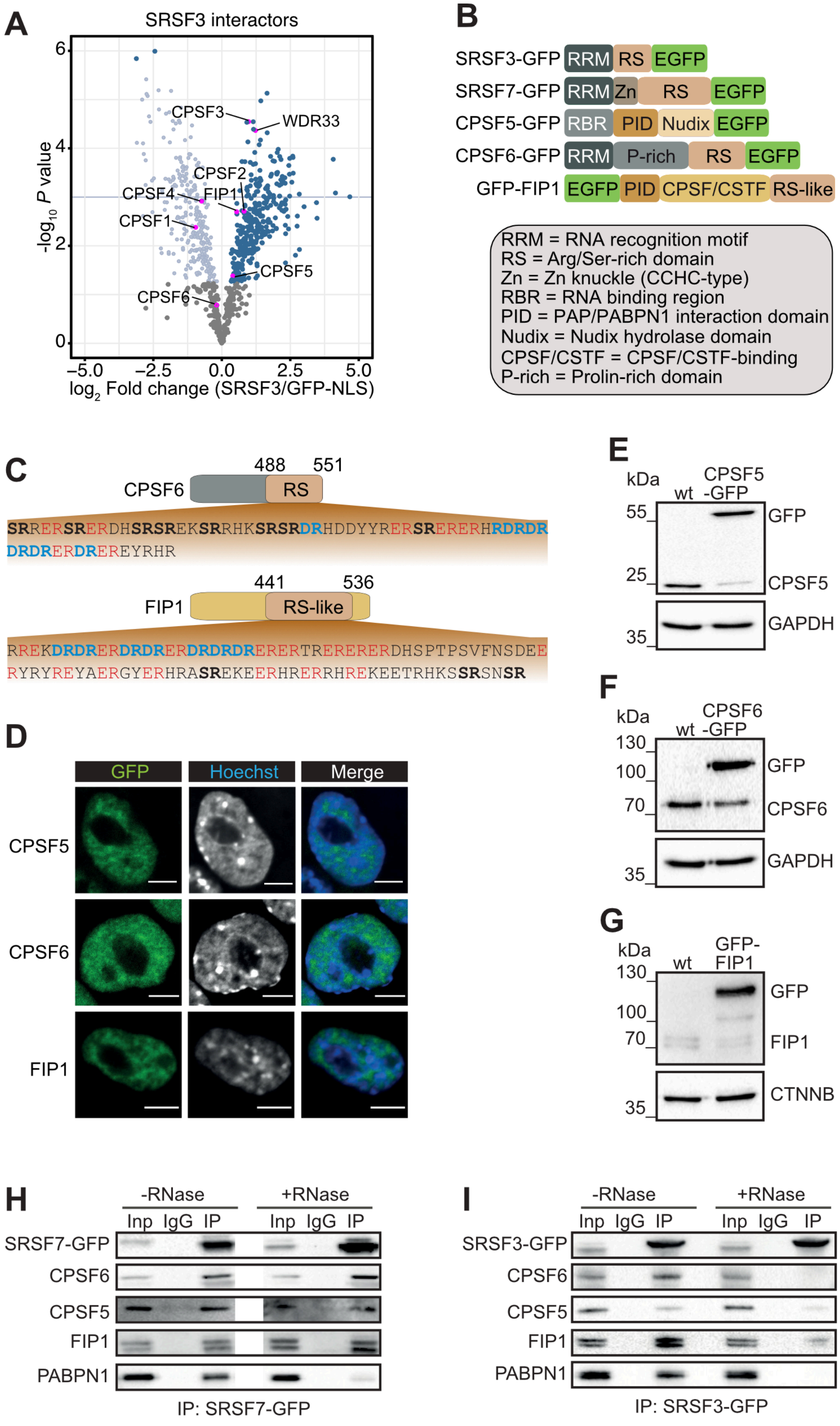
A
SRSF3 interactors
CPSF3
WDR33
CPSF2
CPSF4
FIP1
CPSF1
CPSF5
CPSF6
-log10 P value
log2 Fold change (SRSF3/GFP-NLS)
B
SRSF3-GFP RRM RS EGFP
SRSF7-GFP RRM Zn RS EGFP
CPSF5-GFP RBR PID Nudix EGFP
CPSF6-GFP RRM P-rich RS EGFP
GFP-FIP1 EGFP PID CPSF/CSTF RS-like
RRM = RNA recognition motif
RS = Arg/Ser-rich domain
Zn = Zn knuckle (CCHC-type)
RBR = RNA binding region
PID = PAP/PABPN1 interaction domain
Nudix = Nudix hydrolase domain
CPSF/CSTF = CPSF/CSTF-binding
P-rich = Prolin-rich domain
C
CPSF6 488 551 RS
SRRERSRERDHSRSREKSRRHKSRSRDRHDDYYRERSRERERHRDRDR
DRDRERDREREYRHR
FIP1 441 536 RS-like
RREKDRDRERDRDRERDRDRDREREREERTREREREERDHSPTPSVFNSDEE
RYRYREYAERGYERHRASREKEERHRERRHREKEETRHKSSRSNSR
D
GFP Hoechst Merge
CPSF5
CPSF6
FIP1
E
kDa wt CPSF5-GFP
55 GFP
25 CPSF5
35 GAPDH
F
kDa wt CPSF6-GFP
130 100 GFP
70 CPSF6
35 GAPDH
G
kDa wt GFP-FIP1
130 GFP
100 70 FIP1
35 CTNNB
H
-RNase +RNase
Inp IgG IP Inp IgG IP
SRSF7-GFP
CPSF6
CPSF5
FIP1
PABPN1
IP: SRSF7-GFP
I
-RNase +RNase
Inp IgG IP Inp IgG IP
SRSF3-GFP
CPSF6
CPSF5
FIP1
PABPN1
IP: SRSF3-GFP

Fig. S4: SRSF7 directly interacts with CPA factors. (A) Volcano plot of 832 proteins quantified from SRSF3-GFP-containing ribonucleoprotein particles (RNPs) after normalization to GFP (n = 3). 331 proteins were significantly enriched (*P* value ≤ 0.05, Benjamini-Hochberg correction; $\log_2$-transformed fold change [Log2FC] ≥ 1) and are highlighted in dark blue. CPA factors are labeled. **(B)** Domain architecture of GFP-tagged SRSF3, SRSF7, CPSF5, CPSF6 and FIP1 stably expressed in P19 cells. **(C)** Amino acid sequence of the RS domains in CPSF6 and FIP1. SR repeats are indicated in bold, ER and DR repeats in blue and red, respectively. **(D)** Fluorescence microscopy of P19 cells stably expressing GFP-tagged CPSF5, CPSF6 or FIP1 proteins. Hoechst staining was used to stain the nucleus. **(E-G)** Western blots to detect endogenous and GFP-tagged CPSF5, CPSF6 and FIP1 using protein-specific antibodies. GAPDH and CTNNB were used as loading controls. **(H&I)** Co-immunoprecipitations (Co-IPs) using GFP-tagged SRSF7 and SRSF3. Proteins were pulled-down by α-GFP antibodies and probed for interactions with CPSF5, FIP1 and CPSF6 using specific antibodies. Lysates were treated with (+RNase) or without RNase A (-RNase) prior to IPs. The nuclear polyA-binding protein PABPN1 served as a control for successful RNA degradation.

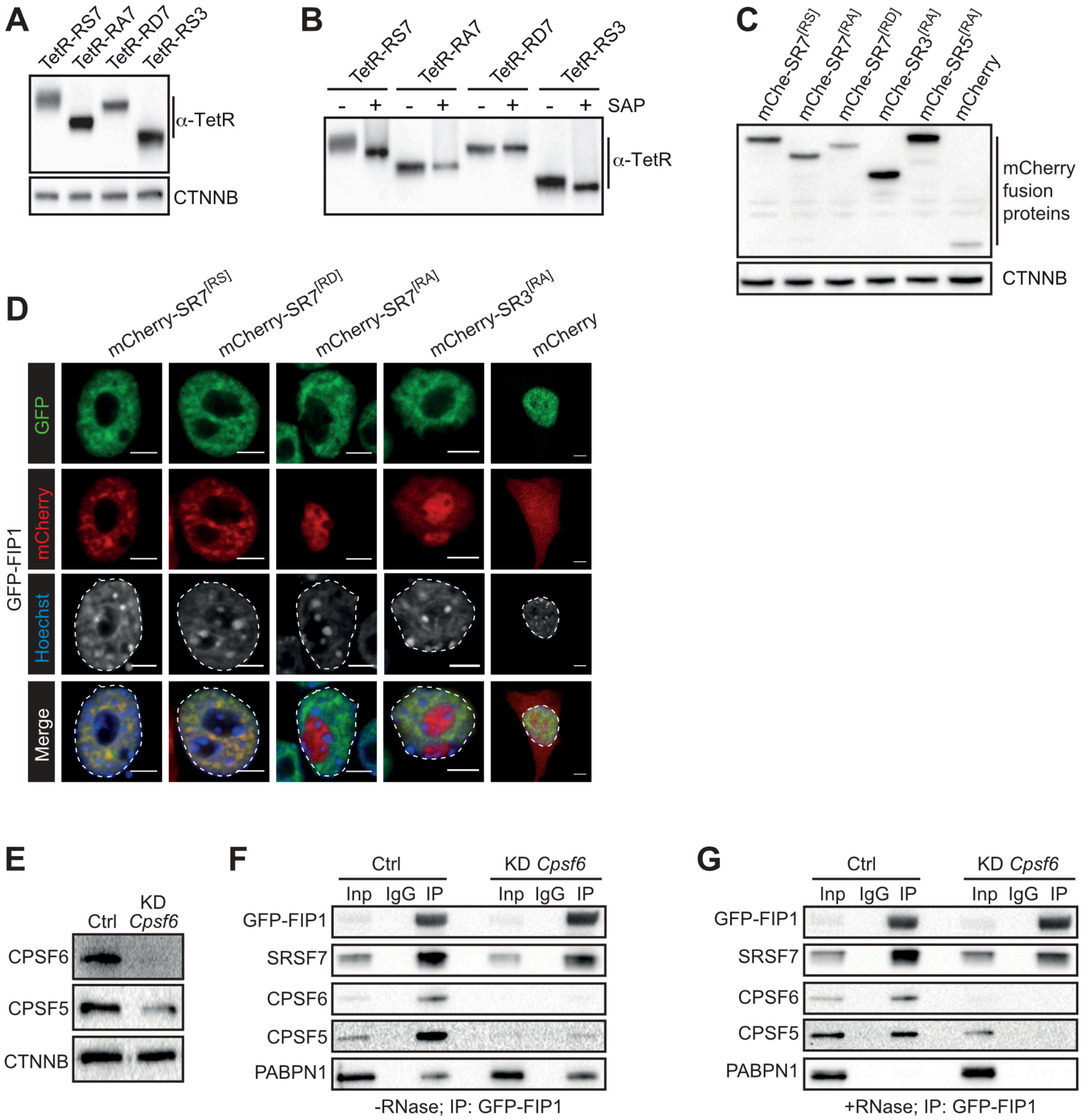
A
TetR-RS7
TetR-RA7
TetR-RD7
TetR-RS3
α-TetR
CTNNB
B
TetR-RS7
TetR-RA7
TetR-RD7
TetR-RS3
- + - + - + - + SAP
α-TetR
C
mChe-SR7[RS]
mChe-SR7[RA]
mChe-SR7[RD]
mChe-SR3[RA]
mChe-SR5[RA]
mCherry
mCherry fusion proteins
CTNNB
D
mCherry-SR7[RS]
mCherry-SR7[RD]
mCherry-SR7[RA]
mCherry-SR3[RA]
mCherry
GFP-FIP1
GFP
mCherry
Hoechst
Merge
E
Ctrl
KD Cpsf6
CPSF6
CPSF5
CTNNB
F
Ctrl
KD Cpsf6
Inp IgG IP Inp IgG IP
GFP-FIP1
SRSF7
CPSF6
CPSF5
PABPN1
-RNase; IP: GFP-FIP1
G
Ctrl
KD Cpsf6
Inp IgG IP Inp IgG IP
GFP-FIP1
SRSF7
CPSF6
CPSF5
PABPN1
+RNase; IP: GFP-FIP1

Fig. S5: SRSF7 interacts with FIP1 via its hypo-phosphorylated RS domain. (A) Western blot to confirm TetR-RS-domain fusion protein expression in P19 wt cells using α-TetR antibodies. CTNNB was used as loading control. **(B)** Western blot of TetR-RS-domain fusion proteins after shrimp alkaline phosphatase (SAP) treatment using α-TetR antibodies. Phosphorylated proteins show a reduction in molecular weight upon SAP treatment. **(C)** Western blot to validate expression of mCherry-tagged phosphomimetics using an α-mCherry antibody. CTNNB was used as loading control. **(D)** Confocal fluorescence microscopy to assess co-localization of mCherry-tagged phosphomimetics of SRSF7 and SRSF3 with GFP-FIP1. Hoechst was used to stain the nucleus. Nuclei are labeled by dashed lines. An empty mCherry vector was transfected as control. Scale bars = 5 μm. **(E)** Western blot to validate loss of CPSF6 and co-depletion of CPSF5 the upon KD of *Cpsf6* using specific antibodies. CTNNB was used as loading control. **(F&G)** Co-immunoprecipitations (Co-IPs) of GFP-FIP1 after KD of *Cpsf6*. GFP-FIP1 was pulled-down by α-GFP antibodies and probed for interaction with SRSF7, SRSF3, CPSF6 and CPSF5 using specific antibodies. Samples were treated without (-RNase) (I) or with (+RNase) (G) RNase A prior to IPs. PABPN1 served as a control for RNA degradation.

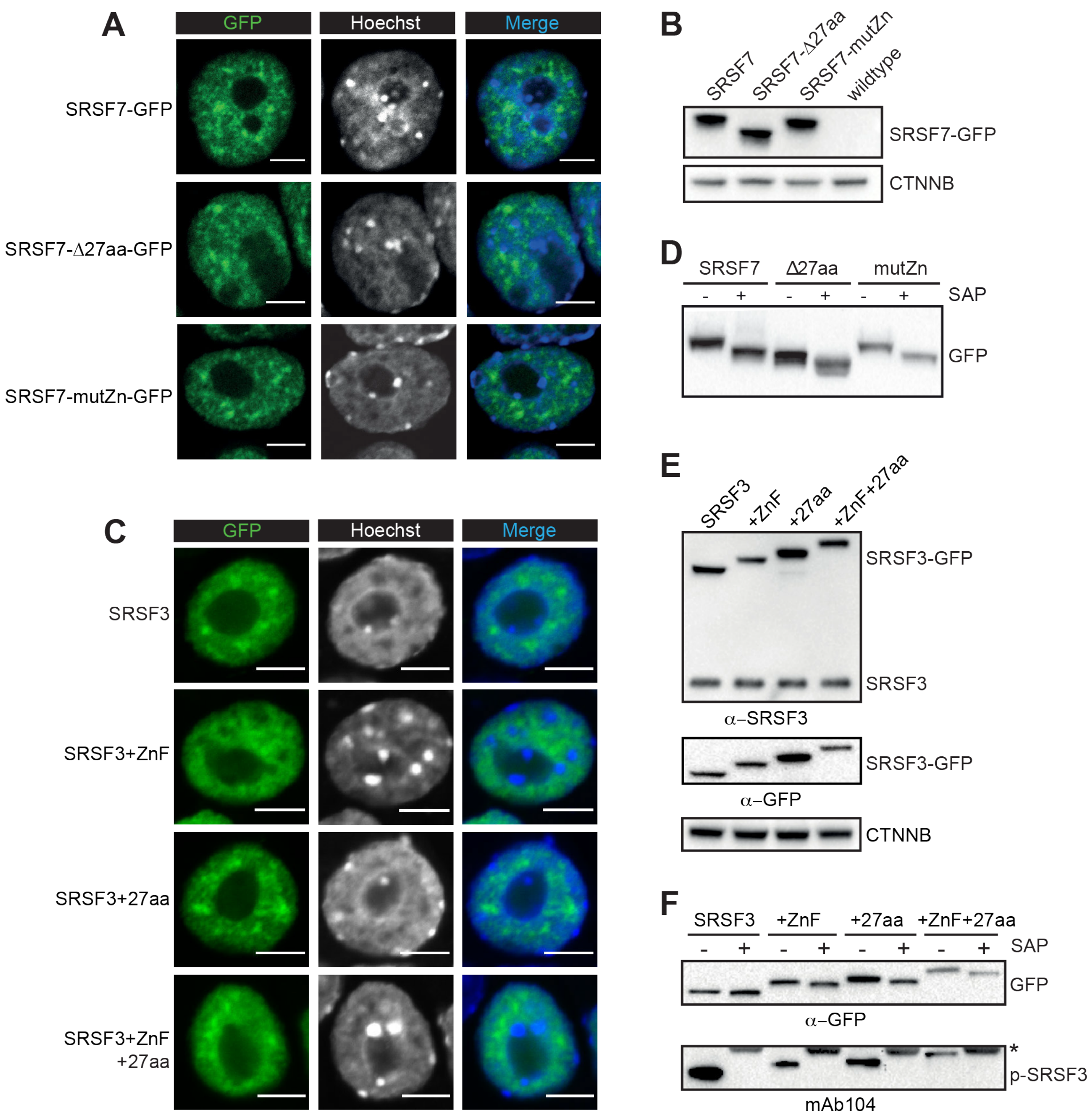
A
GFP
Hoechst
Merge
SRSF7-GFP
SRSF7-Δ27aa-GFP
SRSF7-mutZn-GFP
B
SRSF7
SRSF7-Δ27aa
SRSF7-mutZn
wildtype
SRSF7-GFP
CTNNB
C
SRSF3
SRSF3+ZnF
SRSF3+27aa
SRSF3+ZnF +27aa
D
SRSF7
Δ27aa
mutZn
SAP
GFP
E
+ZnF
+27aa
+ZnF+27aa
SRSF3-GFP
α–SRSF3
α–GFP
F
GFP
mAb104
p-SRSF3
*

Fig. S6: The hydrophobic stretch in the SRSF7 RS domain contributes to its interaction with CPA factors. (A) Confocal fluorescence microscopy to assay nuclear localization of GFP-tagged SRSF7 mutants stably expressed in P19 cells. Hoechst was used to stain the nucleus. Scale bars = 5 μm. **(B)** Western blot to confirm similar expression levels of GFP-tagged mutants of SRSF7. CTNNB was used as loading control. **(C)** Confocal fluorescence microscopy to assay nuclear localization of GFP-tagged SRSF3 chimeric proteins transiently expressed in P19 cells. Hoechst was used to stain the nuclei. Scale bars = 5 μm. **(D)** Western blot to validate the phosphorylation level of SRSF7 mutants using shrimp alkaline phosphatase (SAP) treatment and α-GFP antibodies. **(E)** Western blot probed with α-GFP and α-SRSF3 to compare expression levels of chimeric SRSF3 proteins to endogenous SRSF3. **(F)** Western blot of SRSF3 chimeric proteins after SAP treatment to verify RS domain phosphorylation. The blots were probed with α-GFP antibodies (top) and mAb104 antibodies (bottom), which detect only phosphorylated SR proteins. Unspecific detection of the SAP enzyme is indicated with an asterisk.

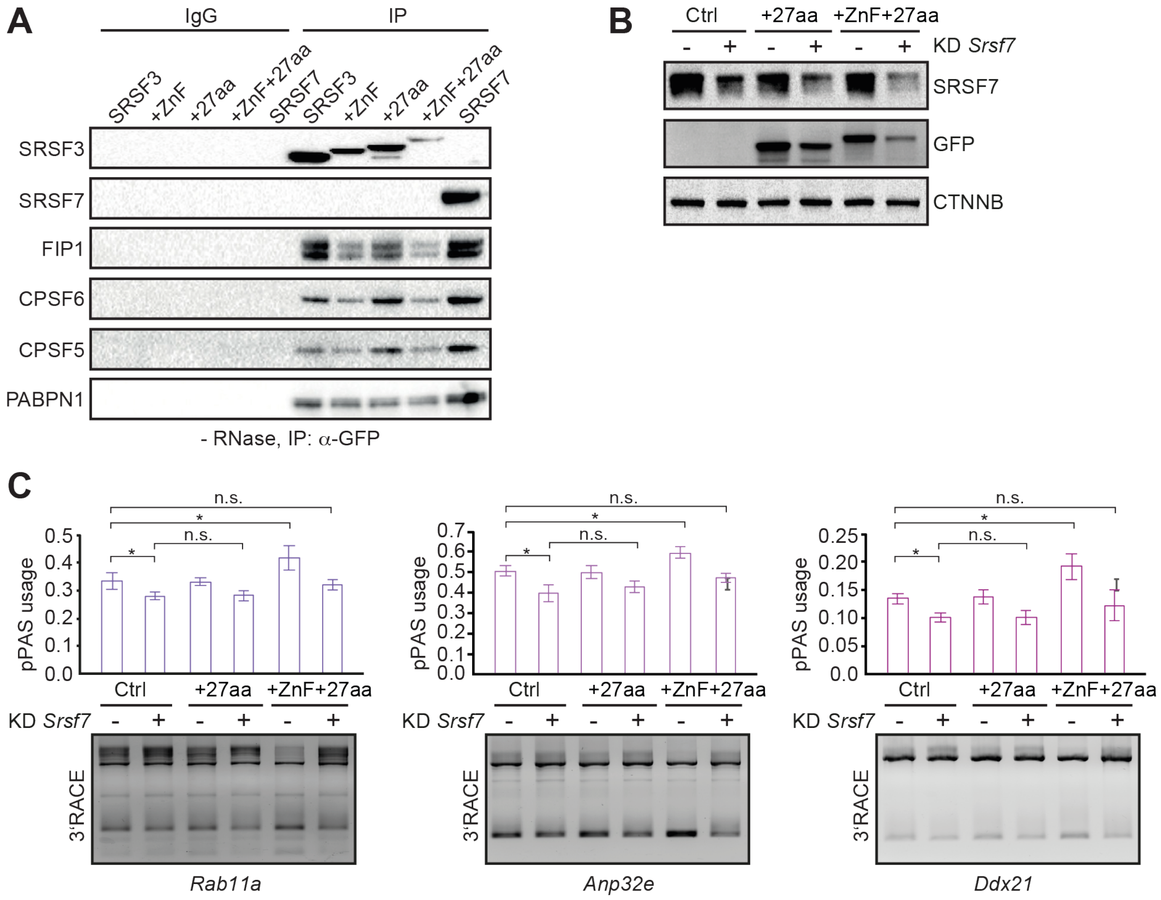

Fig. S7: The Zn knuckle and the 27aa hydrophobic stretch in the SRSF7 RS domain are both required to enhance pPAS usage. (A) Co-IPs using GFP-tagged SRSF3 chimeric proteins without RNase A treatment (-RNase): Proteins were pulled-down by α-GFP antibodies and probed for CPA factors using specific antibodies. PABPN1 served as a control for RNA integrity. **(B)** Western blot to confirm expression of SRSF3-27aa and SRSF3-27aa+ZnF chimeric proteins and SRSF7 depletion. The blots were probed with α-SRSF7 antibodies (top), α-GFP antibodies (middle) and CTTNB (bottom) as loading control. **(C)** Quantification of 3'RACE-PCRs (n = 3) of *Rab11a*, *Anp32e* and *Ddx21* transcripts in Ctrl and SRSF7-depleted P19 cells transiently overexpressing SRSF3-27aa and SRSF3-27aa+ZnF chimeric proteins. Data are represented as mean ± standard deviation of mean. Student's t-test, * *P* value < 0.05.

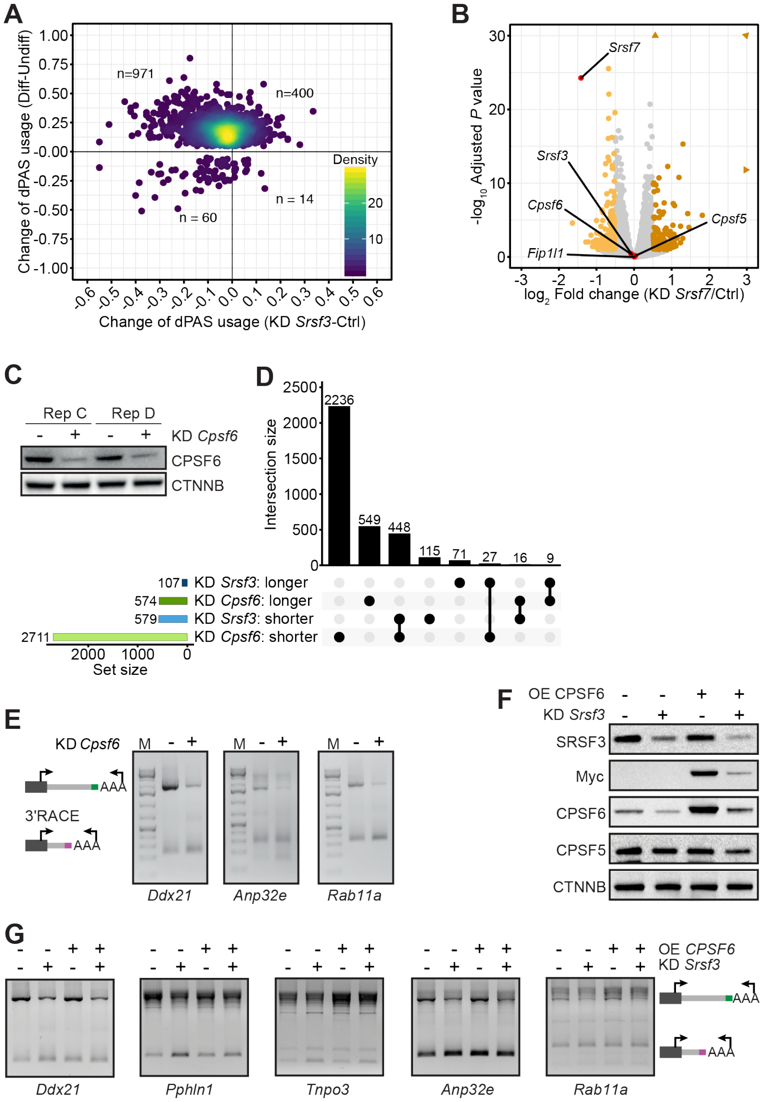
A
n=971
n=400
n = 14
n = 60
Density
Change of dPAS usage (Diff-Undiff)
Change of dPAS usage (KD Srsf3-Ctrl)
B
Srsf7
Srsf3
Cpsf6
Cpsf5
Fip1l1
-log10 Adjusted P value
log2 Fold change (KD Srsf7/Ctrl)
C
Rep C
Rep D
KD Cpsf6
CPSF6
CTNNB
D
Intersection size
2236
549
448
115
71
27
16
9
107 KD Srsf3: longer
574 KD Cpsf6: longer
579 KD Srsf3: shorter
2711 KD Cpsf6: shorter
Set size
E
KD Cpsf6
3'RACE
AAA
Ddx21
Anp32e
Rab11a
F
OE CPSF6
KD Srsf3
SRSF3
Myc
CPSF6
CPSF5
CTNNB
G
OE CPSF6
KD Srsf3
Ddx21
Pphln1
Tnpo3
Anp32e
Rab11a

Fig. S8: SRSF3 promotes dPAS usage by maintaining high levels of CFIm. (A) Scatter plot comparing dPAS usage (distal polyA site usage index, PDUI) upon differentiation and KD of *Srsf3*. **(B)** Volcano plot of differential gene expression upon KD of *Srsf7* analyzed by DESeq2. Significant genes (adjusted P value ≤ 0.1) are highlighted in orange. Genes of CPA factors, *Srsf3* and *Srsf7* are indicated. **(C)** Western blot using specific antibodies to confirm *Cpsf6* KD in P19 wt cells used for RNA-seq. CTNNB was used as loading control. **(D)** UpSet plot of genes with significant changes in 3'UTR-APA after KD of *Srsf3* and *Cpsf6*. Numbers of target genes and direction of regulation are given as barchart below. **(E)** 3'RACE-PCR of endogenous *Ddx21*, *Anp32e* and *Rab11a* 3'UTR-APA isoforms after KD of *Cpsf6* using gene-specific forward primers. **(F)** Western blot to confirm expression of CPSF6-myc and SRSF3 depletion. The blots were probed with α-SRSF3, α-myc, α-CPSF6 and α-CPSF5 antibodies. CTTNB was used as loading control. **(G)** Semiquantitative 3'RACE-PCRs upon *Srsf3* KD and transient CPSF6-myc expression. This experiment is representative of n=3.

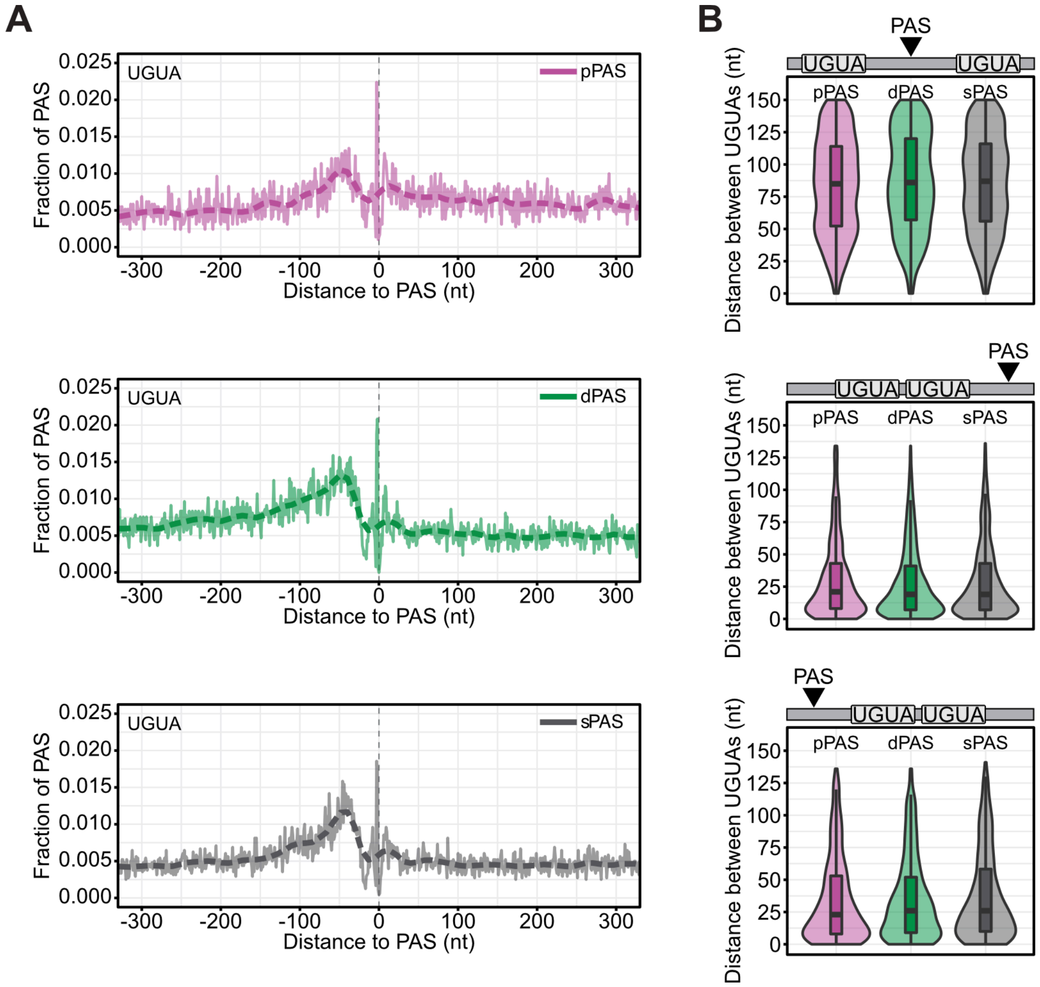

Fig. S9: UGUA motif pairs accumulate around PASs. (A) Enrichment of UGUA motifs around pPASs (top), dPASs (middle) and sPASs (bottom) used in P19 cells. Metaprofiles show fraction of PASs with UGUA motif per position in a 601-nt window. Dashed lines from loess smoothing. **(B)** Distances between UGUA motif pairs that flank (top) or occur upstream (middle) or downstream (bottom) of pPASs, dPASs and sPASs used in P19 cells.

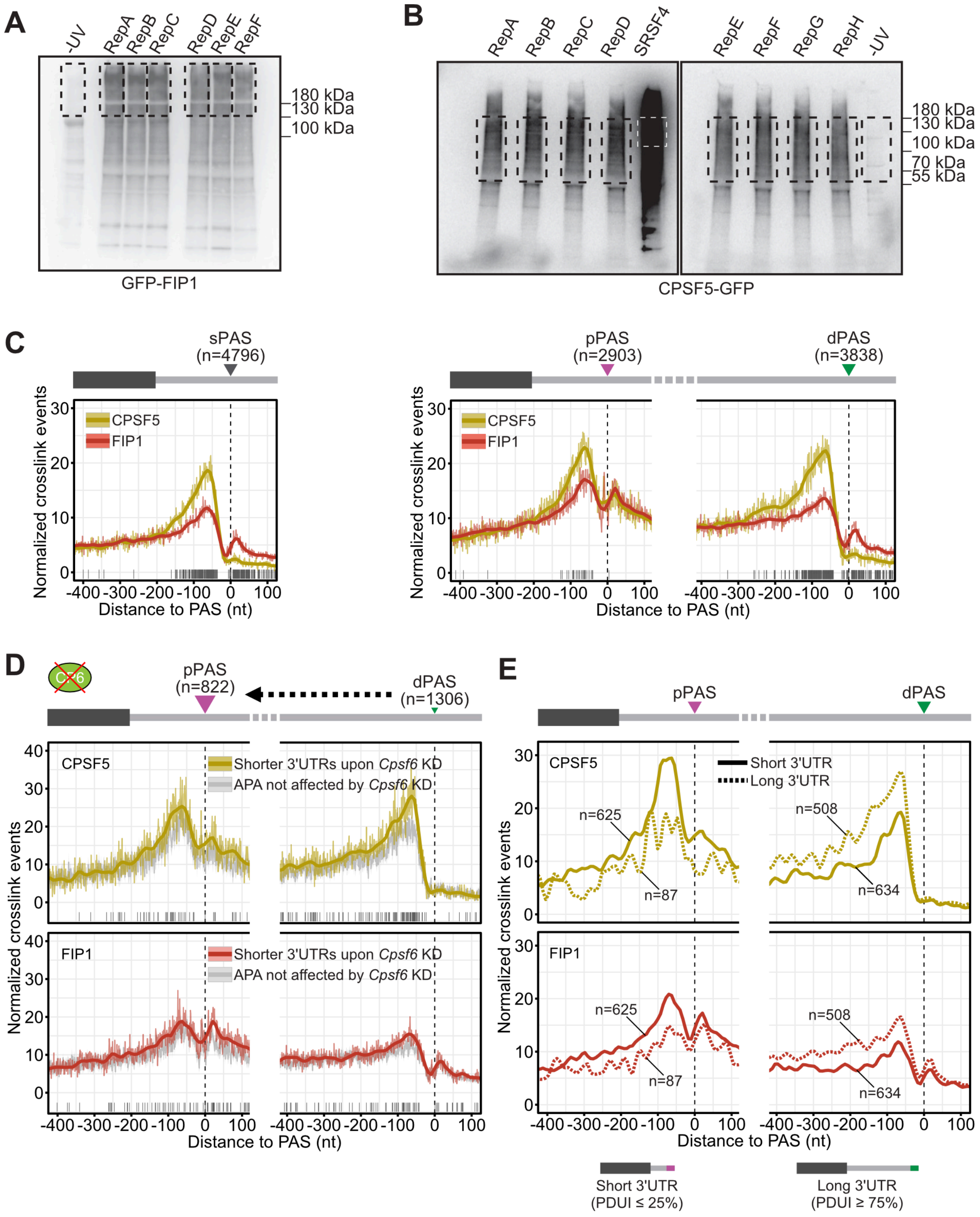

A
-UV
RepA RepB RepC
RepD RepE RepF
180 kDa
130 kDa
100 kDa
GFP-FIP1
B
RepA RepB RepC RepD SRSF4
RepE RepF RepG RepH -UV
180 kDa
130 kDa
100 kDa
70 kDa
55 kDa
CPSF5-GFP
C
sPAS
(n=4796)
pPAS
(n=2903)
dPAS
(n=3838)
CPSF5
FIP1
Normalized crosslink events
Distance to PAS (nt)
D
Cf6
pPAS
(n=822)
dPAS
(n=1306)
CPSF5
Shorter 3'UTRs upon Cpsf6 KD
APA not affected by Cpsf6 KD
FIP1
Normalized crosslink events
Distance to PAS (nt)
E
pPAS
dPAS
CPSF5
Short 3'UTR
Long 3'UTR
n=625
n=87
n=508
n=634
FIP1
Normalized crosslink events
Distance to PAS (nt)
Short 3'UTR
(PDUI ≤ 25%)
Long 3'UTR
(PDUI ≥ 75%)

Fig. S10: SRSF3-regulated pPASs show dual CFIm binding motifs downstream and enclosing pPAS and upstream of dPAS. (A&B) Autoradiographs of iCLIP experiments using α-GFP antibodies to pull down GFP-FIP1 (A) and CPSF5-GFP (B). Crosslinked RNA was labeled with ^{32}P. Non-crosslinked samples (-UV) served as controls. Cut bands are indicated with dashed squares. **(C)** Metaprofiles of normalized iCLIP crosslink events of FIP1 and CPSF5 in a window of -400 nt to 100 nt around sPASs (left) as well as pPASs and dPASs (right). Raw signal and loess smoothing are shown. Marks below the signals indicate positions with a significant signal difference between CPSF5 and FIP1 as inferred from two-proportions z-tests with FDR ≤ 0.01. **(D)** Metaprofiles of normalized iCLIP crosslink events of CPSF5 and FIP1 around pPASs and dPASs of CPSF6 targets compared to randomly sampled PASs that are not affected by *Cpsf6* KD. Raw signal and loess smoothing are shown. Significant differences in CPSF5 and FIP1 binding at CPSF6 targets compared to random sampled PASs are computed by a z-score approach. Positions with a FDR ≤ 0.01 are indicated by marks below the signals. **(E)** Metaprofiles of normalized iCLIP crosslink events of CPSF5 and FIP1 on genes that predominantly use their pPAS (solid) or dPAS (dashed) in P19 wt cells. Loess smoothing is shown.

Supplementary Tables

Additional file 2: Table S1. Genes with significant changes in 3′UTR length upon KD of *Srsf3* and *Srsf7* quantified with DaPars. Information on 686 SRSF3 targets and 134 SRSF7 targets with absolute distal polyA site usage index [|ΔPDUI|] ≥ 5% and false discovery rate (FDR) ≤ 0.1. Additional information from the DaPars output: Ensembl transcript IDs, gene names and genomic coordinates of the underlying 3′UTR; expression (exp) and PDUI for each replicate and mean over conditions; PDUI_Group_diff, ΔPDUI (KD over Ctrl); *P* value and adjusted *P* value (FDR).

Additional file 3: Table S2. Genes with significant changes in 3′UTR length upon KD of *Srsf3* and *Srsf7* quantified with MISO. Information on 273 SRSF3 targets and 89 SRSF7 targets with differences in tandem 3′UTR usage > 5% and Bayes factor (BF) > 5.

Additional file 4: Table S3. SRSF3 RNPome. Information on 832 proteins quantified by mass spectrometry.

Additional file 5: Table S4. Genes with significant differential expression upon P19 differentiation into neural cells quantified with DESeq2. Information on 17,134 genes with adjusted *P* value ≤ 0.1. Additional information from the DESeq2 output: Base_Mean, average normalized read count across all samples. log2_Fold_Change, log_2-transformed foldchange (Diff/Undiff).

Additional file 6: Table S5. Genes with significant changes in 3′UTR length upon P19 differentiation into neural cells quantified with DaPars. Information on 1,851 genes with |ΔPDUI| ≥ 5% and FDR ≤ 0.1. Additional information from the DaPars output: Ensembl transcript IDs, gene names and genomic coordinates of the underlying 3′UTR; expression (exp) and PDUI for each replicate and mean over conditions; PDUI_Group_diff, ΔPDUI (KD over Ctrl); *P* value and adjusted *P* value (FDR).

Additional file 7: Table S6. Genes with significant change in gene expression upon KD of *Srsf3* and *Srsf7* quantified with DESeq2. Adjusted *P* value ≤ 0.1. Additional information from the DESeq2 output: Base_Mean, average normalized read count across all samples. log2_Fold_Change, log_2-transformed foldchange (KD/control).

Additional file 8: Table S7. Genes with significant changes in 3′UTR length upon KD of *Cpsf6* quantified with DaPars. Information on 3907 genes with |ΔPDUI| ≥ 5% and FDR ≤ 0.1. Additional information from the DaPars output: Ensembl transcript IDs, gene names and genomic coordinates of the underlying 3′UTR; expression (exp) and PDUI for each replicate and mean over conditions; PDUI_Group_diff, ΔPDUI (KD over Ctrl); *P* value and adjusted *P* value (FDR).

Table S8: Unique crosslink events and positions derived from merged iCLIP replicates.

Protein	Unique crosslink events	Unique positions
SRSF3	16,458,431	13,022,949
SRSF7	19,177,075	14,616,952
CPSF5	1,851,266	1,659,525
FIP1	3,759,237	3,242,512

Table S9: Primers used in this study.

Primer	Sequence (5′-3′)	Tm [°C]	Purpose
3'RACE_PCR_rev	CCAGTGAGCAGAGTGACGAGGACTCGAGCTCAAGC	63	3′RACE-PCR
3'RACE-RT-Anchored	CCAGTGAGCAGAGTGACGAGGACTGAGCTCAAGCT TTTTTTTTTTTTTTTTTTV	63	3′RACE-PCR
3′RACE_Ddx21_fwd	CACCCTGCTGACAAAGCCCGA	63	3′RACE-PCR
3′RACE_Rab11a_fwd	GCGTCTCTTCCCCTAGAAGGCTGT	63	3′RACE-PCR
3′RACE_Anp32e_fwd	CCTGGGCGCTGGAGAGCGAT	63	3′RACE-PCR
3′RACE_Hspa4_fwd	CCAAACCCAAAGTGGAACCCCCA	63	3′RACE-PCR
3′RACE_Pphln1_fwd	TGCGAGACTTTCGGGATGGTG	63	3′RACE-PCR
3′RACE_Tnpo3_fwd	CACCTAAGAGTGGGAGGCTGC	63	3′RACE-PCR
mCherry3'_fwd	GCGCCTACAACGTCAACATCAAG	63	3′RACE-PCR
PCR_Luc2_PAS-Seq_fwd	AGGTGCCTAAAGGACTGACCGG	63	3′RACE-PCR
RS_SRSF3_fw	GACCCGGGAGGAGTCCTCCACCTC	63	Cloning
RS_SRSF3_rev	GACCCGGGCTATTTCCTTTCATTTGACC	63	Cloning
RS_SRSF7_fw	GACCCGGGAGCCGACGAAGAAGAAGC	63	Cloning
RS_SRSF7_rev	GACCCGGGTCAGTCCATTCTTTCTGGACT	63	Cloning
RS_SRSF3_S-to-D_fw	GACCCGGGAGGGACCCTCCACCTC	63	Cloning
RS_SRSF3_S-to-D_rev	GACCCGGGCTATTTCCTTTCATTGTCCC	63	Cloning
RS_SRSF7_S-to-D_fw	GACCCGGGGACCGACGAAGAAGAGAC	63	Cloning
RS_SRSF7_S-to-D_rev	GACCCGGGTCAGTCCATTCTTTCTGGGTC	63	Cloning
RS_SRSF3_S-to-A_fw	GACCCGGGAGGGCCCCTCCACCTC	63	Cloning
RS_SRSF3_S-to-A_rev	GACCCGGGCTATTTCCTTTCATTGGCCC	63	Cloning
RS_SRSF7_S-to-A_fw	GACCCGGGGCCCGACGAAGAAGAGCC	63	Cloning
RS_SRSF7_S-to-A_rev	GACCCGGGTCAGTCCATTCTTTCTGGGGC	63	Cloning
gBlocks_SRSF3_fwd	ATGCATCGTGATTCCTGTCCCTTGG	63	Cloning
gBlocks_SRSF3-RA_rev	CTATTTCCTTTCATTGGCCCTAGCTCGG	63	Cloning
gBlocks_SRSF3-RD_rev	CTATTTCCTTTCATTGTCCCTGTCTCGGT	63	Cloning
gBlocks_SRSF7_fwd	ATGTCACGCTACGGGCGG	63	Cloning
gBlocks_SRSF7-RA_rev	TCAGTCCATTCTTTCTGGAGCTGCG	63	Cloning
gBlocks_SRSF7-RD_rev	TCAGTCCATTCTTTCTGGGTCTGCGT	63	Cloning
GA_pmCherry_for	AGGCGTAAATTGTAAGCGTTAATATTTTGT	63	Cloning
GA_pmCherry_rev	CTACTTGTACAGCTCGTCCATGCC	63	Cloning
GA_pLuc2_fwd	AAATCGATAAGGATCCGTTTGCGTATTGGG	63	Cloning
GA_pLuc2_rev	TTACACGGCGATCTTGCCGCCCT	63	Cloning
SRSF3-RA-KpnI_rev	GCGGTACCACTTTCCTTTCATTGGCCCTAGCTCGG G	63	Cloning
SRSF3-RD-KpnI_rev	GCGGTACCACTTTCCTTTCATTGTCCCTGTCTCGG TCAC	63	Cloning
SRSF7-RA-KpnI_rev	GCGGTACCACGTCCATTCTTTCTGGAGCTGCGG	63	Cloning
SRSF7-RD-KpnI_rev	GCGGTACCACGTCCATTCTTTCTGGGTCTGCGTCT CTGTG	63	Cloning
GA_Luc_Ddx21_fwd	CAAGATCGCCGTGTAAGTAGAGGCCAGAAGGGACT GTTCC	63	Cloning
GA_Luc_Ddx21_rev	AAACGGATCCTTATCGATTTAGGATTGCGTTCATT TTTATGACAATTTAGCCAAGG	63	Cloning
GA_mCherry_Ddx21_fwd	GACGAGCTGTACAAGTAGGTAGAGGCCAGAAGGGA CTGTTCCC	63	Cloning
GA_mCherry_Ddx21_rev	CGCTTACAATTTACGCCTAGGATTGCGTTCATTTT TATGACAATTTAGCCAAGG	63	Cloning

(continued on next page)

(continued from previous page)

Primer	Sequence (5′-3′)	Tm [°C]	Purpose
**GA_Luc_Anp32e_fwd**	CAAGATCGCCGTGTAAACCTCCAGGACCAGGCCAC	63	Cloning
**GA_Luc_Anp32e_rev**	AAACGGATCCTTATCGATTTTACCACCACTGCTGTTGGAGTATGAGT	63	Cloning
**GA_mCherry_Anp32e_fwd**	GACGAGCTGTACAAGTAGACCTCCAGGACCAGGCCAC	63	Cloning
**GA_mCherry_Anp32e_rev**	CGCTTACAATTTACGCCTTACCACCACTGCTGTTGGAGTATGAGT	63	Cloning
**GA_Luc_Rab11a_fwd**	CAAGATCGCCGTGTAAGGCGTCTCTTCCCCTAGAAGGC	63	Cloning
**GA_Luc_Rab11a_rev**	AAACGGATCCTTATCGATTTCTCTGCAGATCTAAAGTCTACCTGAACTGACAGT	63	Cloning
**GA_mCherry_Rab11a_fwd**	GACGAGCTGTACAAGTAGGGCGTCTCTTCCCCTAGAAGGC	63	Cloning
**GA_mCherry_Rab11a_rev**	CGCTTACAATTTACGCCTCTCTGCAGATCTAAAGTCTACCTGAACTGACAGT	63	Cloning
**Ddx21_UTR_Vector_fwd**	ACCAACCATGGATCTGCCTATCTTGG	63	Cloning
**Ddx21_UTR_Vector_rev**	TGCAAGAAAGGGGGCGCTGAG	63	Cloning
**Ddx21_strongpPAS_Frag_for**	GCCCCCTTTCTTGCAGAGAGGCTTCTGAACTGTCCACTAACCA	63	Cloning
**Ddx21_strongpPAS_Frag_rev**	GCAGATCCATGGTTGGTTTTATTTTGTAAATCTAAAAGTATGTTGGAAAACGATGCAATGAATTCT	63	Cloning
**Ddx21_pPASmutant_Frag_rev**	GCAGATCCATGGTTGGTCTTACTTTGTAAATCTAAAAGTATGTTGGAAAACGATGCAATG	63	Cloning
**Ddx21_noSR3motif_Frag_for**	GCCCCCTTTCTTGCAGAGAGGATTCTGAACTGTGAACTAACGATCACAA	63	Cloning
**Ddx21_noSR3motif_Frag_rev**	GCAGATCCATGGTTGGTTTTACTTTGTAAATCTAAAAGTATGTTCGAAAACGATCCAATGAA	63	Cloning
**Ddx21_noSR7motif_Frag_fwd**	GCCCCCTTTCTTGCACAGCGGCTTCTCAACTGTCCACTAACC	63	Cloning
**Ddx21_noSR7motif_Frag_rev**	GCAGATCCATGGTTGGTTTTACTTTGTAGATGTAAAAGTATGTTGGAAAACGATGCAAT	63	Cloning
**Calr_fwd**	TCAAGTCCGGGACAATCTTTG	63	Test PCR
**Calr_rev**	TCCTCTTTACGCTTCTTGTCCTC	63	Test PCR
**Splicing_CPSF6_Ex4_fwd**	TGGTCAGAGTCCTGTTGTAACTCCA	63	RT-PCR
**Splicing_CPSF6_Exon7_rev**	CTCAGCTTCACTCAGAGGTGTTCTTGCA	63	RT-PCR
**esi_SRSF7_fwd**	CGTAATACGACTCACTATAGGGATTCGCCTTTGTGGAATTTG	63	esiRNAs
**esi_SRSF7_rev**	CGTAATACGACTCACTATAGGGCTTGAGCGGGATTGGAAATA	63	esiRNAs
**esi_mSRSF3_1_fwd**	CGTAATACGACTCACTATAGGGAGAGAAATCACAAGCCGTCTC	63	esiRNAs
**esi_mSRSF3_1_rev**	CGTAATACGACTCACTATAGGGAGAGGCTTGTGTTCACAGCAG	63	esiRNAs
**esi_Luc_fwd**	TAATACGACTCACTATAGGGAGATTCTTCATGCCCGTGTTGGGTGC	63	esiRNAs
**esi_Luc_rev**	TAATACGACTCACTATAGGGAGAAATCCGGTACTGCCACTACTGTTCATGA	63	esiRNAs
**esi_mCPSF6_R3_fwd**	TAATACGACTCACTATAGGGAGATACGCGGATGTGGGTGAAGAG	63	esiRNAs
**esi_mCPSF6_R3_rev**	TAATACGACTCACTATAGGGAGAGACTCAGGAACTGCTTATTGCATGG	63	esiRNAs
**qPCR_CPSF6_fwd**	ACCTTTTCCAGCTGGACAAACTCC	63	RT-qPCR
**qPCR_CPSF6_rev**	GGAGCAAGTGTGAGGGGAGG	63	RT-qPCR
**qPCR_U6-1_fwd**	GCTCGCTTCGGCAGC	63	RT-qPCR
**qPCR_U6-1_rev**	AAATATGGAACGCTTCACGAATT	63	RT-qPCR

Additional Files

Additional file 9: Source data for main figures. Uncropped gel images and additional replicates for analyses shown in Fig. 1E, 2E, 2G, 3A, 3B, 3D, 3E, 3G, 3H, 4C, 4E, 4F, 5B, 6D and 6E.

Additional file 10: Source data for supplementary figures. Uncropped gel images and additional replicates for analyses shown in Fig. S1A, S1D, S3A-D, S4E-I, S5A-C, S5E-G, S6B, S6D-F, S7A, S7B, S8C, S8E-G, S10A and S10B.
