# Supplementary material for: SRSF3 and SRSF7 modulate 3′UTR length through suppression or activation of proximal polyadenylation sites and regulation of CFIm levels
Source: Genome Biol. 2021 Mar 11;22:82. doi: 10.1186/s13059-021-02298-y (PMC7948361; doi:10.1186/s13059-021-02298-y)
Supplement: Supplementary file 9 — Additional file 9. [file 13059_2021_2298_MOESM9_ESM.pdf]

source data Figure 1E

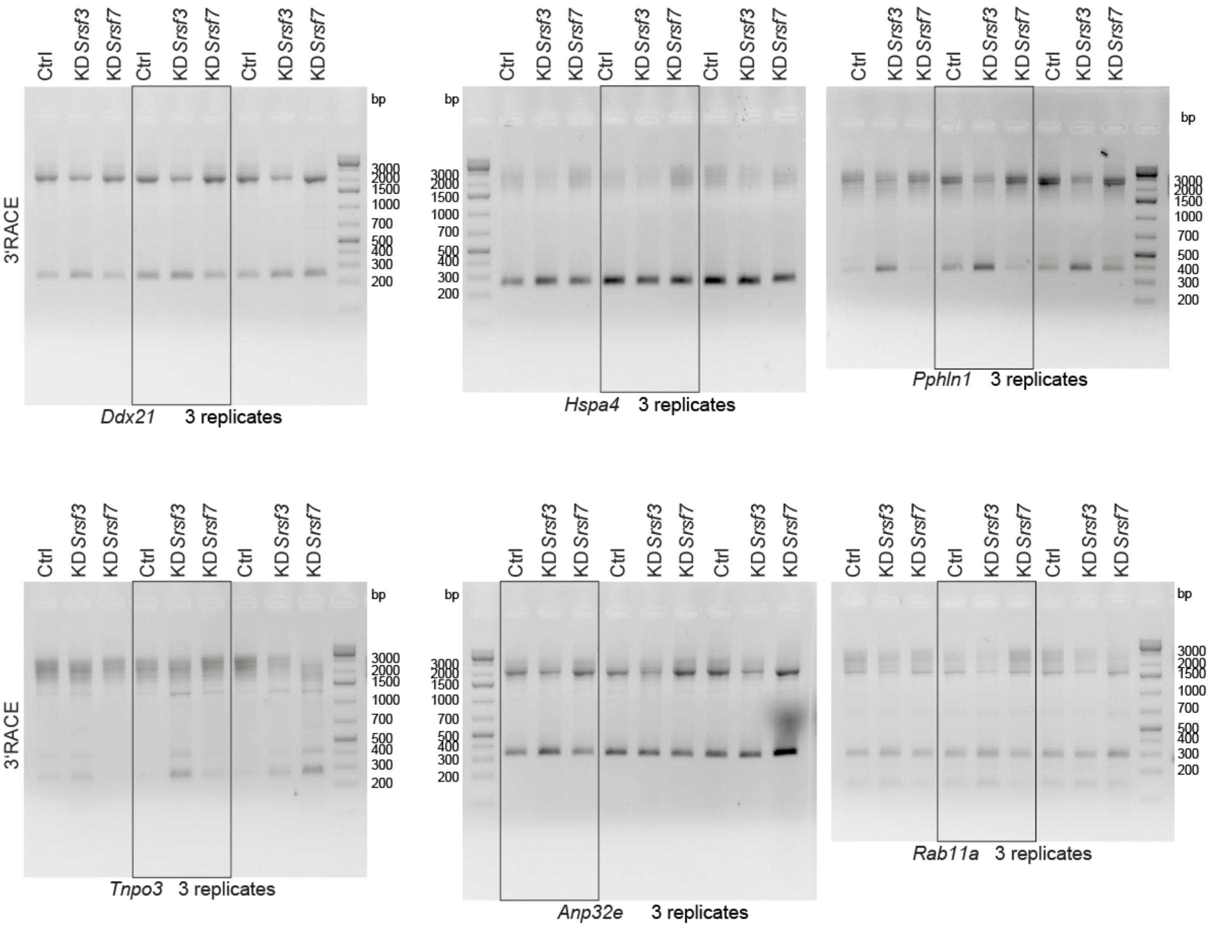

source data Figure 2E

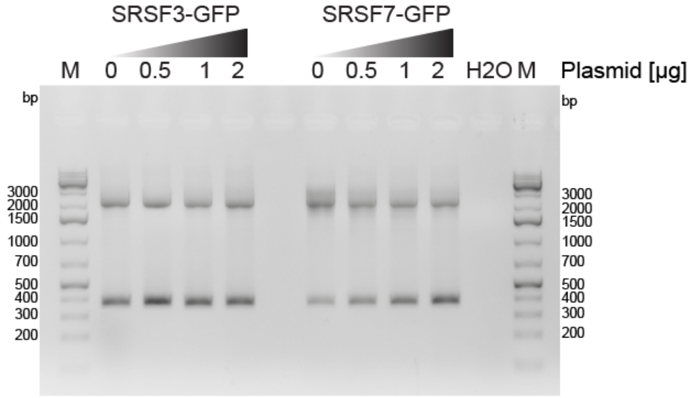

3‘RACE-PCR; *Luc-Ddx21*

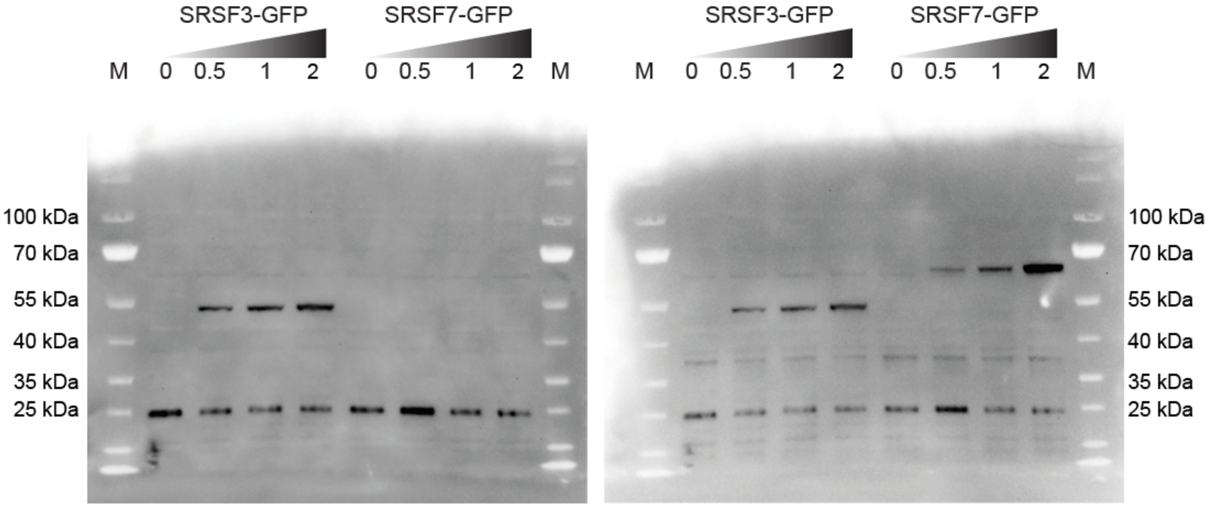

WB: α-SRSF3

WB: α-SRSF7

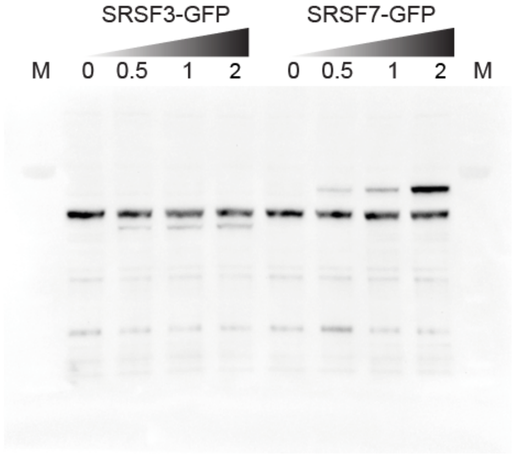

WB: α-αTUB

source data Figure 2G

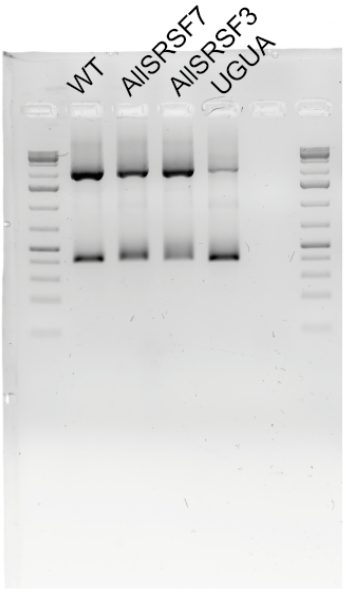

Replicate 1

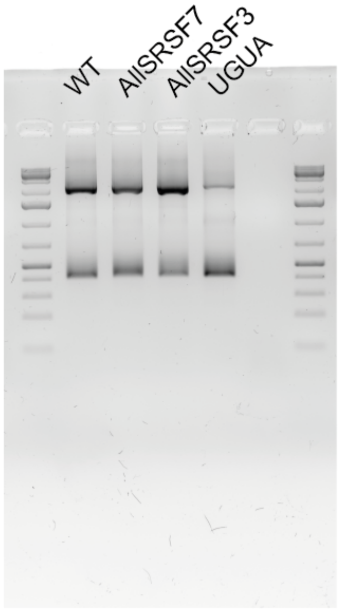

Replicate 2

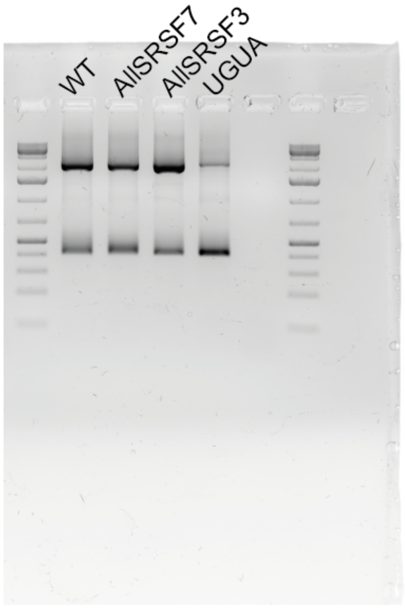

Replicate 3

source data Figure 3A

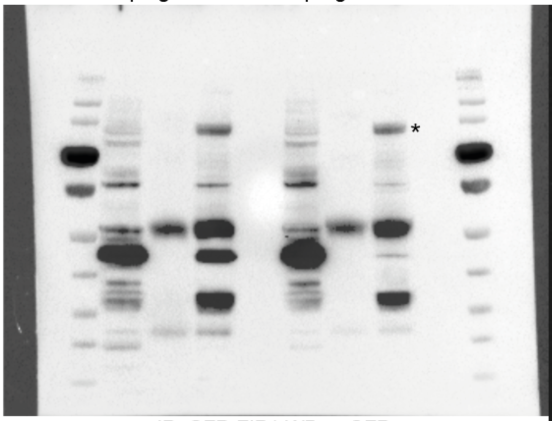

IP: GFP-FIP1,WB: α-GFP

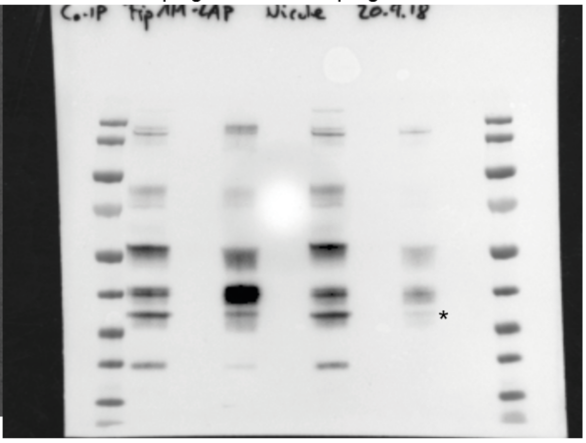

IP: GFP-FIP1, WB: α-mab1o4

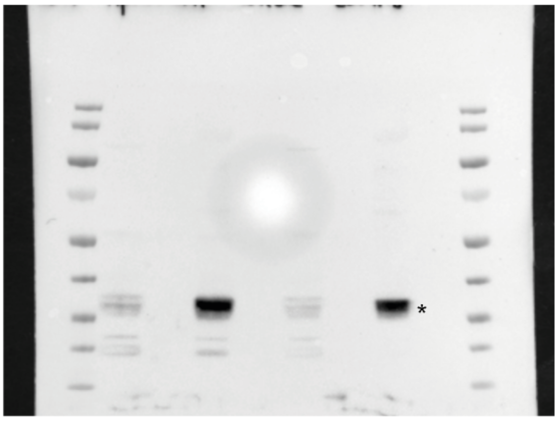

IP: GFP-FIP1, WB: α-SRSF7

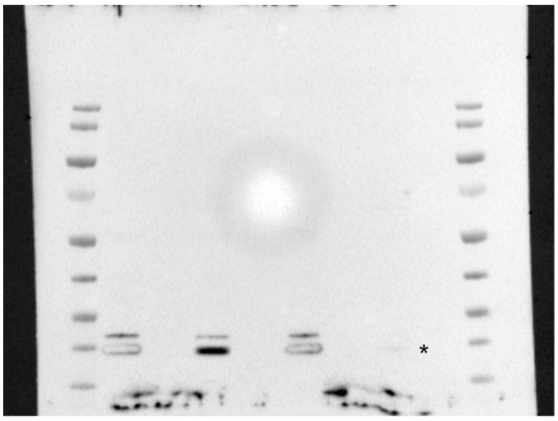

IP: GFP-FIP1, WB: α-SRSF3

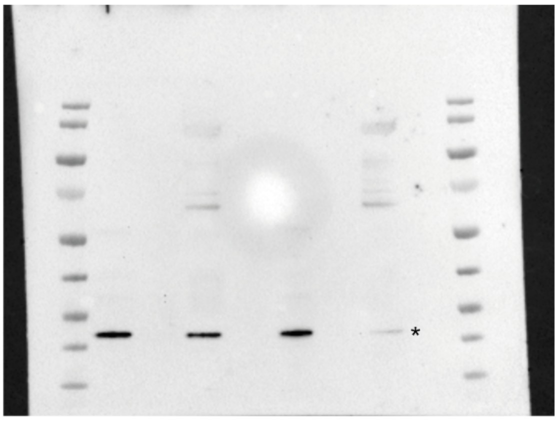

IP: GFP-FIP1, WB: α-CPSF5

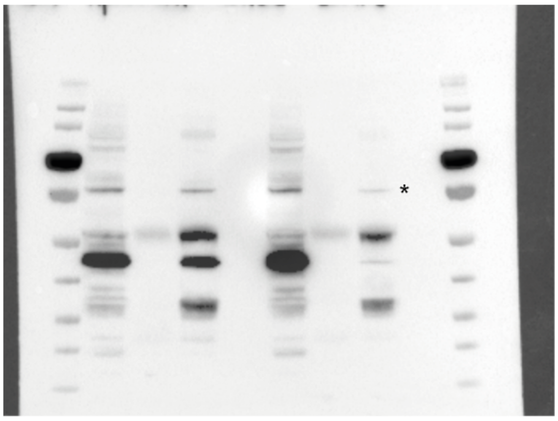

IP: GFP-FIP1, WB: α-CPSF6

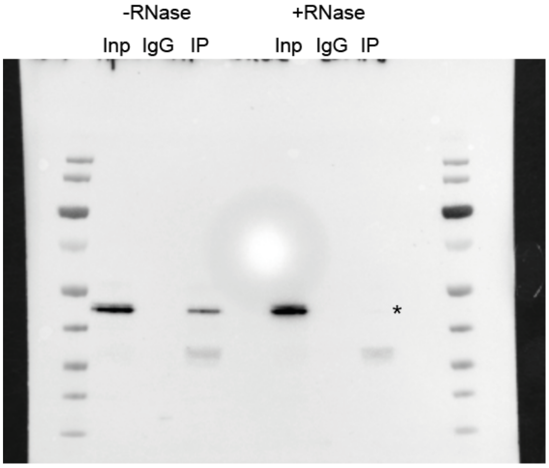

IP: GFP-FIP1, WB: α-PABPN1

source data Figure 3B

-RNase +RNase
Inp IgG IP Inp IgG IP

-RNase +RNase
Inp IgG IP Inp IgG IP

100 kDa
70 kDa
55 kDa
40 kDa
35 kDa
25 kDa

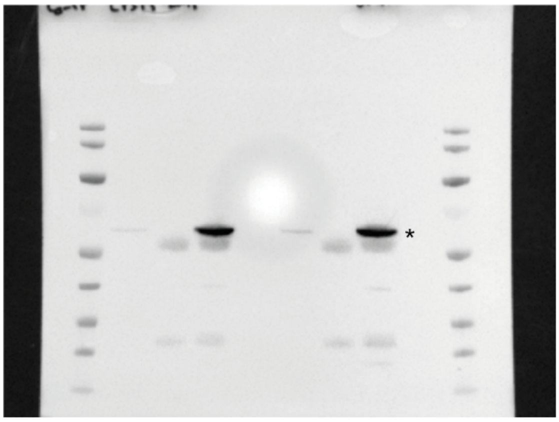

IP: CPSF5-GFP, WB: α-GFP

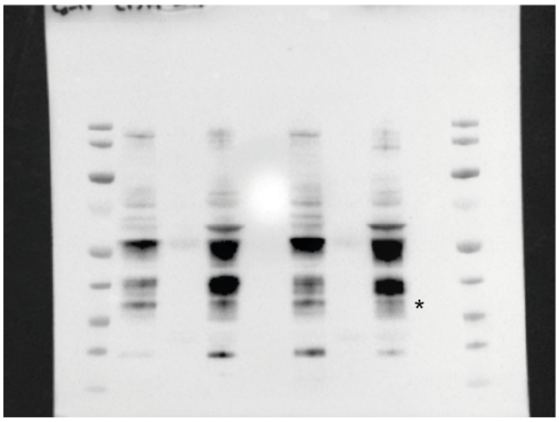

IP: CPSF5-GFP, WB: α-mab1o4

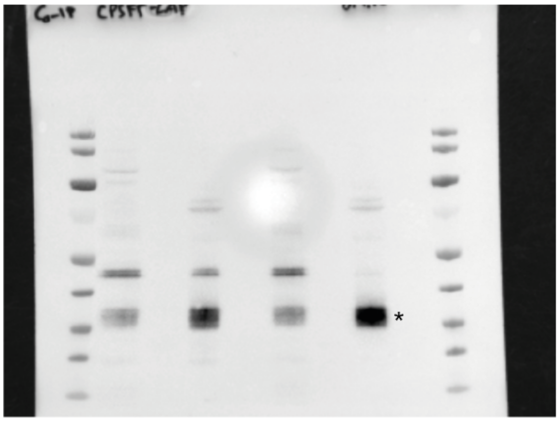

IP: CPSF5-GFP, WB: α-SRSF7

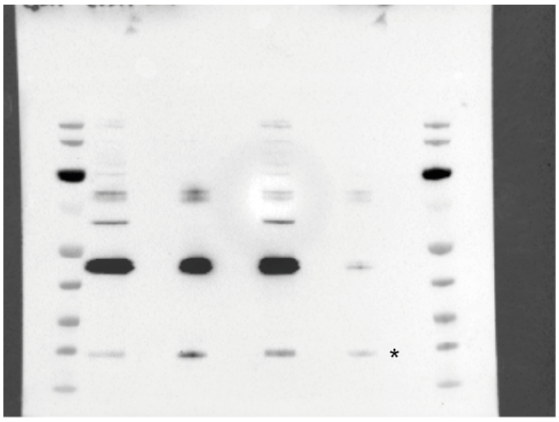

IP: CPSF5-GFP, WB: α-SRSF3

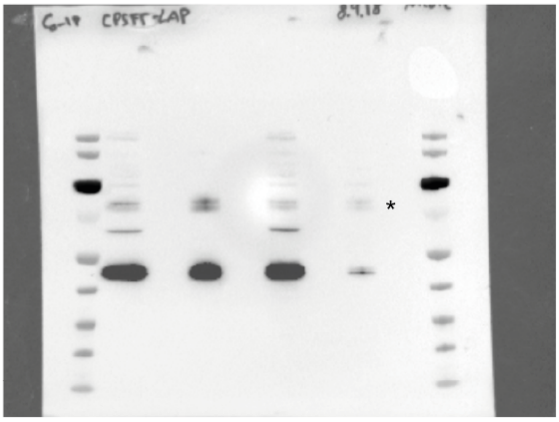

IP: CPSF5-GFP, WB: α-FIP1

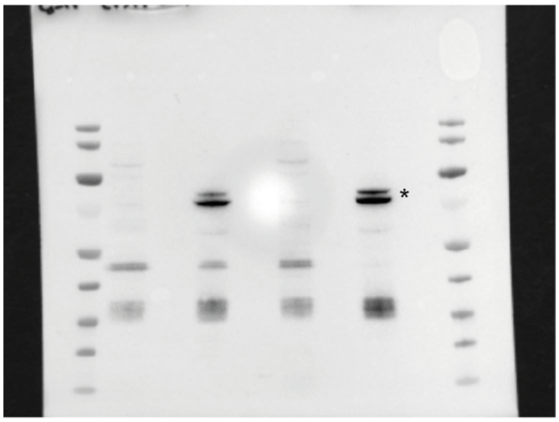

IP: CPSF5-GFP, WB: α-CPSF6

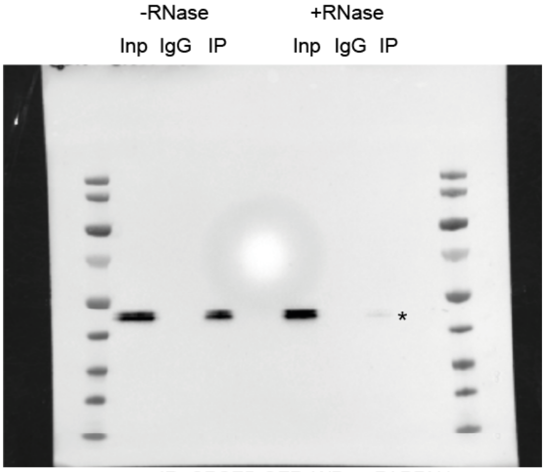

IP: CPSF5-GFP, WB: α-PABPN1

source data Figure 3D

-RNase +RNase

Inp IgG IP Inp IgG IP

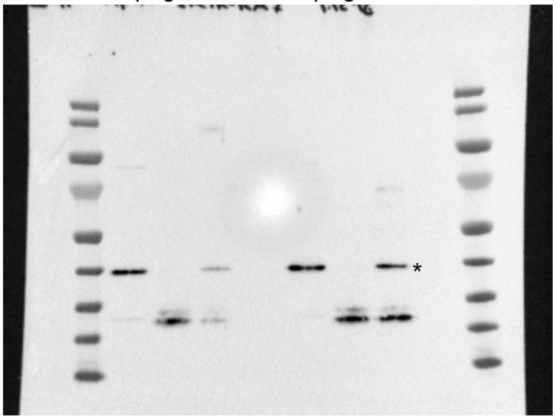

IP: GFP-FIP1, WB: α-TetR; **TetR-RA7**

-RNase +RNase

Inp IgG IP Inp IgG IP

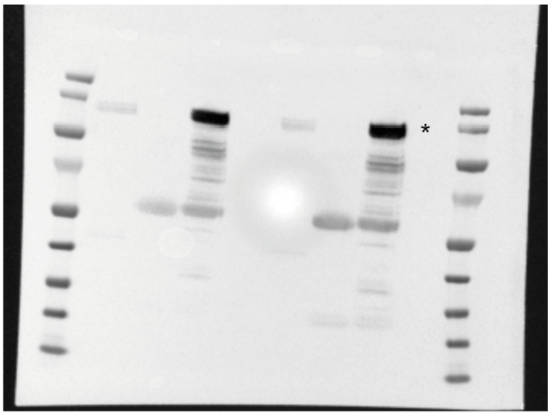

IP: GFP-FIP1, WB: α-GFP; **TetR-RD7**

-RNase +RNase

Inp IgG IP Inp IgG IP

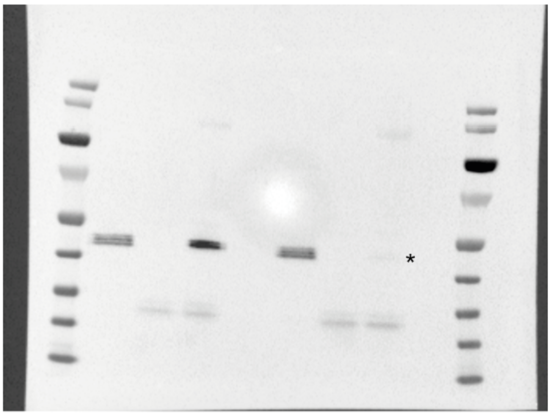

IP: GFP-FIP1, WB: α-PABPN1; **TetR-RD7**

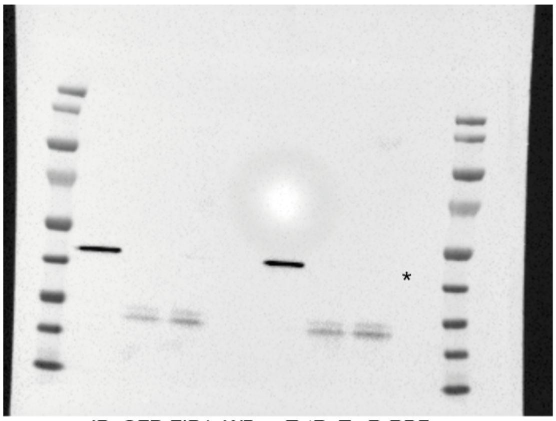

IP: GFP-FIP1, WB: α-TetR; **TetR-RD7**

source data Figure 3E

-RNase +RNase

Inp IgG IP Inp IgG IP

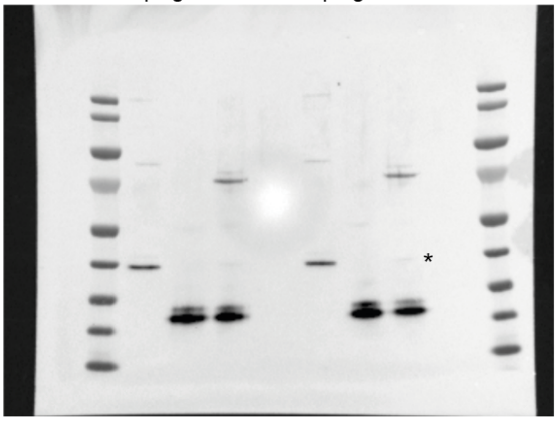

IP: CPSF5-GFP, WB: α-TetR; **TetR-RA7**

-RNase +RNase

Inp IgG IP Inp IgG IP

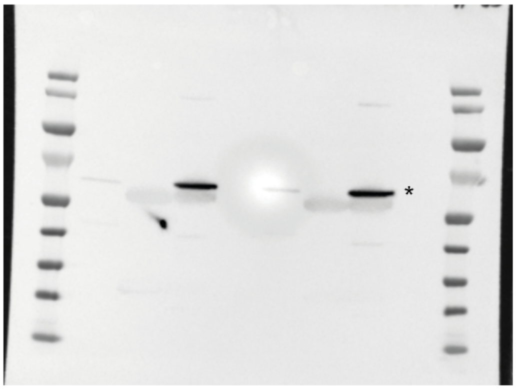

IP: CPSF5-GFP, WB: α-GFP; **TetR-RD7**

-RNase +RNase

Inp IgG IP Inp IgG IP

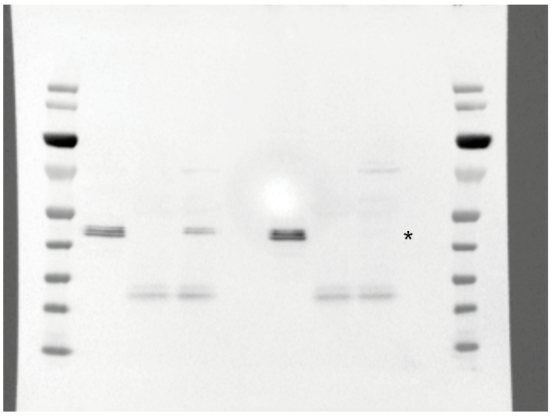

IP: CPSF5-GFP, WB: α-PABPN1; **TetR-RD7**

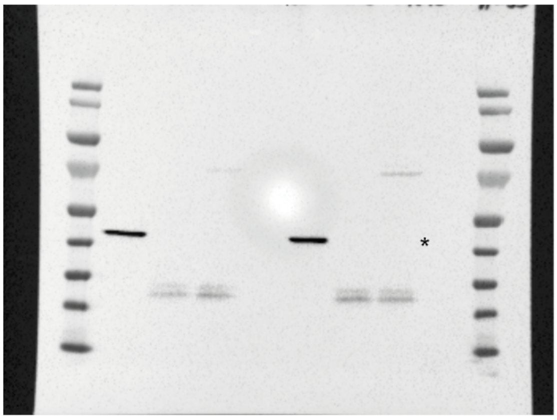

IP: CPSF5-GFP, WB: α-TetR; **TetR-RD7**

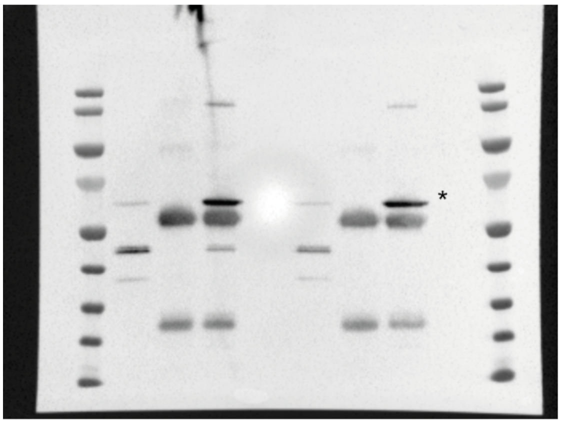

IP: CPSF5-GFP, WB: α-GFP; **TetR-RS3**

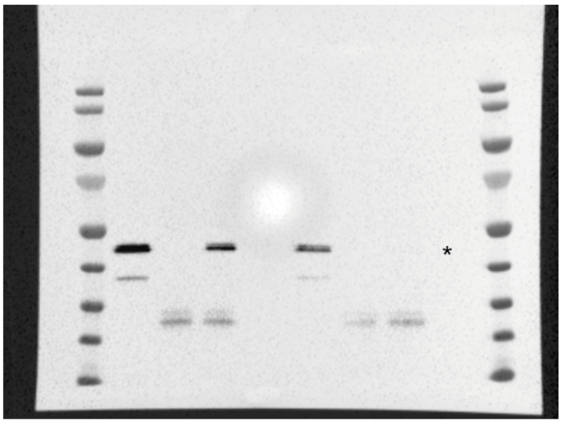

IP: CPSF5-GFP, WB: α-PABPN1; **TetR-RS3**

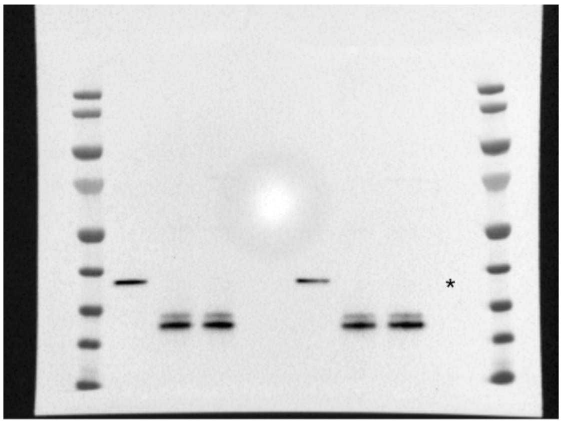

IP: CPSF5-GFP, WB: α-TetR; **TetR-RS3**

source data Figure 3G

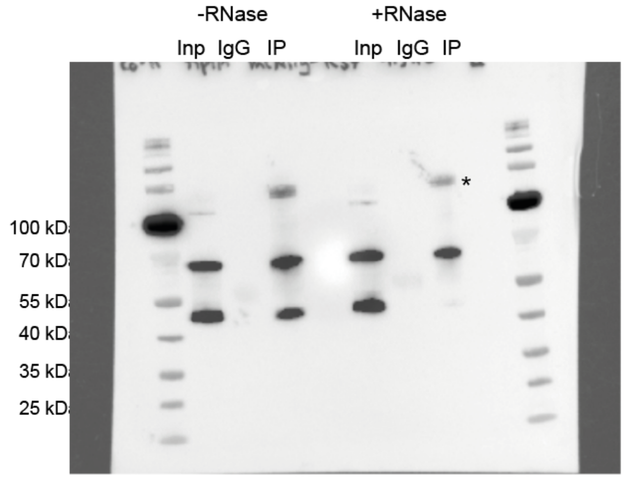

IP: GFP-FIP1, WB: α-GFP; **SRSF7-RS**

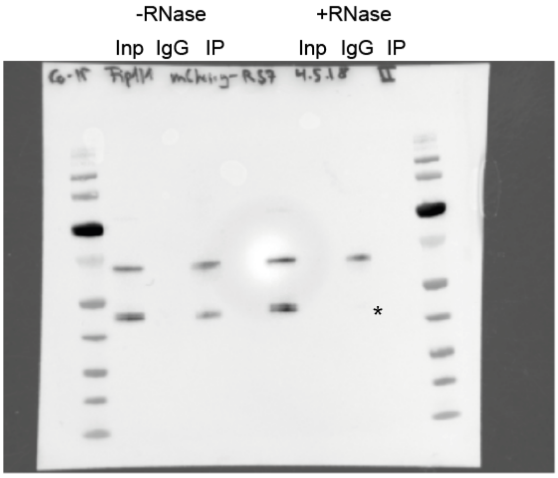

IP: GFP-FIP1, WB: α-PABPN1; **SRSF7-RS**

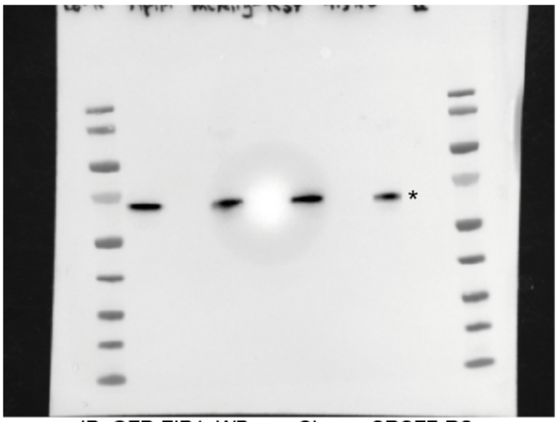

IP: GFP-FIP1, WB: α-mCherry; **SRSF7-RS**

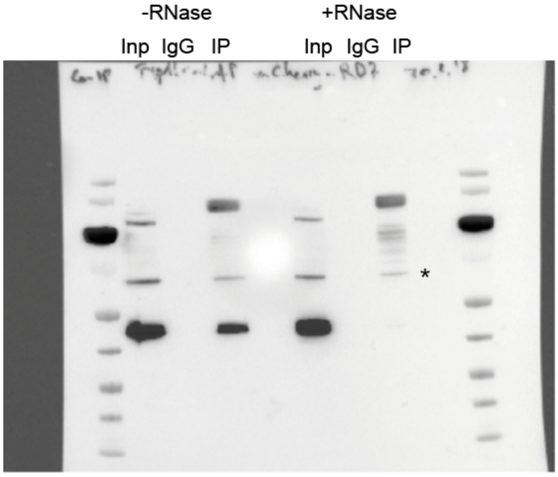

IP: GFP-FIP1, WB: α-mCherry; **SRSF7-RD**

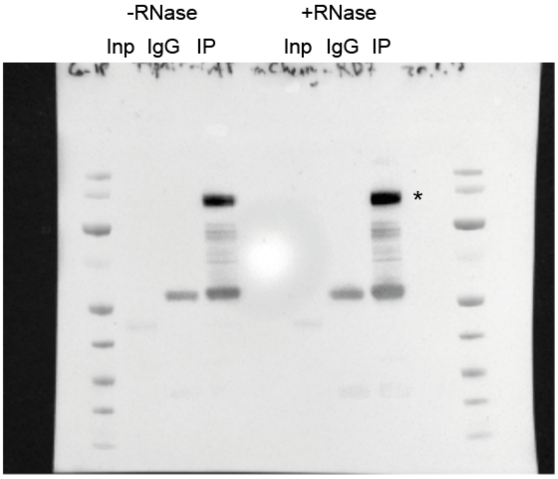

IP: GFP-FIP1, WB: α-GFP; **SRSF7-RD**

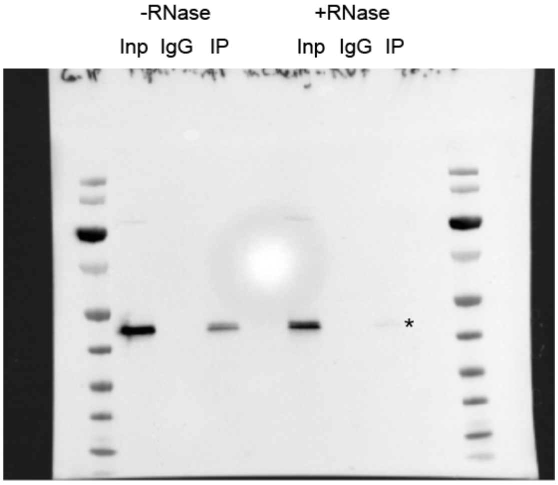

IP: GFP-FIP1, WB: α-PABPN1; **SRSF7-RD**

source data Figure 3H

-RNase +RNase

Inp IgG IP Inp IgG IP

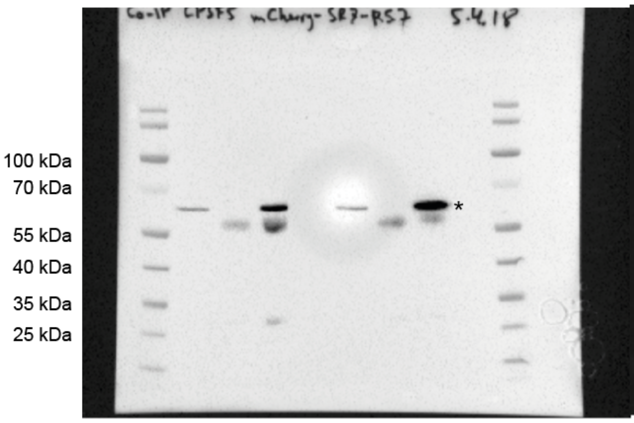

IP: CPSF5-GFP, WB: α-GFP; **SRSF7-RS**

-RNase +RNase

Inp IgG IP Inp IgG IP

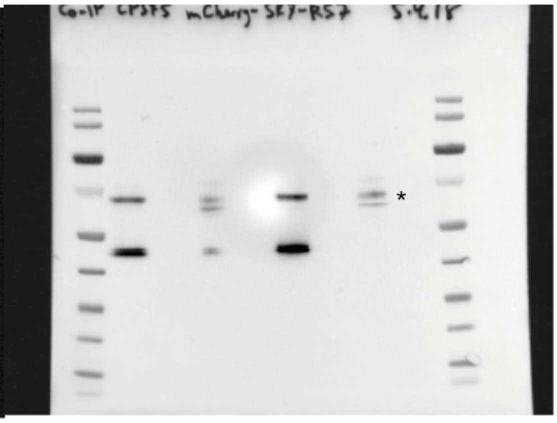

IP: CPSF5-GFP, WB: α-PABPN1; **SRSF7-RS**

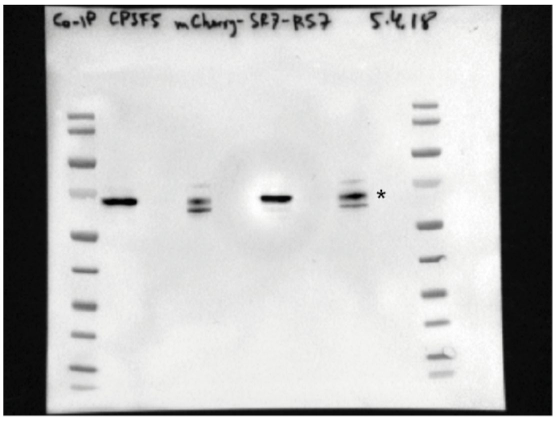

IP: CPSF5-GFP, WB: α-mCherry; **SRSF7-RS**

-RNase +RNase

Inp IgG IP Inp IgG IP

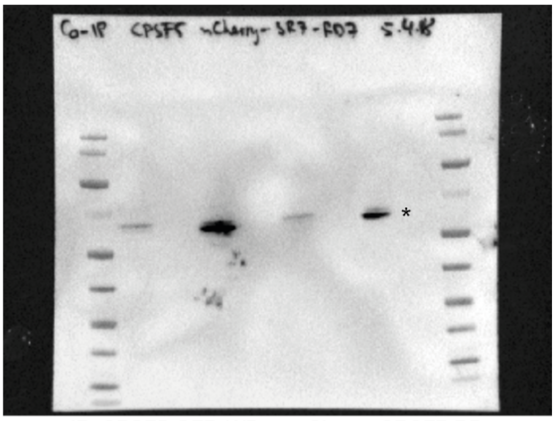

IP: CPSF5-GFP, WB: α-mCherry; **SRSF7-RD**

-RNase +RNase

Inp IgG IP Inp IgG IP

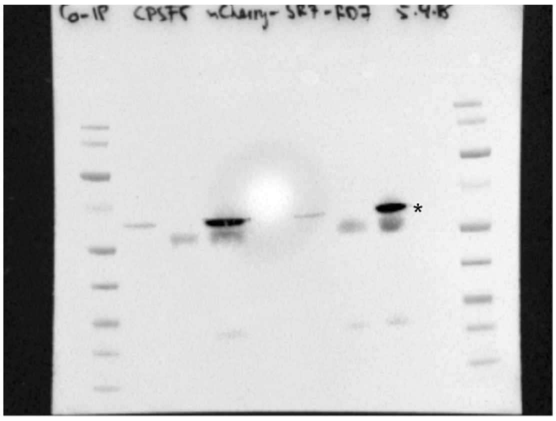

IP: CPSF5-GFP, WB: α-GFP; **SRSF7-RD**

-RNase +RNase

Inp IgG IP Inp IgG IP

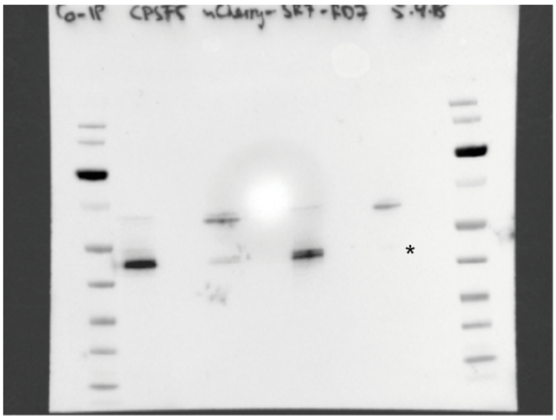

IP: CPSF5-GFP, WB: α-PABPN1; **SRSF7-RD**

source data Figure 4C

SRSF7 | Δ27aa | mutZn

Inp IgG IP | Inp IgG IP | Inp IgG IP

100 kDa
70 kDa
55 kDa
40 kDa
35 kDa
25 kDa

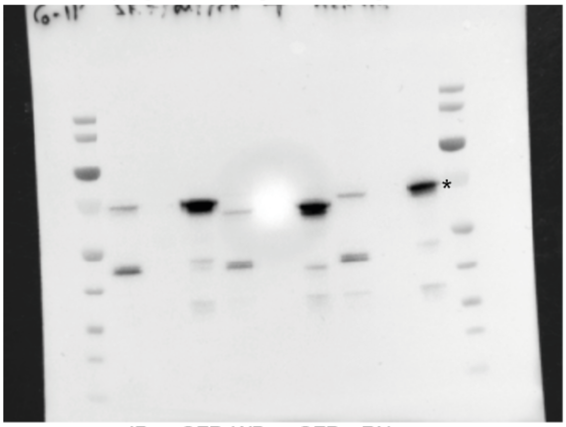

IP: α-GFP, WB: α-GFP, +RNase

SRSF7 | Δ27aa | mutZn

Inp IgG IP | Inp IgG IP | Inp IgG IP

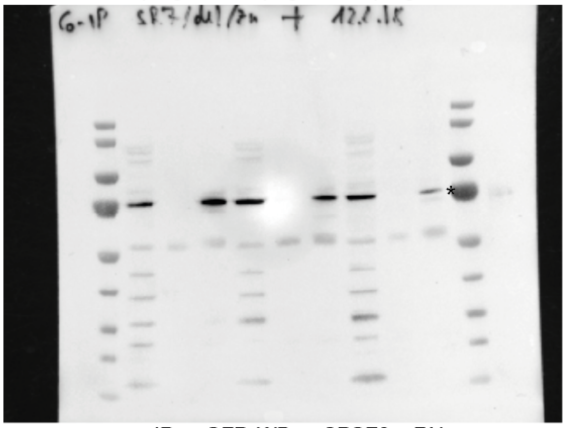

IP: α-GFP, WB: α-CPSF6, +RNase

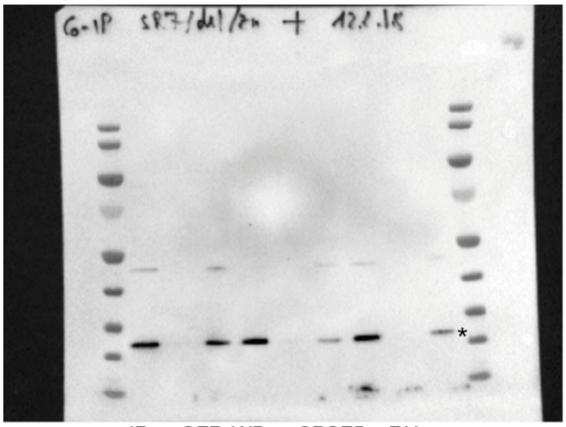

IP: α-GFP, WB: α-CPSF5, +RNase

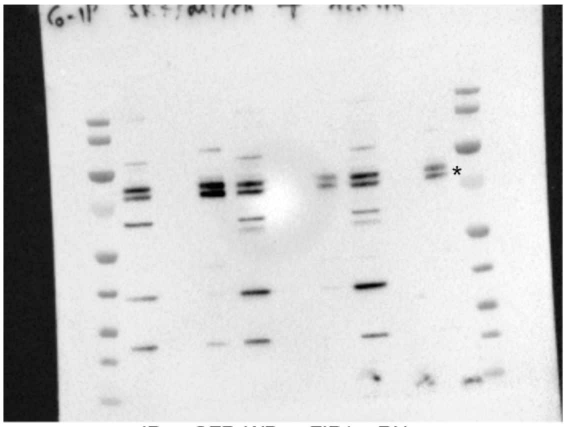

IP: α-GFP, WB: α-FIP1, +RNase

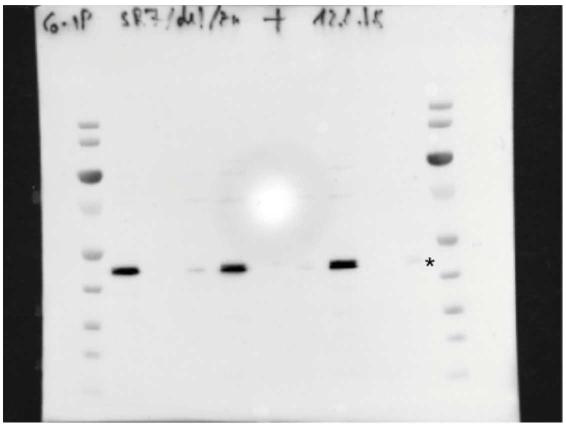

IP: α-GFP, WB: α-PABPN1, +RNase

source data Figure 4E

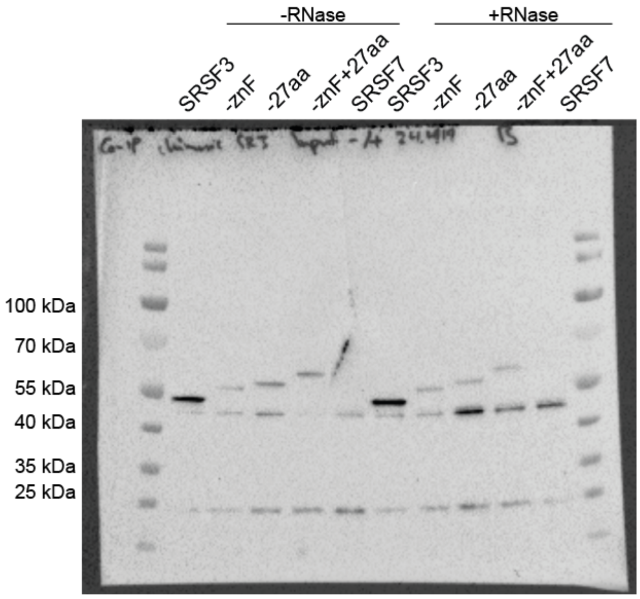

Input, IP: α-GFP, WB: α-SRSF3

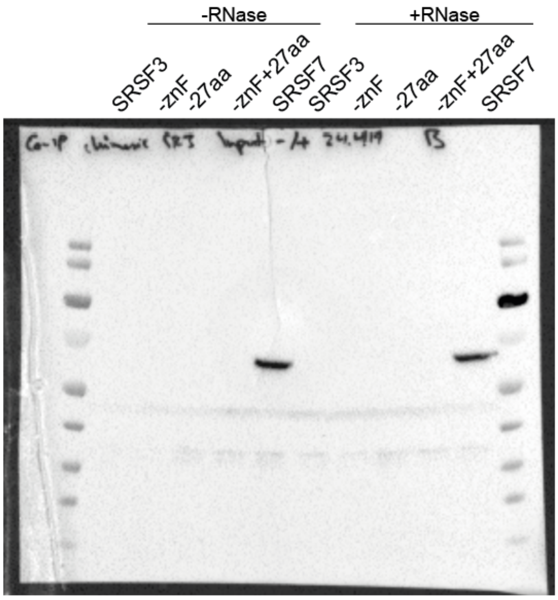

Input, IP: α-GFP, WB: α-SRSF7

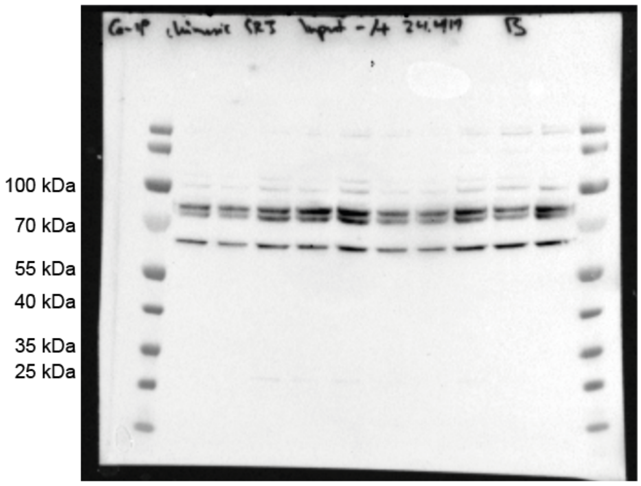

Input, IP: α-GFP, WB: α-FIP1

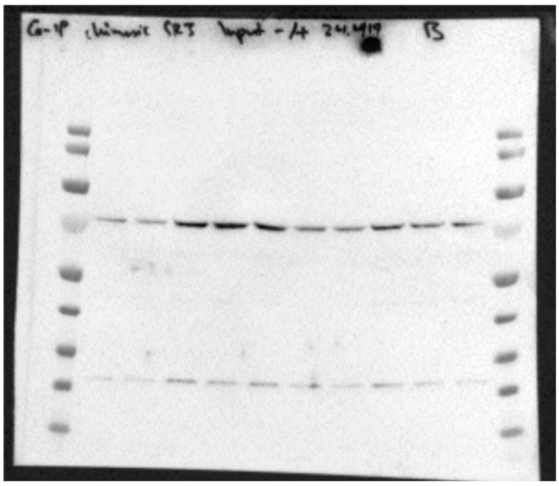

Input, IP: α-GFP, WB: α-CPSF6

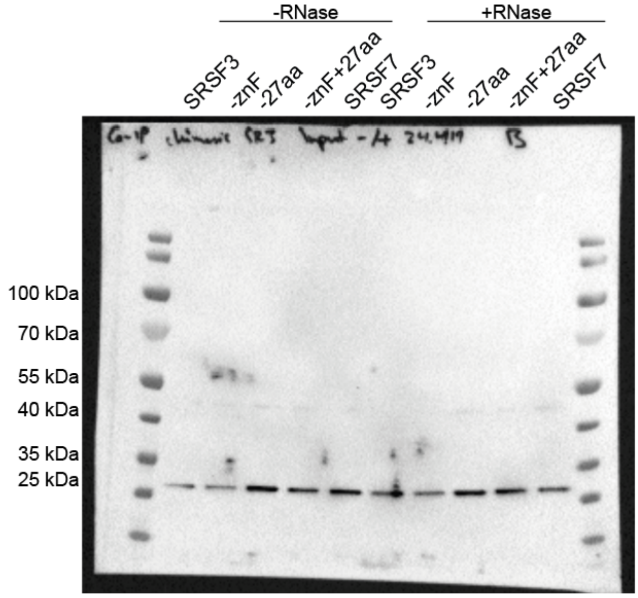

Input, IP: α-GFP, WB: α-CPSF5

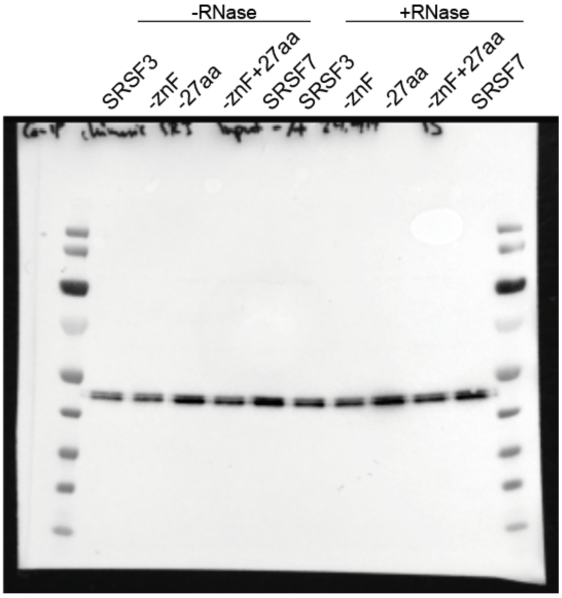

Input, IP: α-GFP, WB: α-PABPN1

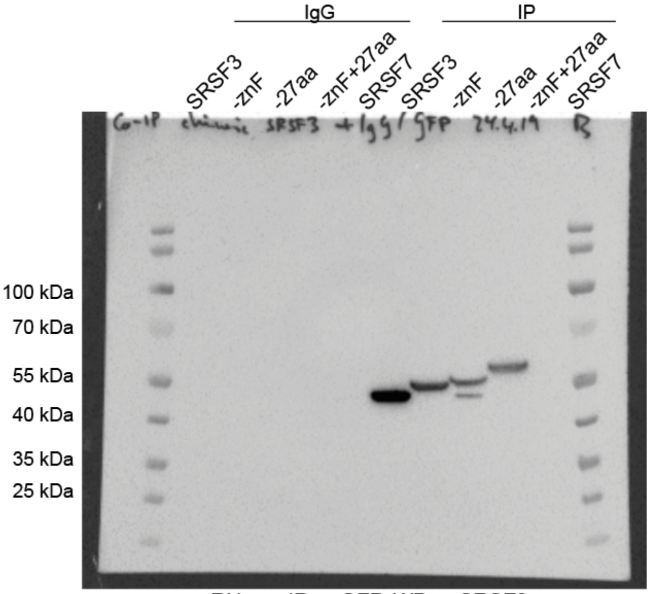

+RNase, IP: α-GFP, WB: α-SRSF3

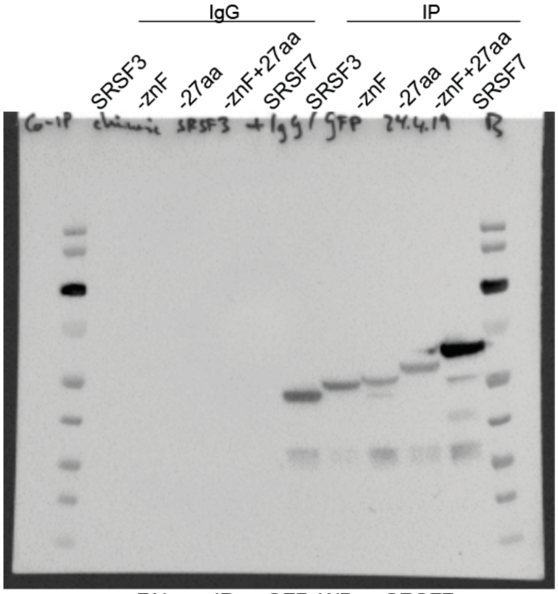

+RNase, IP: α-GFP, WB: α-SRSF7

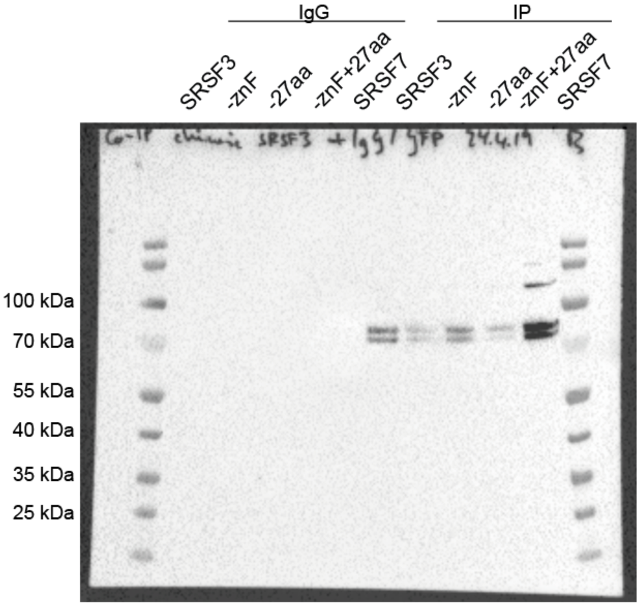

+RNase, IP: α-GFP, WB: α-FIP1

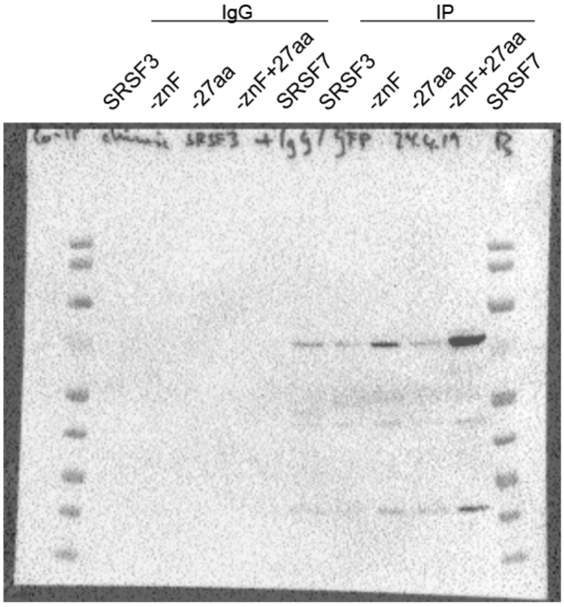

+RNase, IP: α-GFP, WB: α-CPSF6

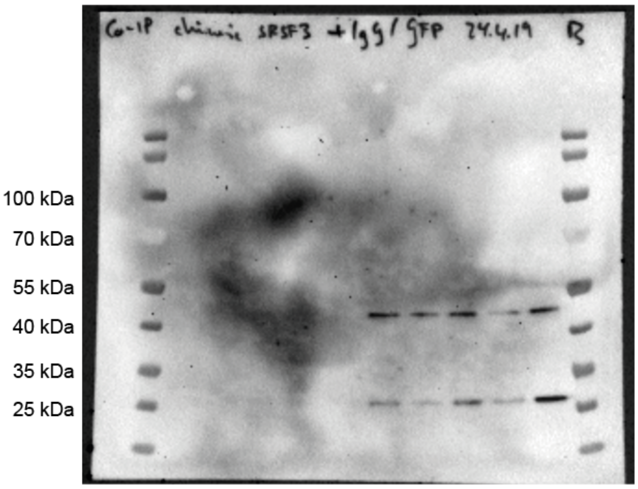

+RNase, IP: α-GFP, WB: α-CPSF5

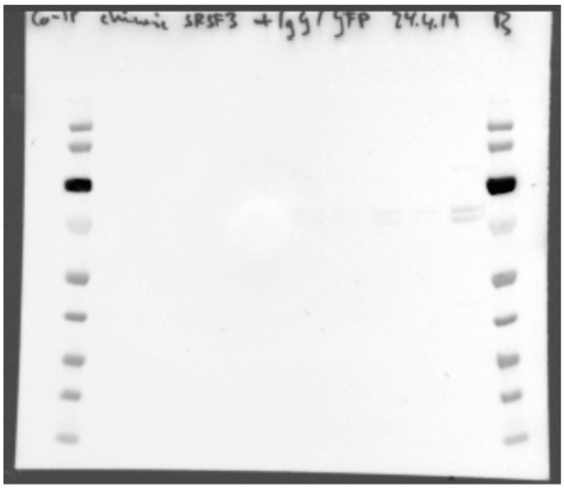
+RNase, IP: α-GFP, WB: α-PABPN1

source data Figure 4F

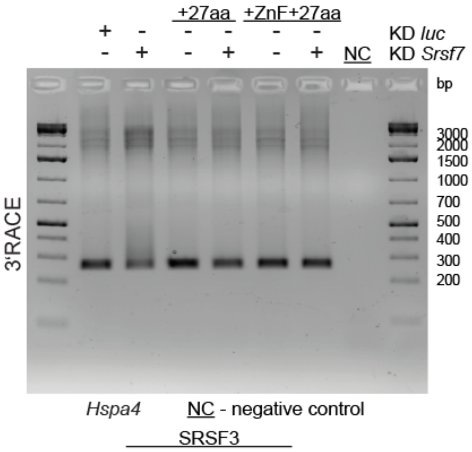

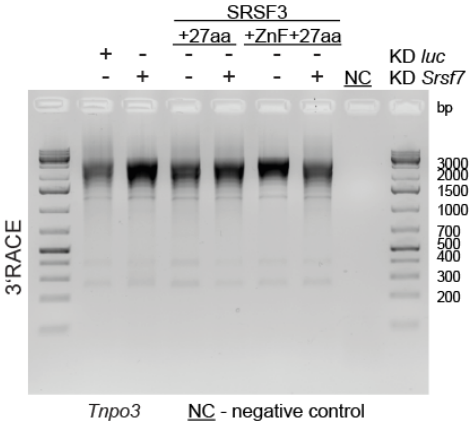

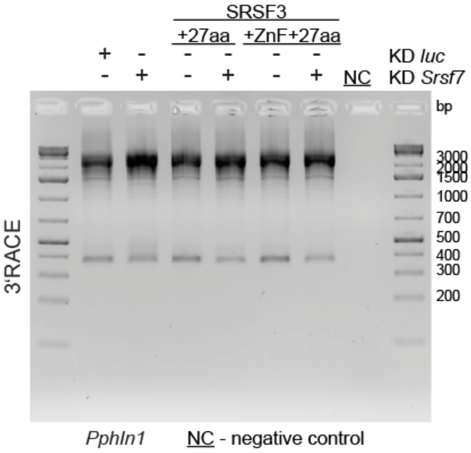

source data Figure 5B

UD Diff

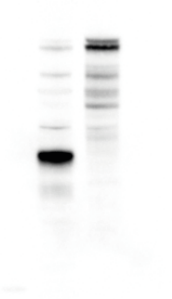

WB: α-OCT4

UD Diff

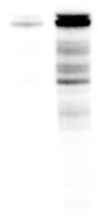

WB: α-Synapsin

UD Diff

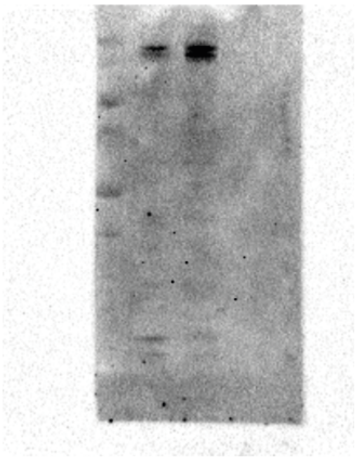

WB: α-Nestin

UD Diff

WB: α-CTNNB

UD Diff

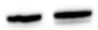

WB: α-CTNNB

UD Diff

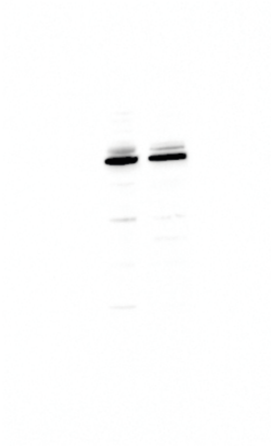

WB: α-CPSF6

UD Diff

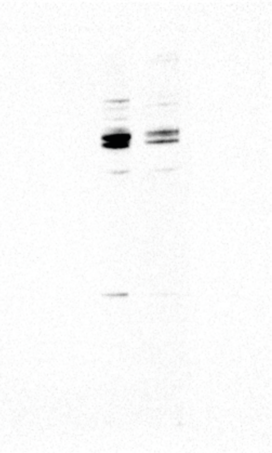

WB: α-FIP1

UD Diff

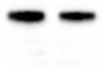

WB: α-SRSF3

UD Diff

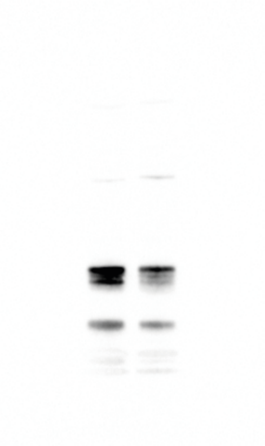

WB: α-SRSF7

source data Figure 6D

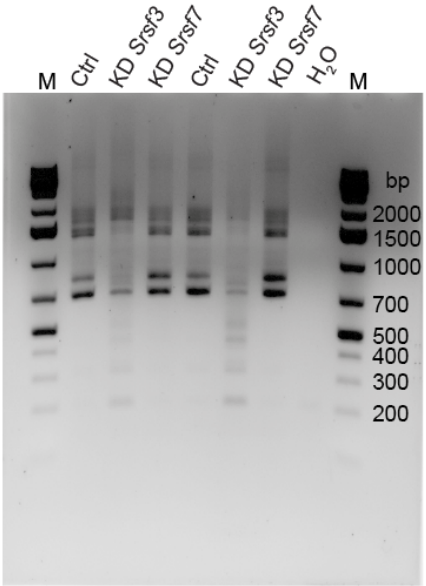

Primers Exon5 + Exon7

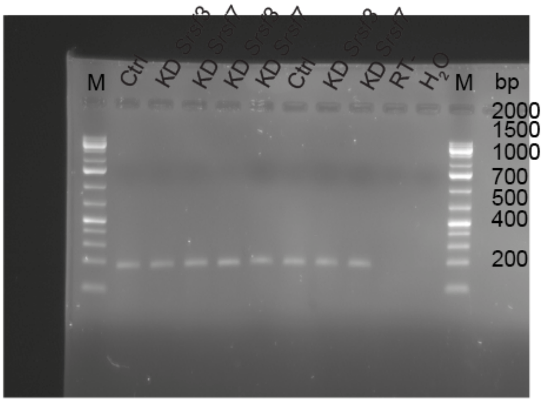

CalR

source data Figure 6E

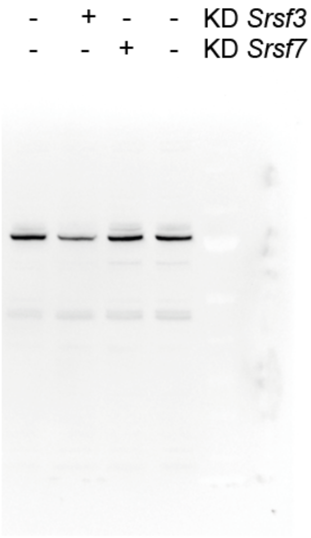

WB: α-CPSF6

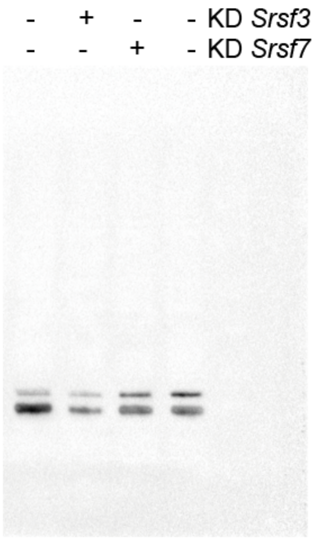

WB: α-CPSF5

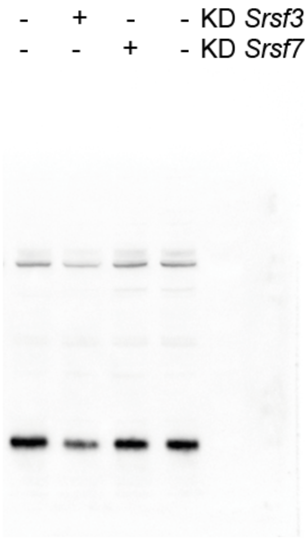

WB: α-SRSF3

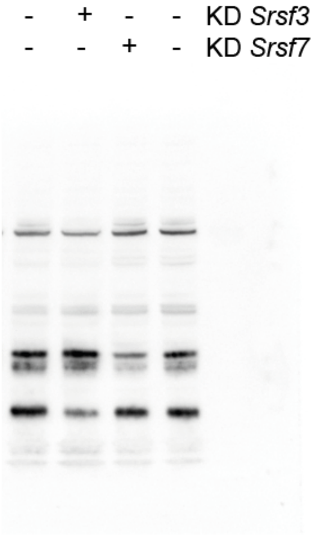

WB: α-SRSF7

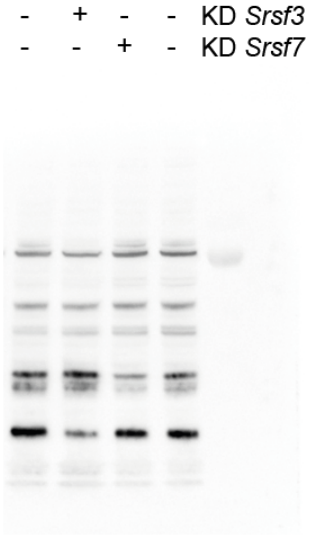

WB: α-αTUB
