# Supplementary material for: SRSF3 and SRSF7 modulate 3′UTR length through suppression or activation of proximal polyadenylation sites and regulation of CFIm levels
Source: Genome Biol. 2021 Mar 11;22:82. doi: 10.1186/s13059-021-02298-y (PMC7948361; doi:10.1186/s13059-021-02298-y)

source data Figure S1A

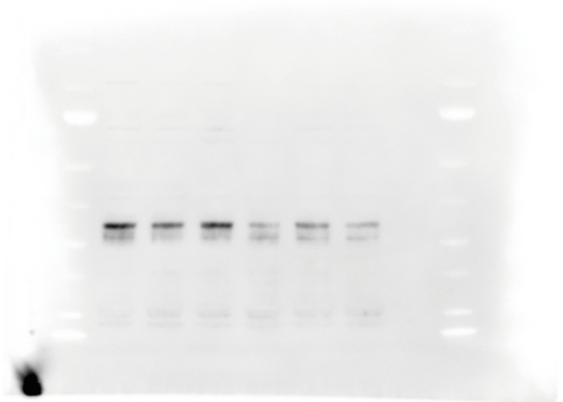

a-SRSF7

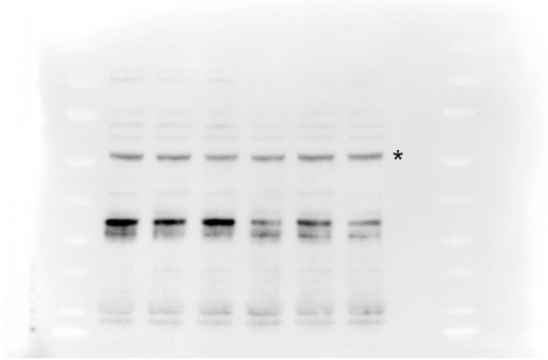

a-TUB

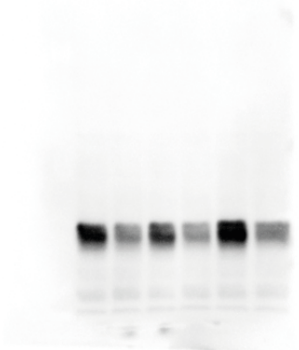

a-SRSF3

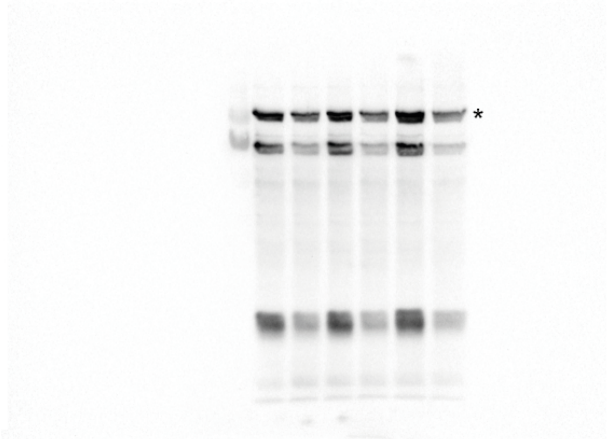

a-CTTNB

source data Figure S1D

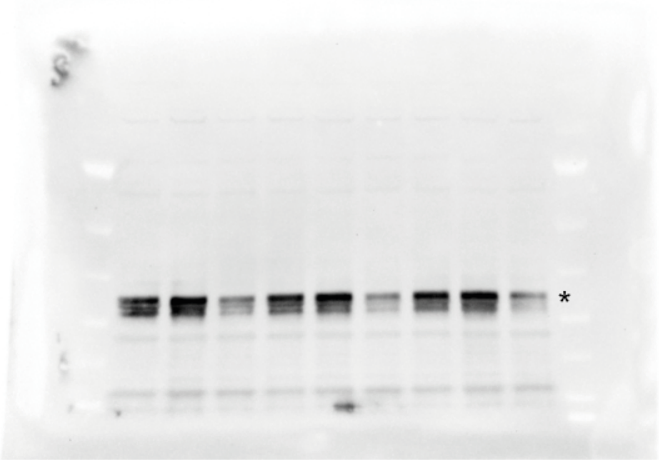

a-SRSF7

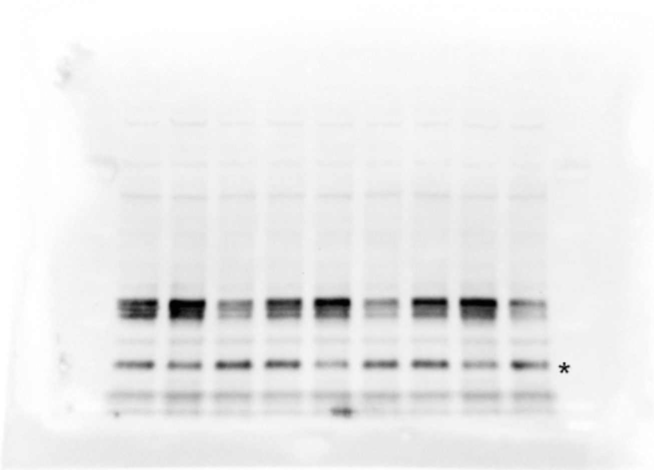

a-SRSF3

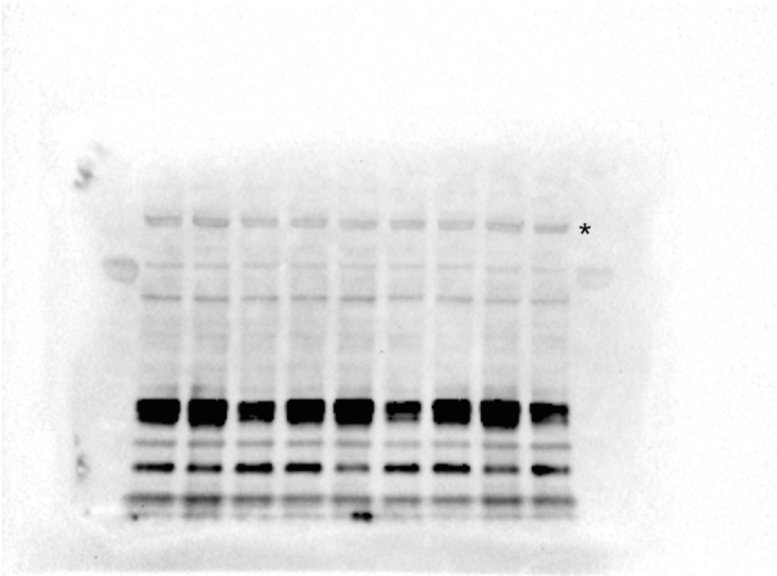

a-CTNNB

source data Figure S3A & S3B

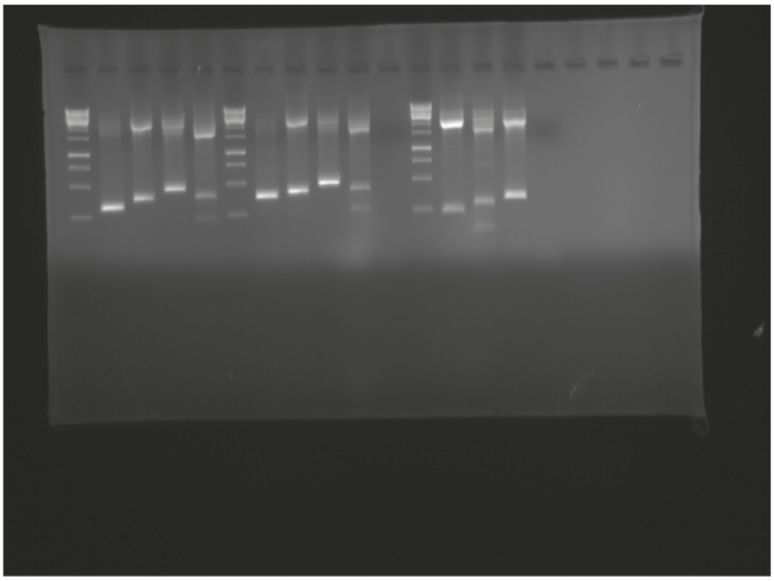

source data Figure S3C

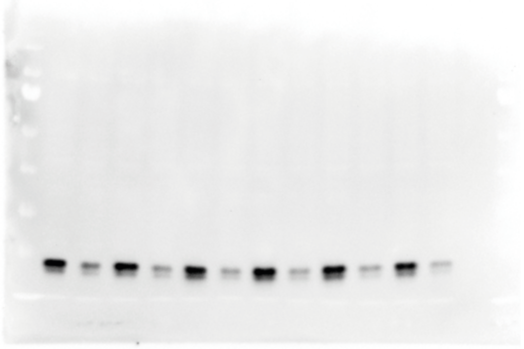

a-SRSF3

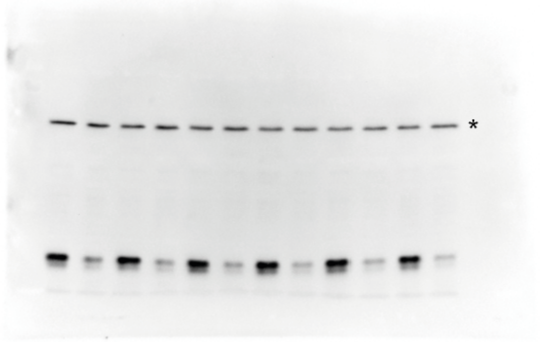

a-TUB

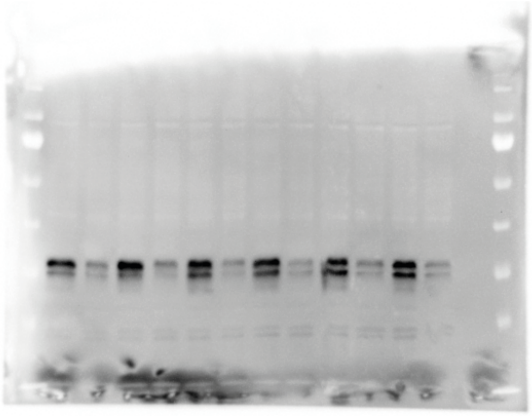

a-SRSF7

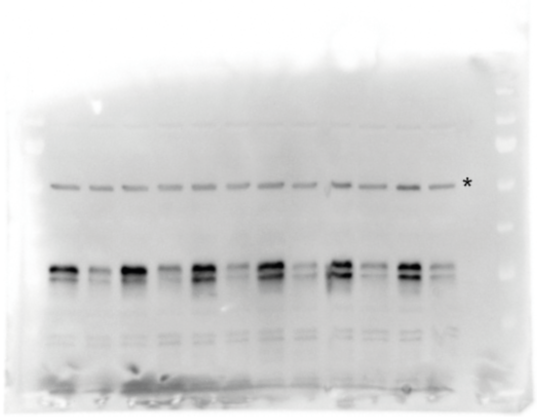

a-TUB

*

source data Figure S3D

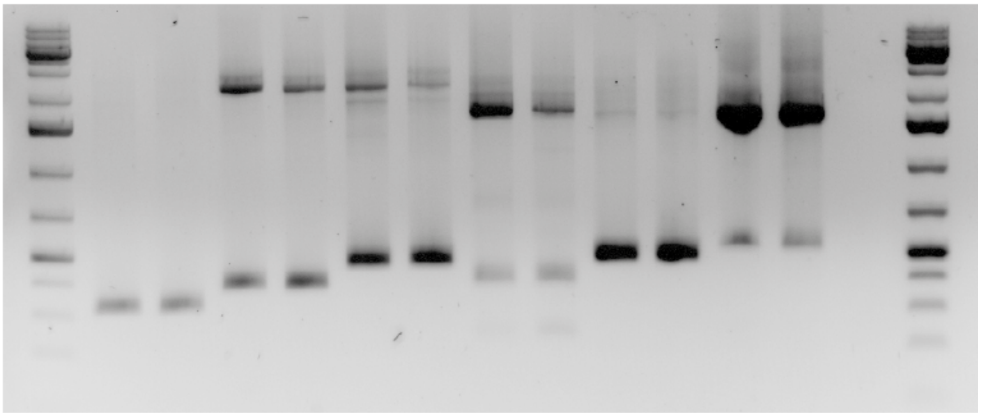

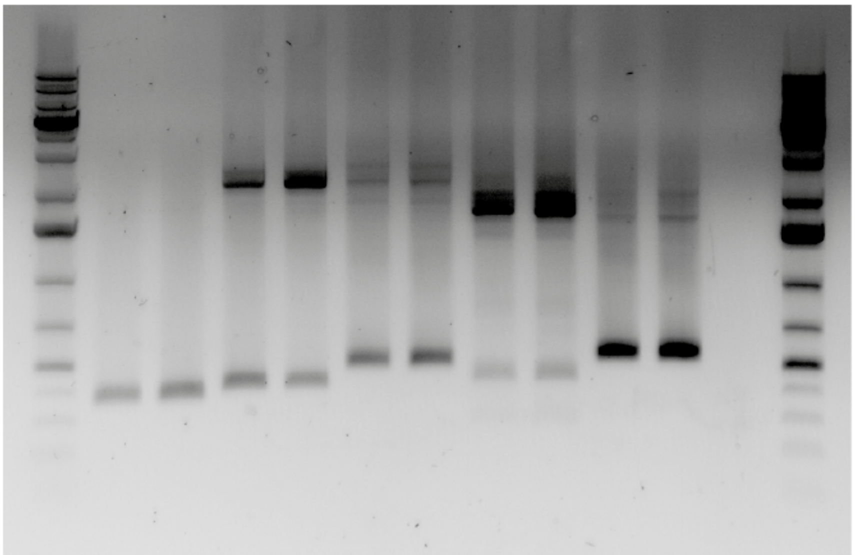

source data Figure S4E

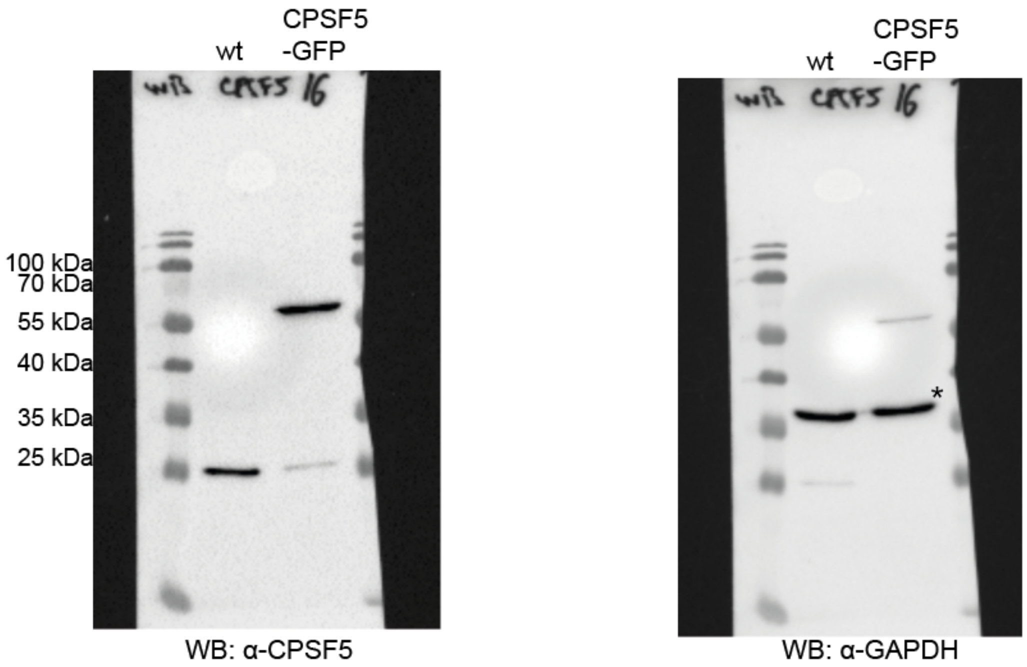

source data Figure S4F

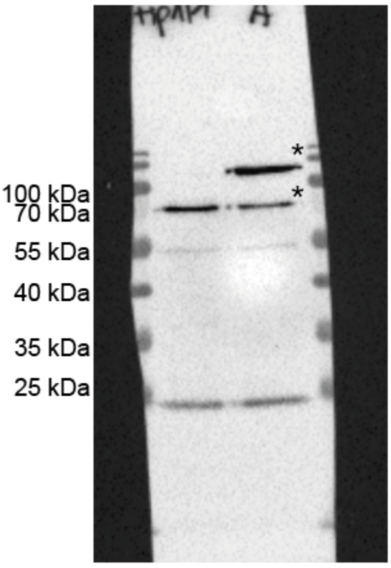

WB: α-CPSF6

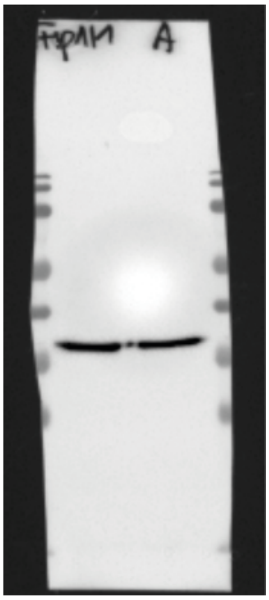

WB: α-GAPDH

source data Figure S4G

wt GFP-FIP1

100 kDa
70 kDa
55 kDa
40 kDa
35 kDa
25 kDa

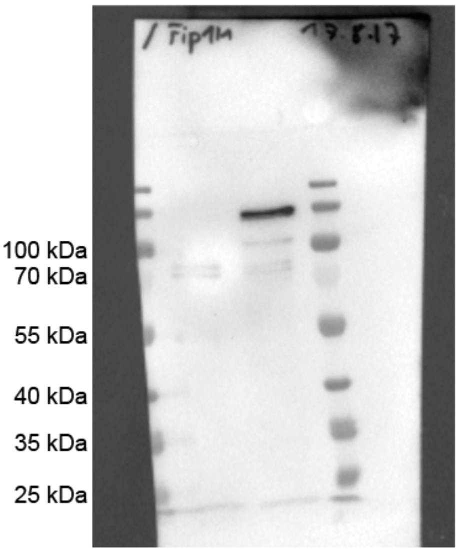

WB: α-FIP1

wt GFP-FIP1

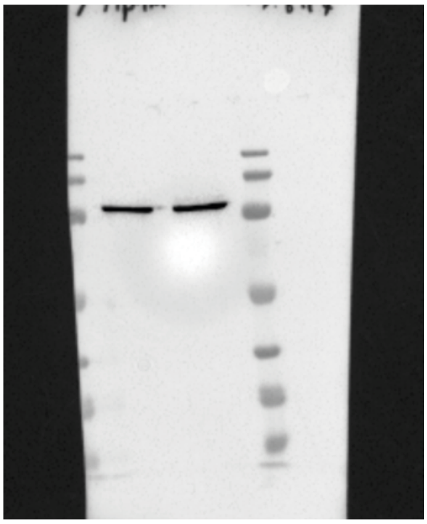

WB: α-CTNNB

source data Figure S4H

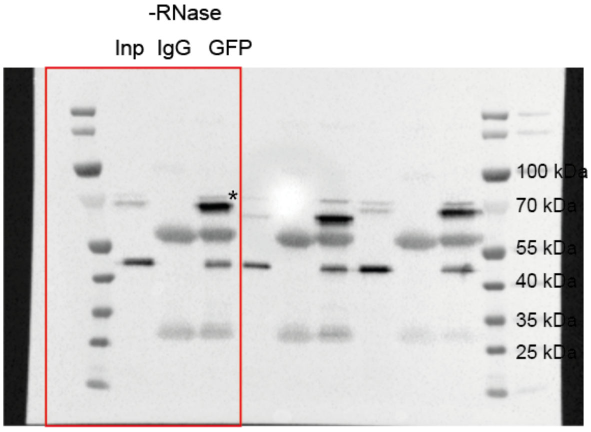

IP: SRSF7-GFP, WB: α-GFP, -RNase

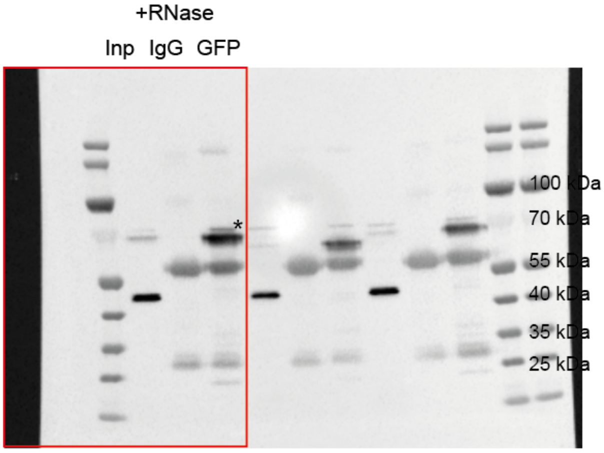

IP: SRSF7-GFP, WB: α-GFP, +RNase

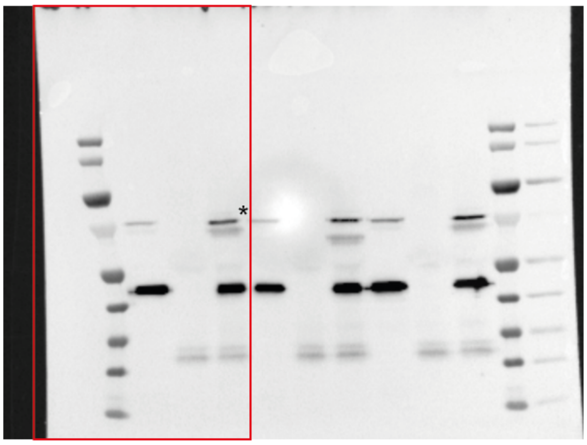

IP: SRSF7-GFP, WB: α-CPSF6, -RNase

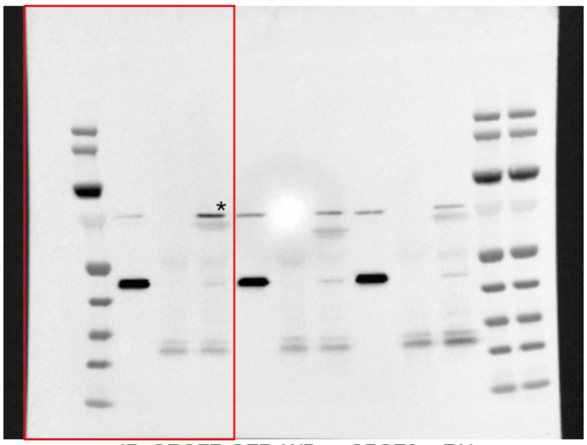

IP: SRSF7-GFP, WB: α-CPSF6, +RNase

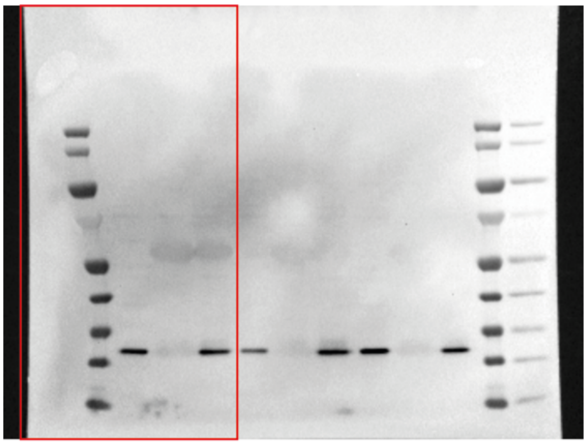

IP: SRSF7-GFP, WB: α-CPSF5, -RNase

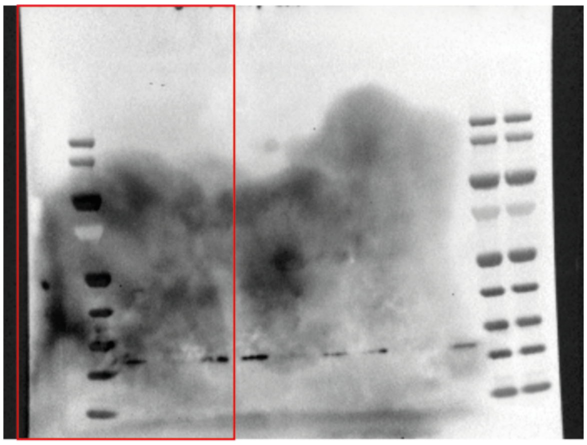

IP: SRSF7-GFP, WB: α-CPSF5, +RNase

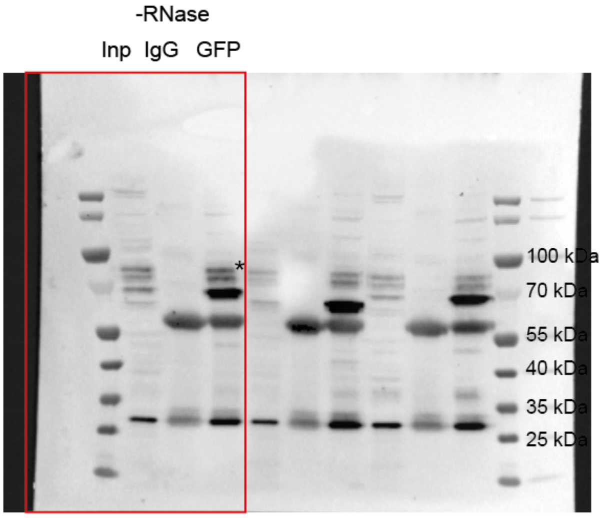

IP: SRSF7-GFP, WB: α-FIP1, -RNase

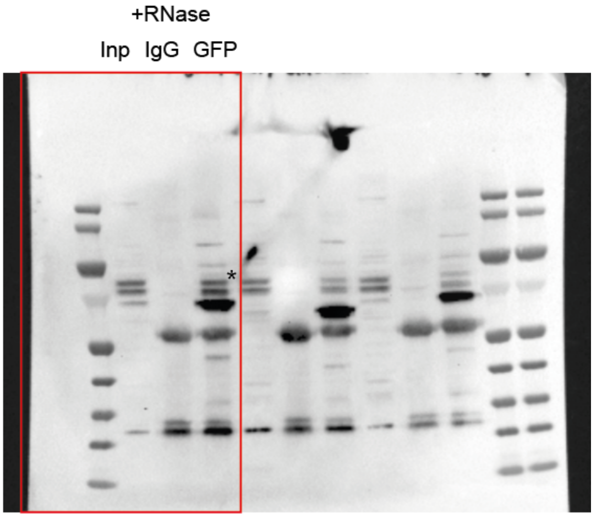

IP: SRSF7-GFP, WB: α-FIP1, +RNase

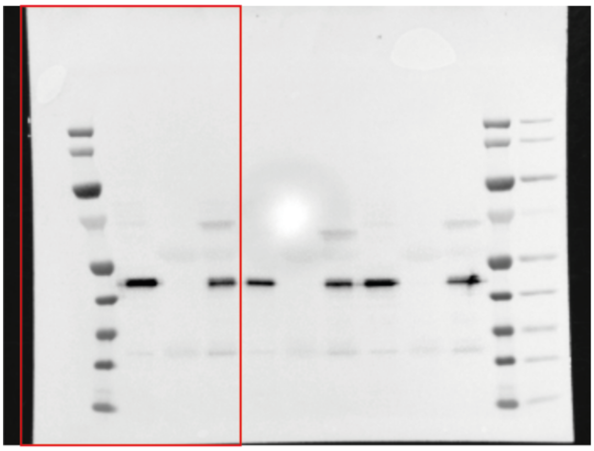

IP: SRSF7-GFP, WB: α-PABPN1, -RNase

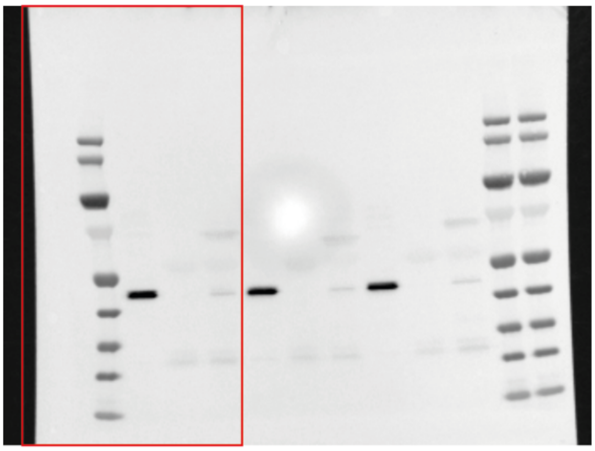

IP: SRSF7-GFP, WB: α-PABPN1, +RNase

source data Figure S4I

-RNase +RNase

Inp IgG GFP Inp IgG GFP

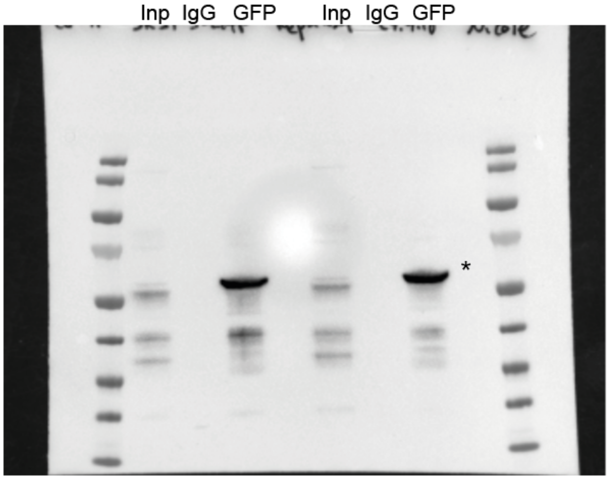

IP: SRSF3-GFP, WB: α-GFP

-RNase +RNase

Inp IgG GFP Inp IgG GFP

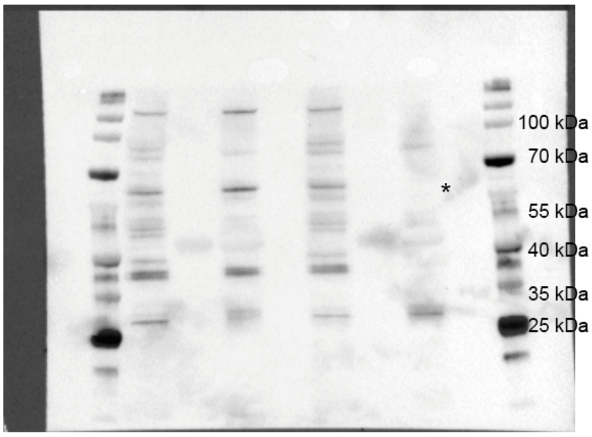

IP: SRSF3-GFP, WB: α-CPSF6

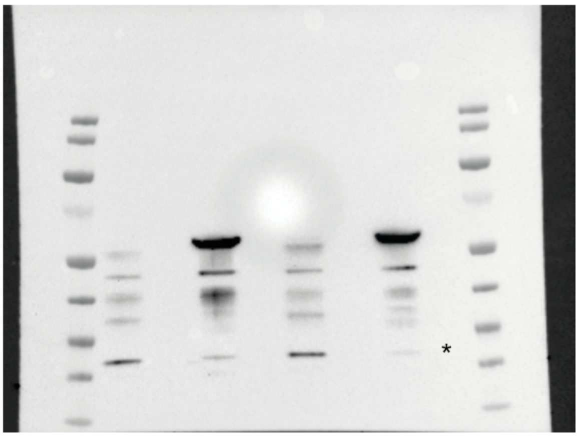

IP: SRSF3-GFP, WB: α-CPSF5

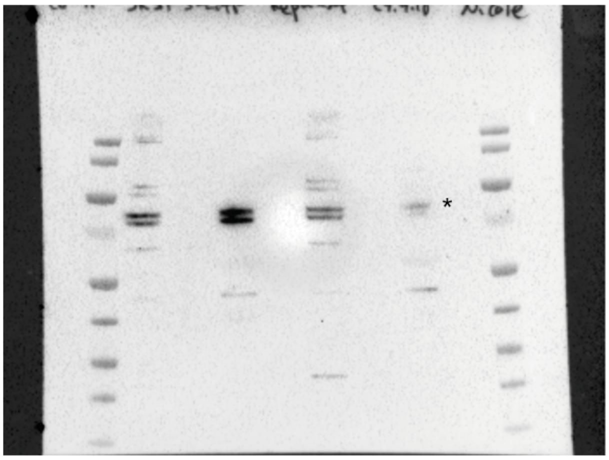

IP: SRSF3-GFP, WB: α-FIP1

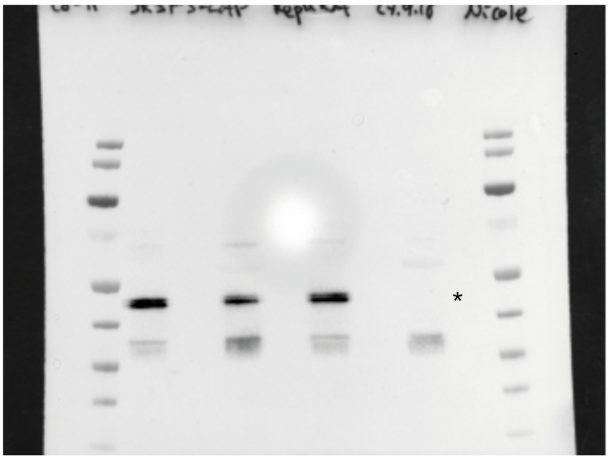

IP: SRSF3-GFP, WB: α-PABPN1

source data Figure S5A

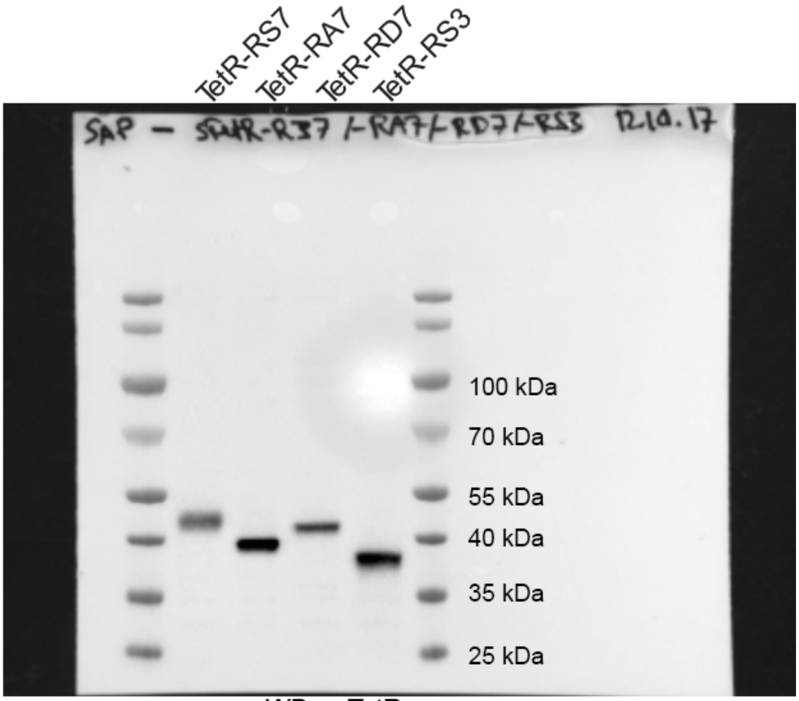

WB: α-TetR

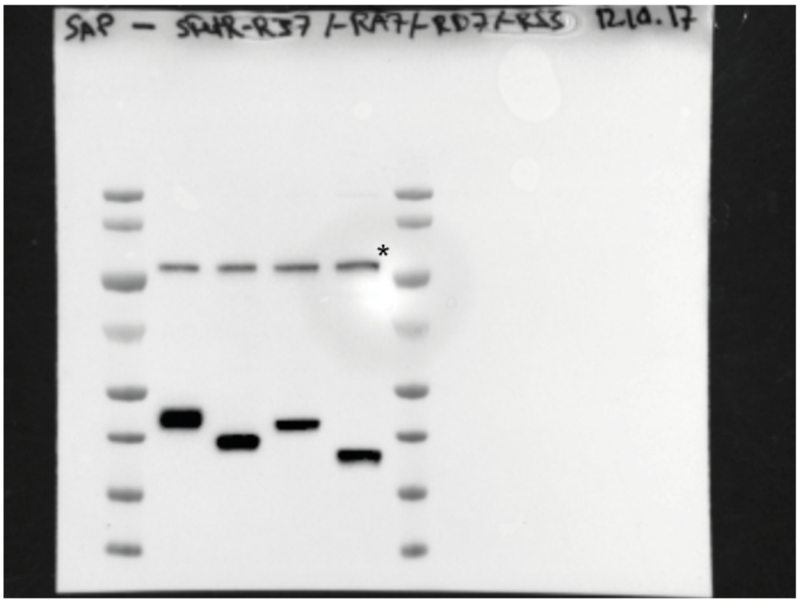

WB: α-CTNNB

source data Figure S5B

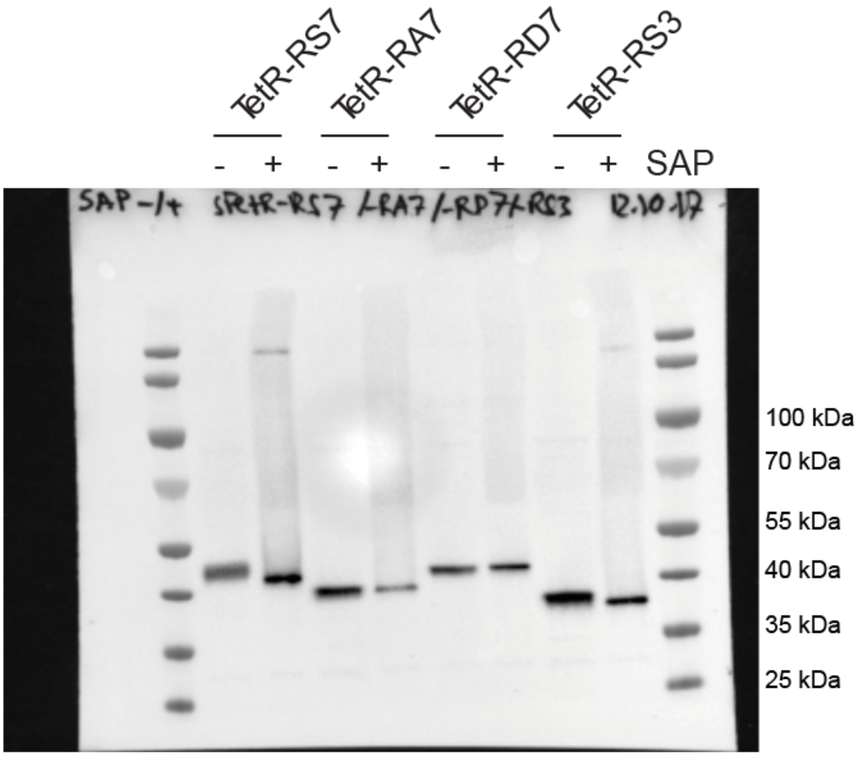

WB: α-TetR

source data Figure S5C

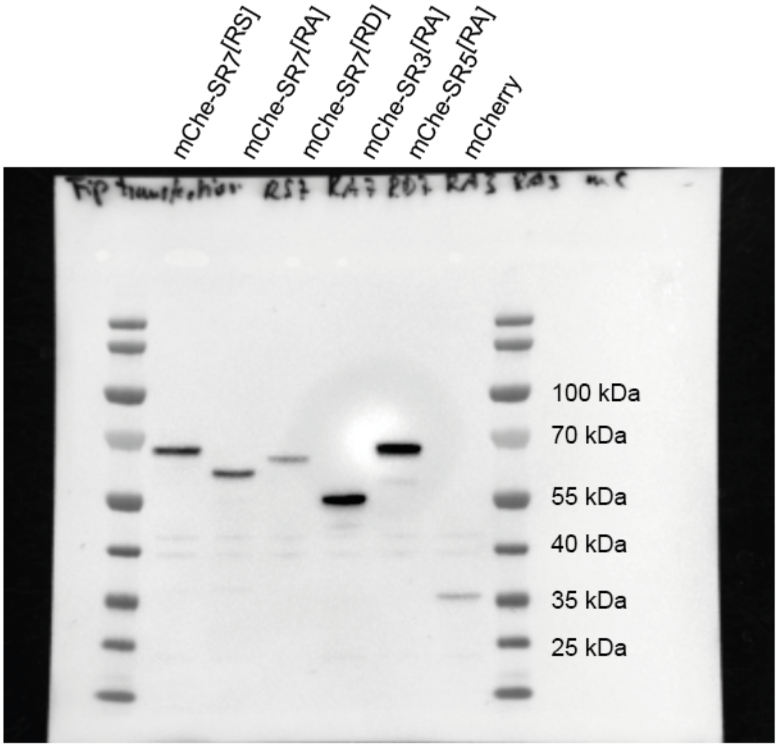

WB: α-mCherry

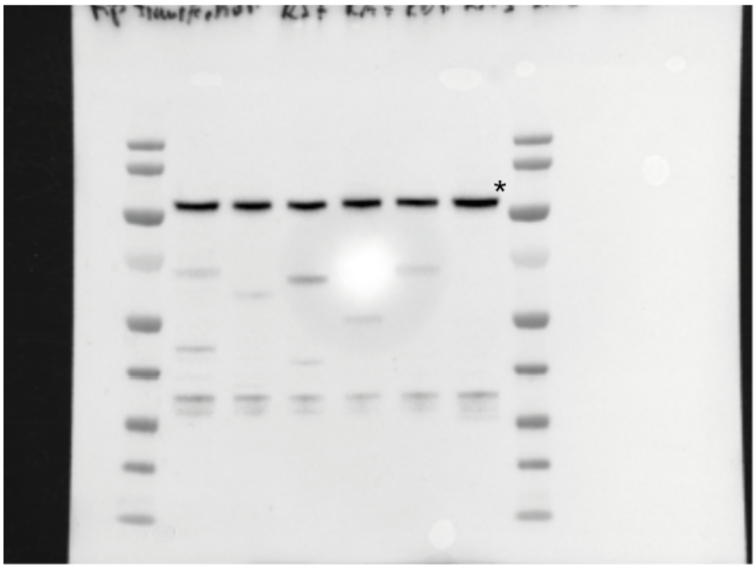

WB: α-CTNNB

source data Figure S5E

Ctrl KD

100 kDa
70 kDa
55 kDa
40 kDa
35 kDa
25 kDa

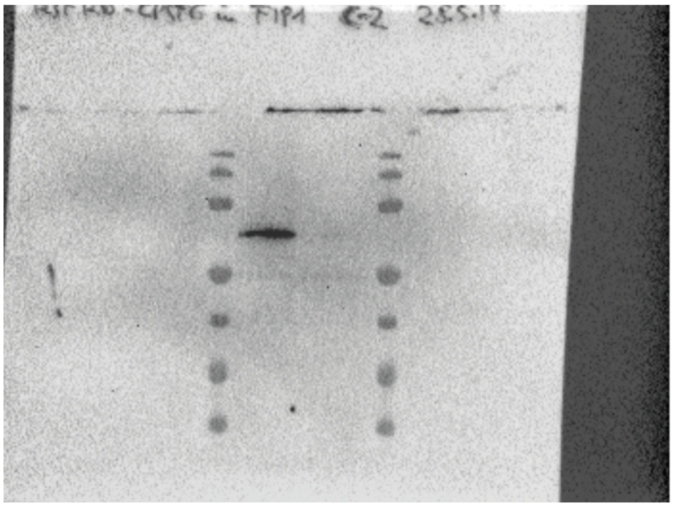

GFP-FIP1, WB: α-CPSF6

Ctrl KD

100 kDa
70 kDa
55 kDa
40 kDa
35 kDa
25 kDa

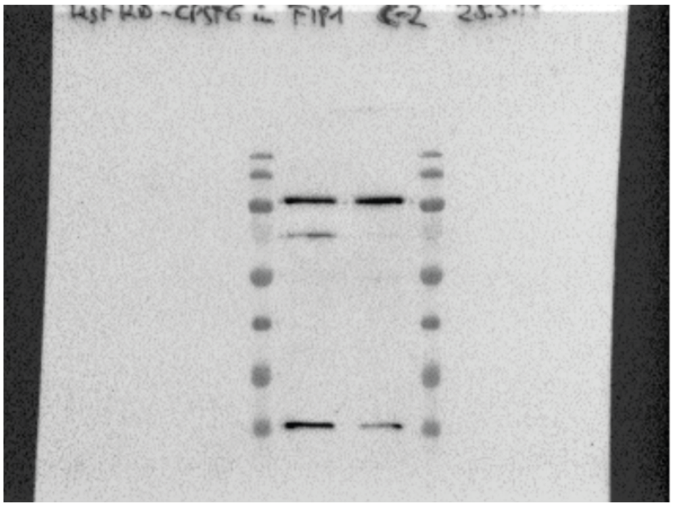

GFP-FIP1, WB: α-CPSF5

Ctrl KD

100 kDa
70 kDa
55 kDa
40 kDa
35 kDa
25 kDa

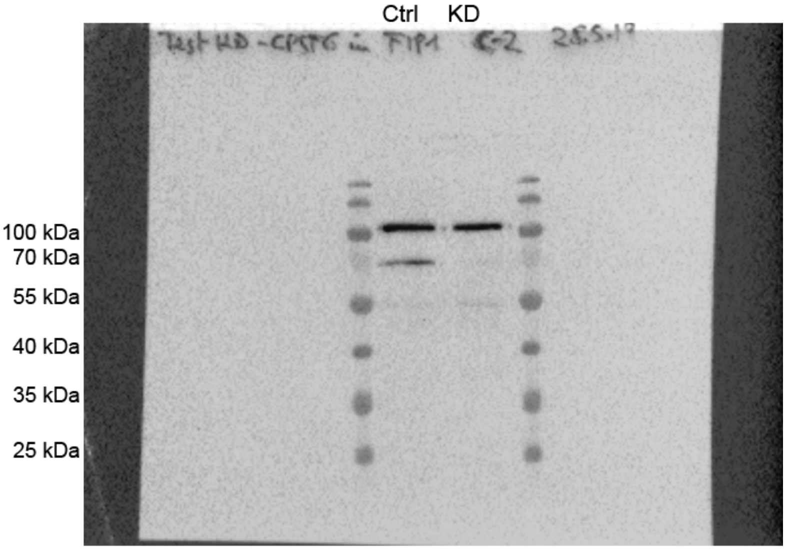

GFP-FIP1, WB: α-CTNNB

source data Figure S5F

Ctrl KD *Cpsf6*

Inp IgG IP Inp IgG IP

100 kDa
70 kDa
55 kDa
40 kDa
35 kDa
25 kDa

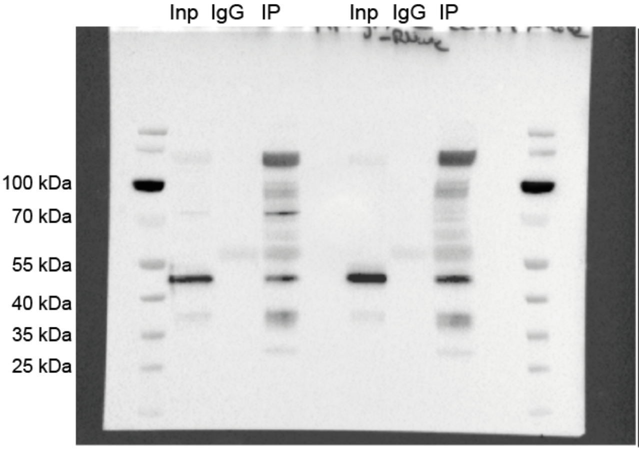

IP: GFP-FIP1, WB: α-GFP, -RNase

Ctrl KD *Cpsf6*

Inp IgG IP Inp IgG IP

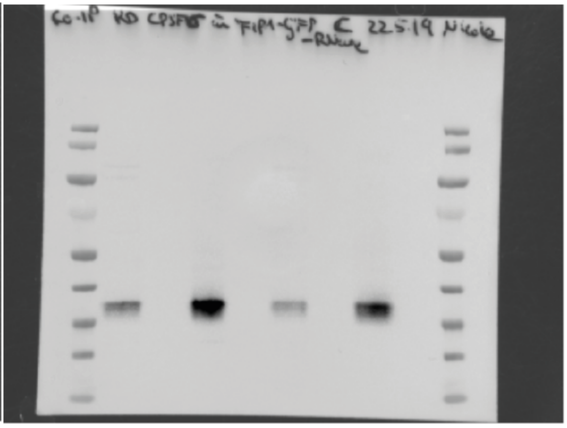

IP: GFP-FIP1, WB: α-SRSF7, -RNase

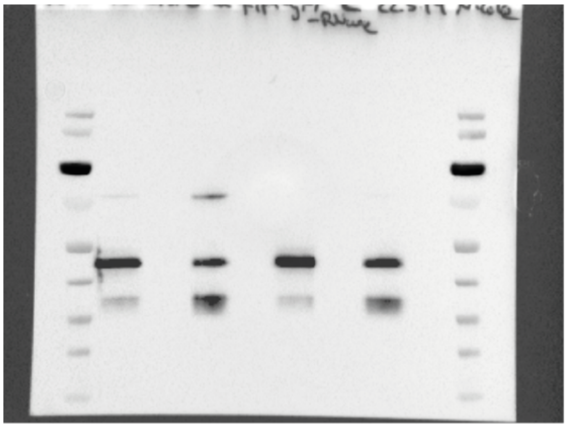

IP: GFP-FIP1, WB: α-CPSF6, -RNase

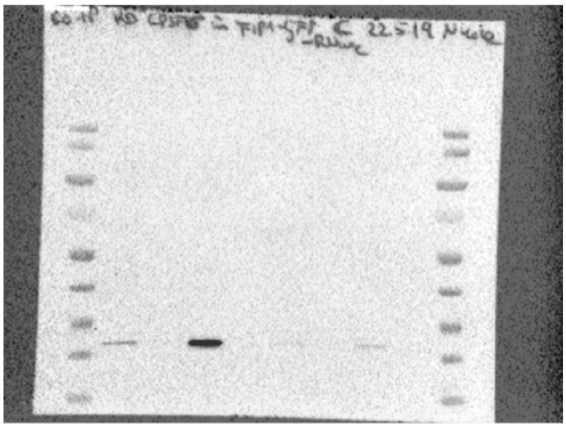

IP: GFP-FIP1, WB: α-CPSF5, -RNase

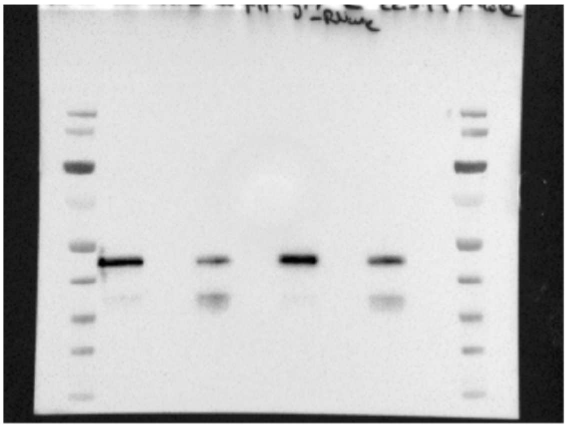

IP: GFP-FIP1, WB: α-PABPN1, -RNase

source data Figure S5G

Ctrl KD *Cpsf6*
Inp IgG IP Inp IgG IP

100 kDa
70 kDa
55 kDa
40 kDa
35 kDa
25 kDa

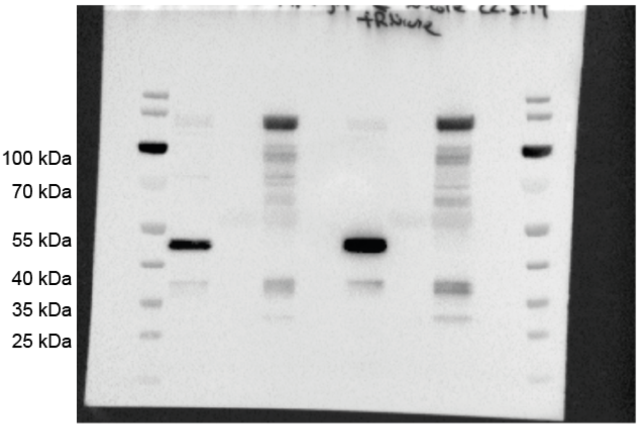

IP: GFP-FIP1, WB: α-GFP, +RNase

Ctrl KD *Cpsf6*
Inp IgG IP Inp IgG IP

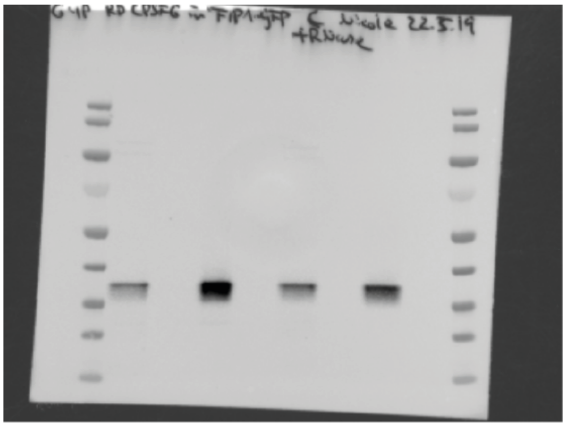

IP: GFP-FIP1, WB: α-SRSF7, +RNase

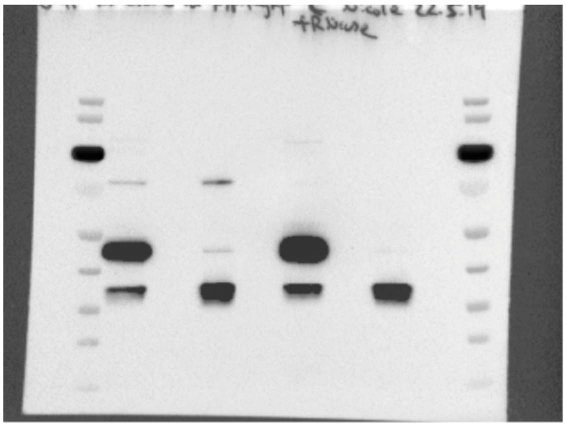

IP: GFP-FIP1, WB: α-CPSF6, +RNase

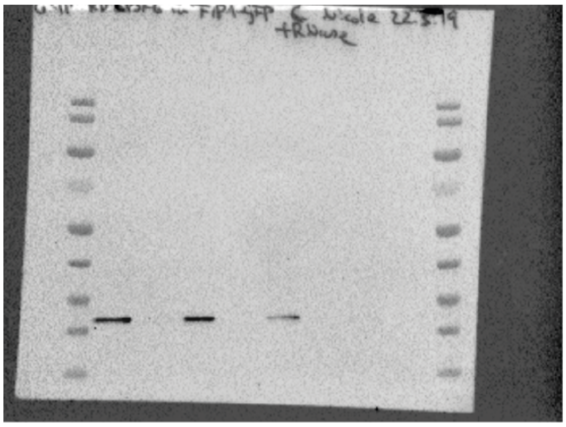

IP: GFP-FIP1, WB: α-CPSF5, +RNase

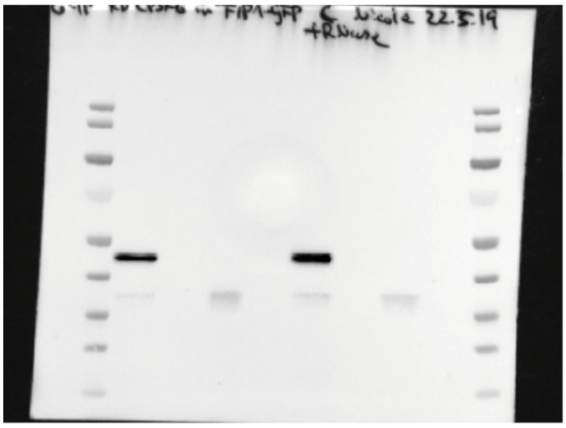

IP: GFP-FIP1, WB: α-PABPN1, +RNase

source data Figure S6B

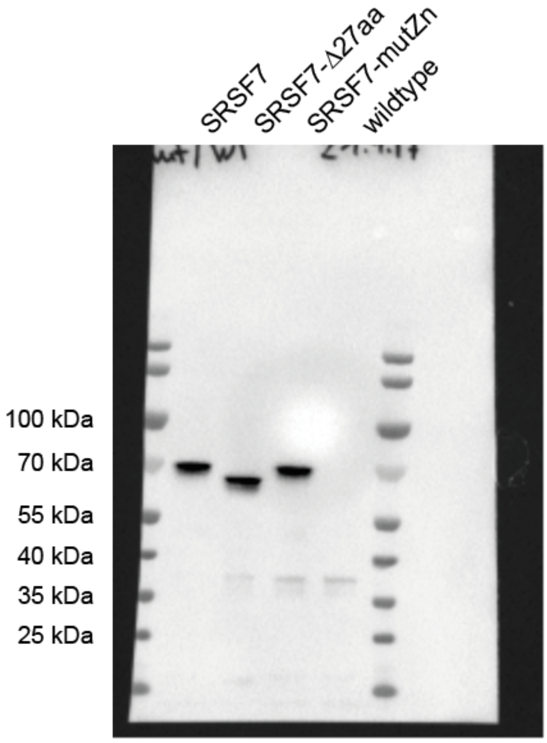

WB: α-SRSF7

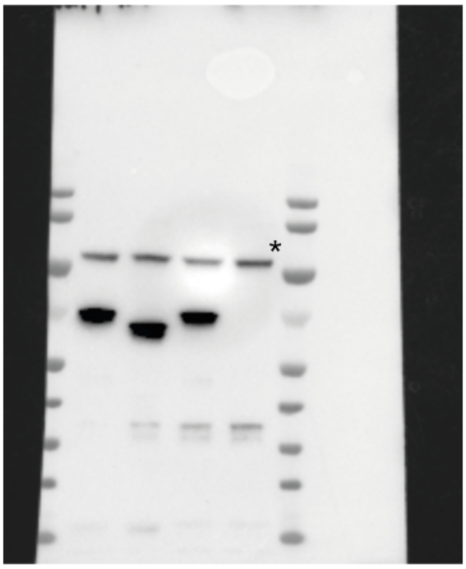

WB: α-CTNNB

source data Figure S6D

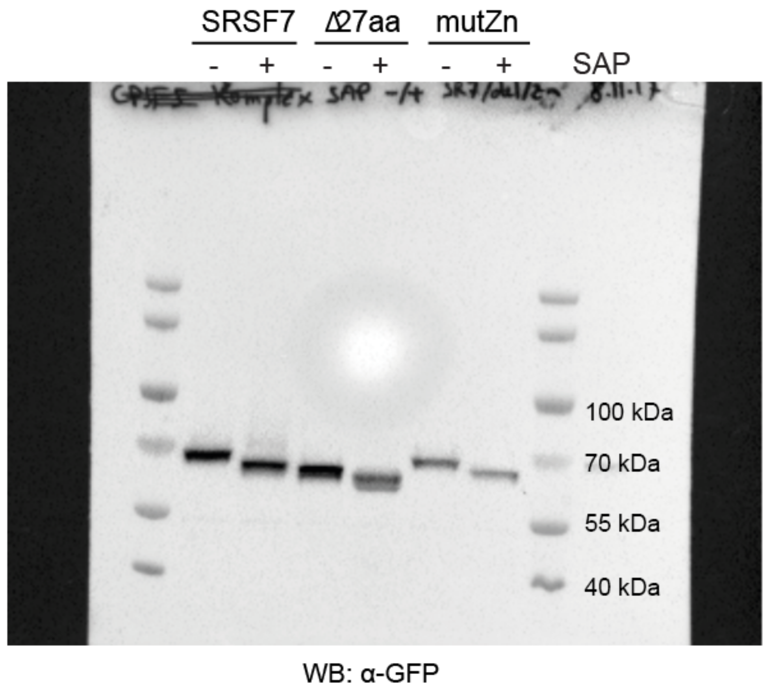

source data Figure S6E

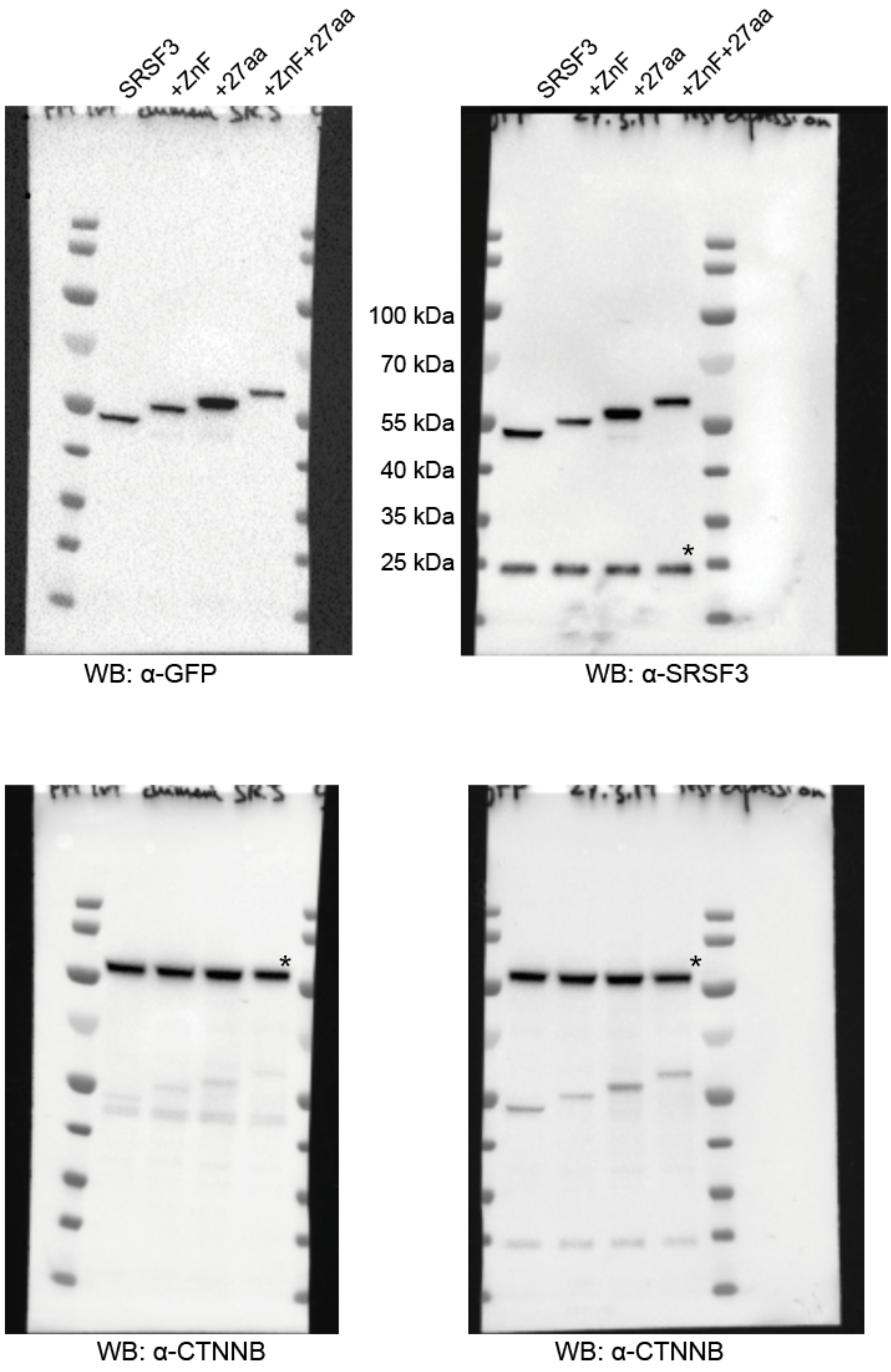

source data Figure S6F

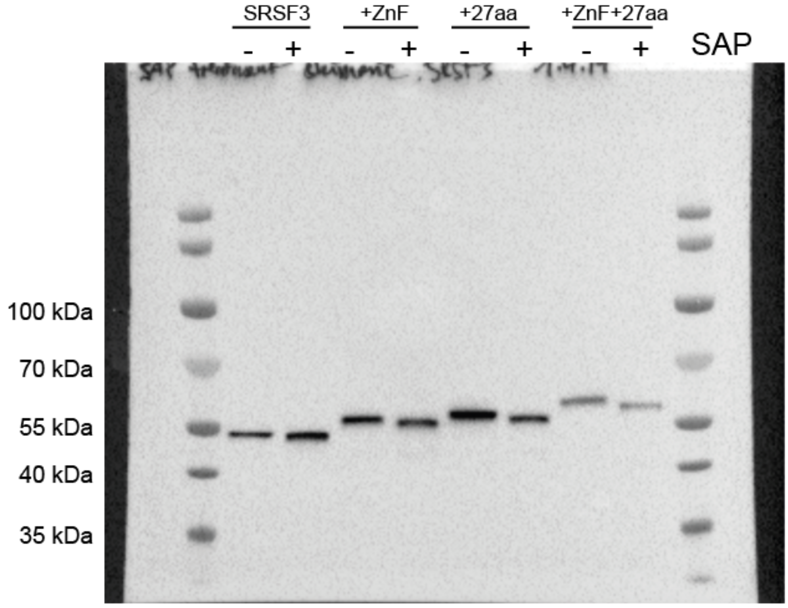

WB: α-GFP

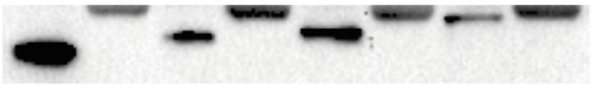

WB: α-mAb104

source data Figure S7A

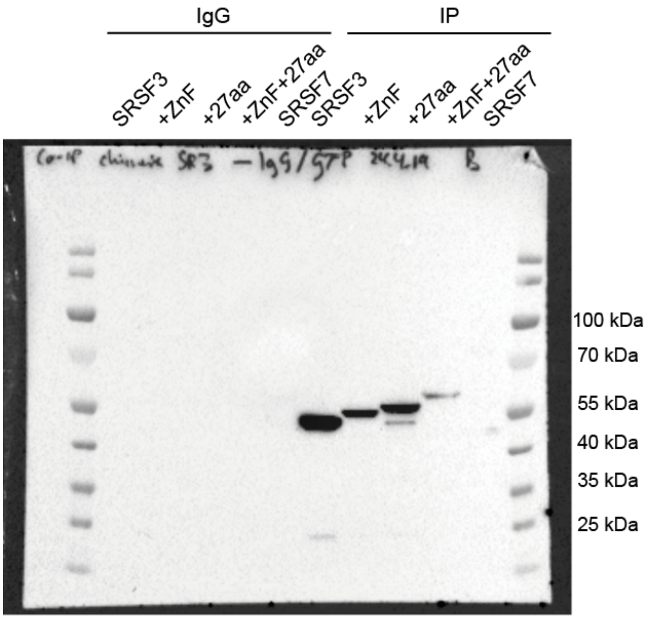

IP: α-GFP, WB: α-SRSF3, -RNase

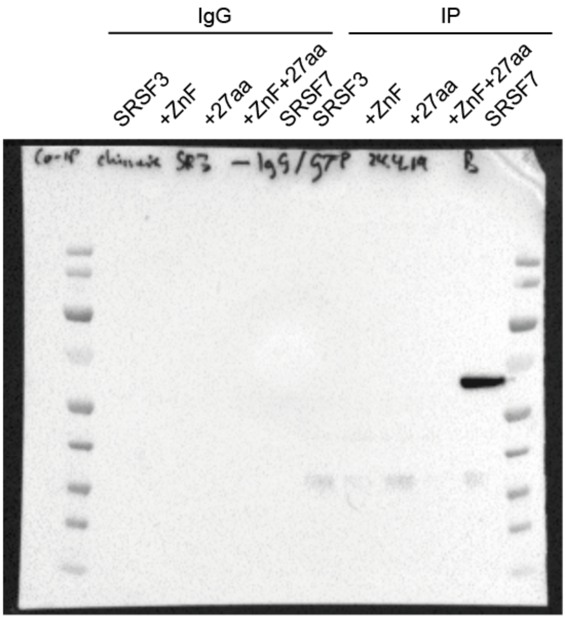

IP: α-GFP, WB: α-SRSF7, -RNase

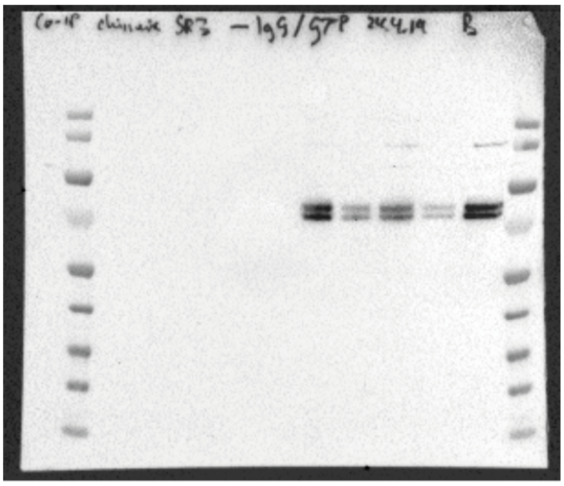

IP: α-GFP, WB: α-FIP1, -RNase

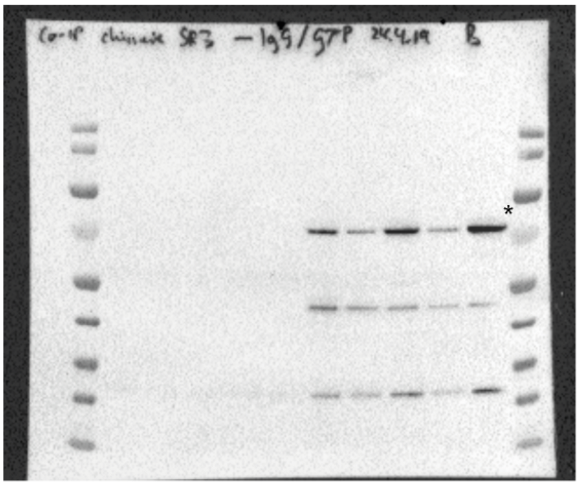

IP: α-GFP, WB: α-CPSF6, -RNase

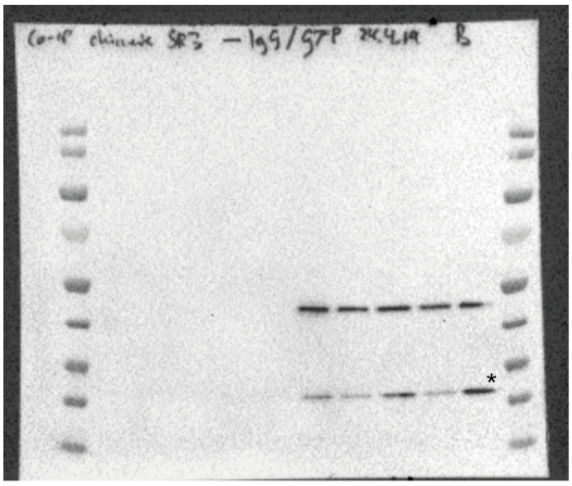

IP: α-GFP, WB: α-CPSF5, -RNase

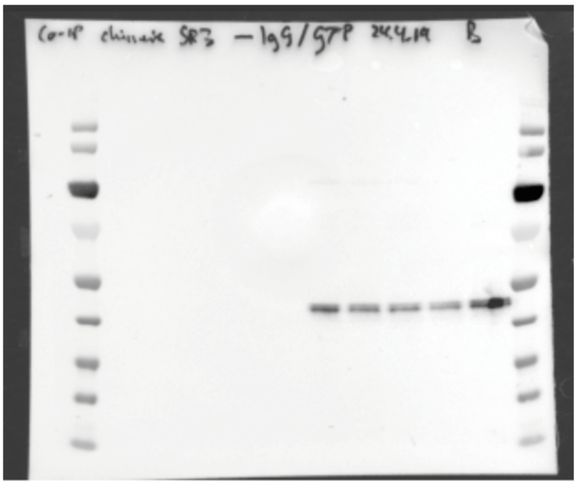

IP: α-GFP, WB: α-PABPN1, -RNase

source data Figure S7B

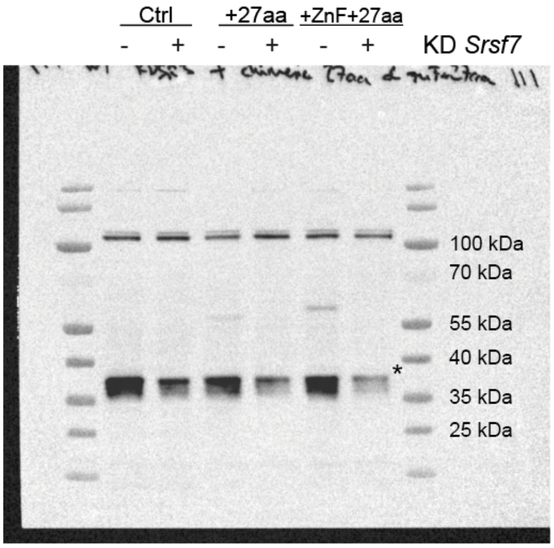

WB: α-SRSF7

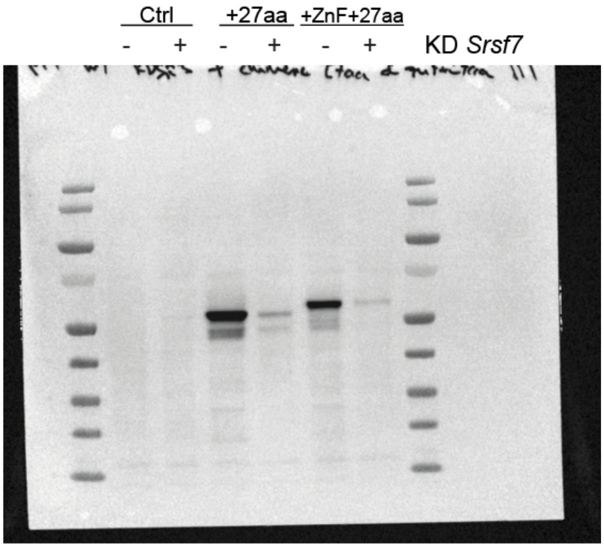

WB: α-GFP

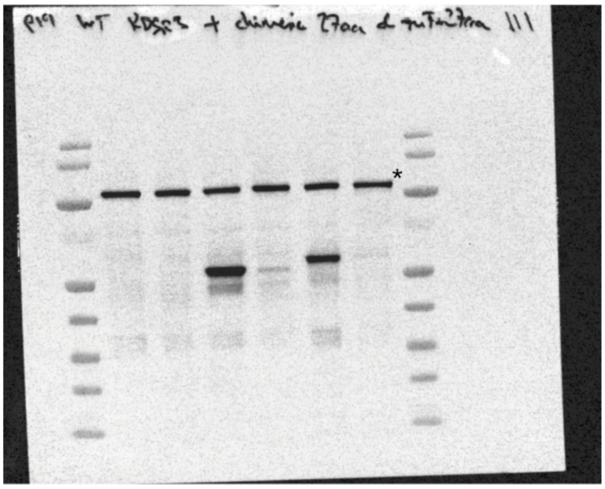

WB: α-CTNNB

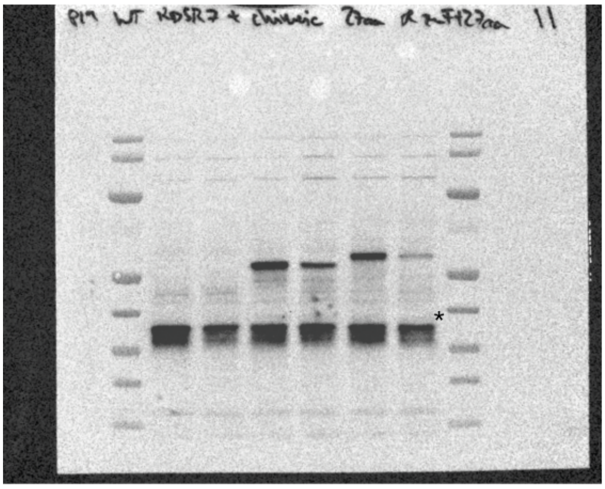

WB: α-SRSF7

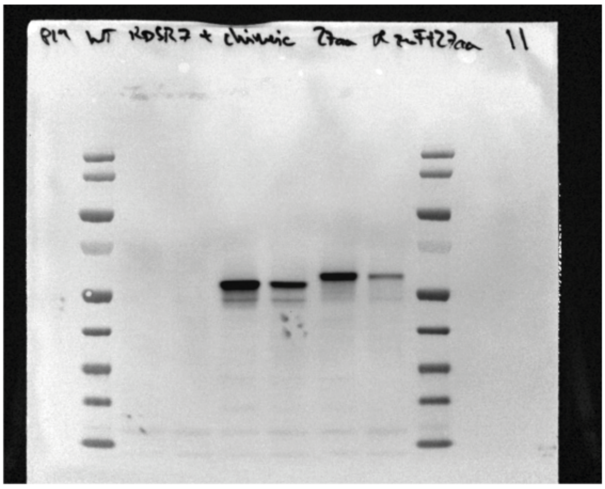

WB: α-GFP

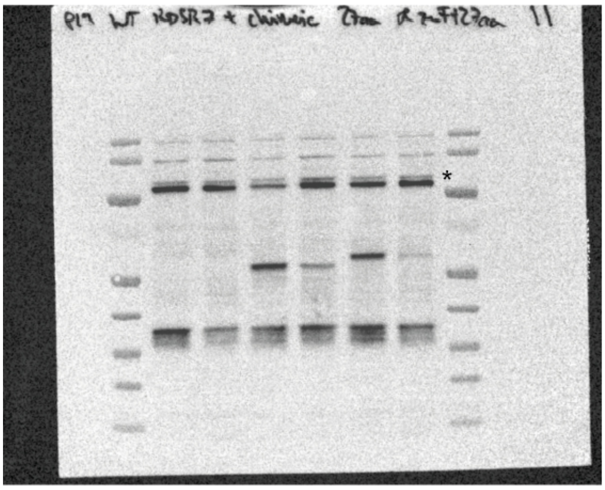

WB: α-CTNNB

source data Figure S8E

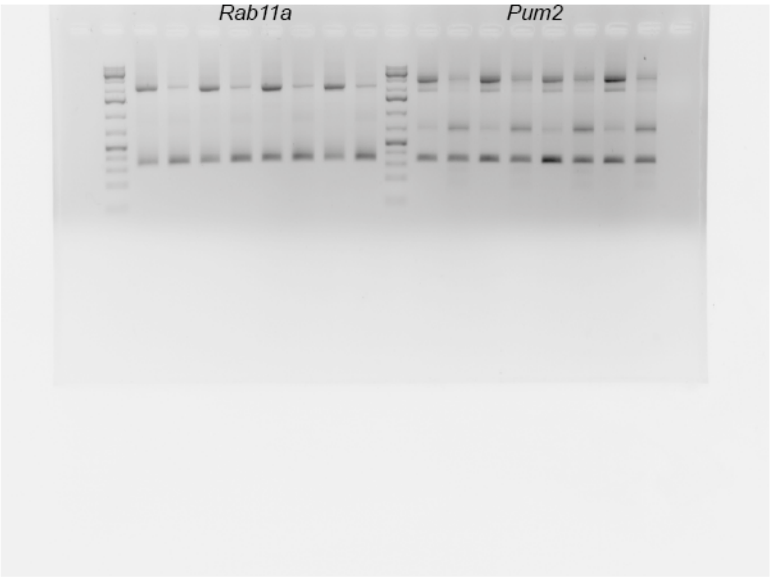

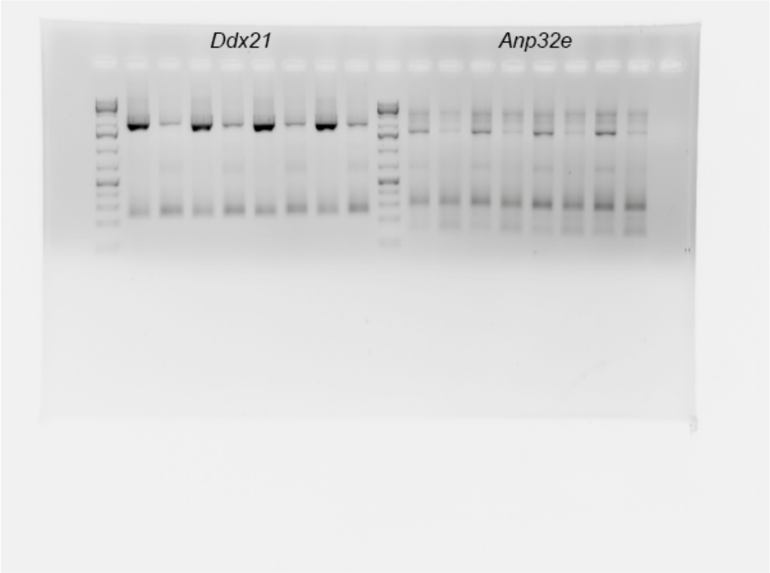

source data Figure S8F

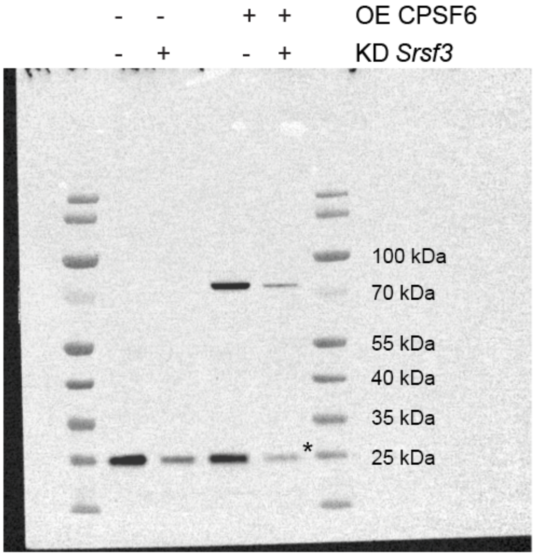

WB: α-SRSF3

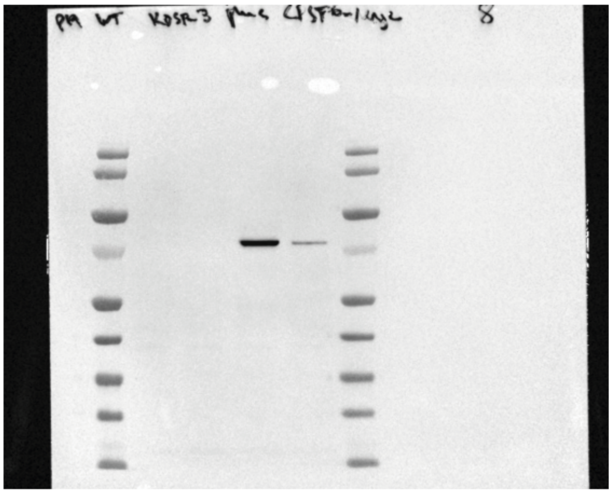

WB: α-Myc

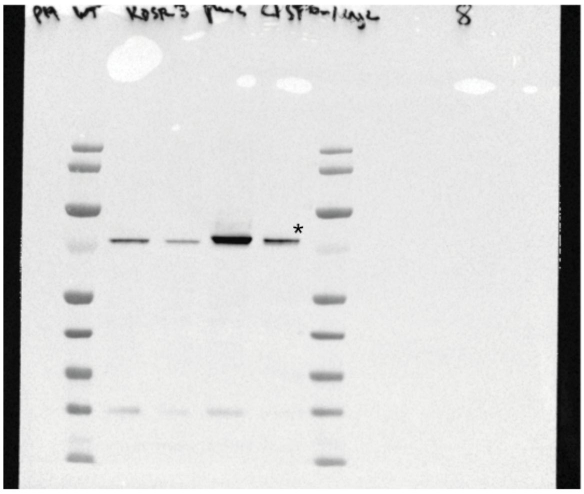

WB: α-CPSF6

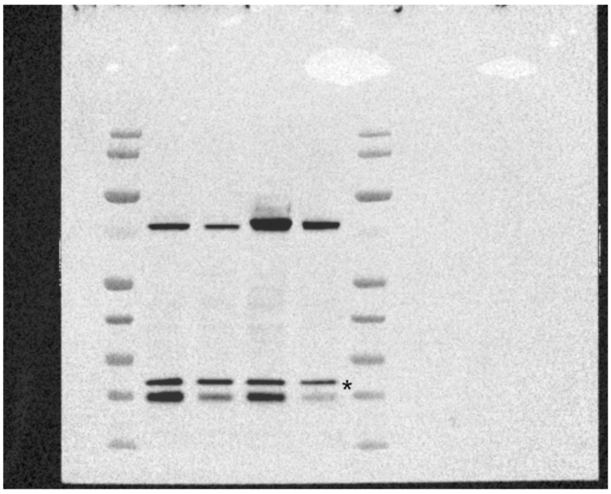

WB: α-CPSF5

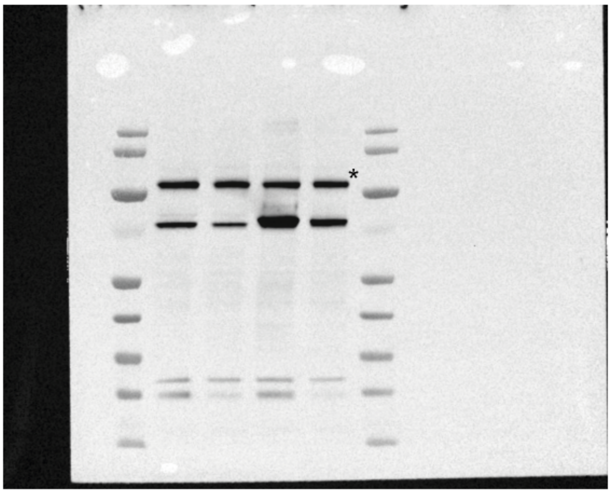

WB: α-CTNNB

source data Figure S8G

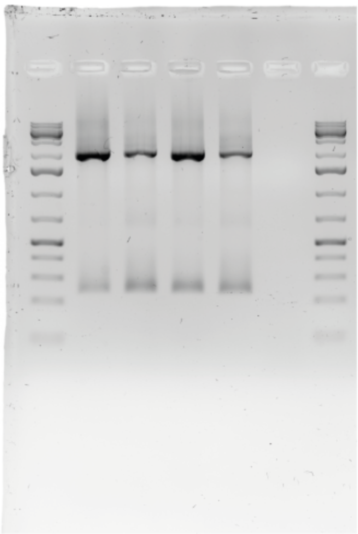

Ddx21

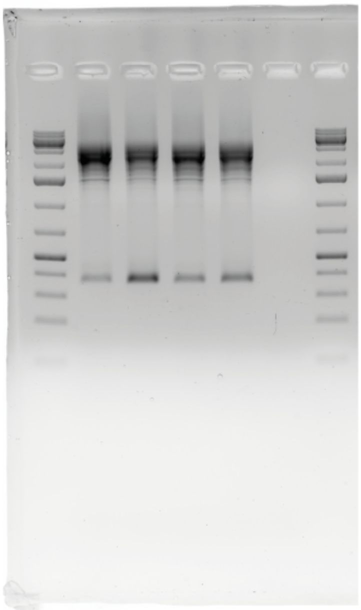

Pphln1

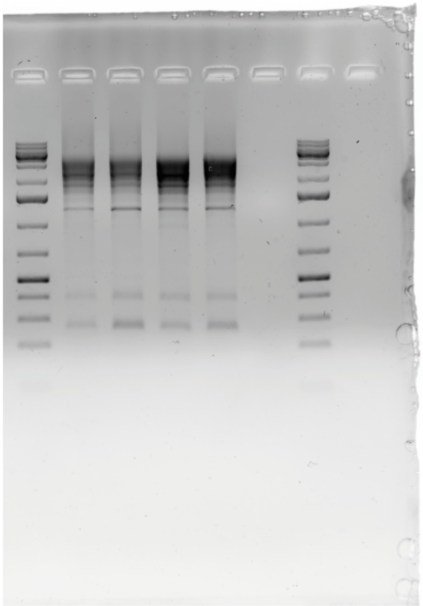

Tnpo3

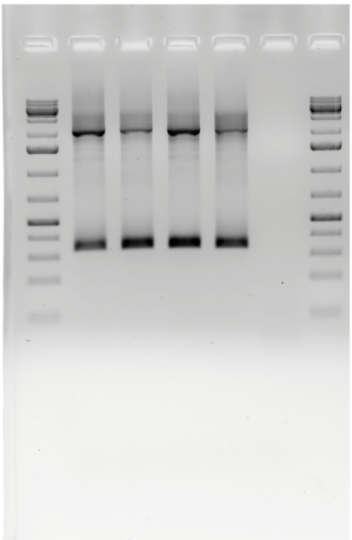

Anp32e

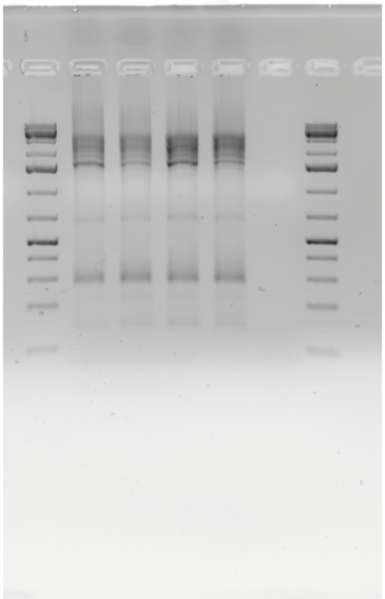

Rab11a

source data Figure S10A

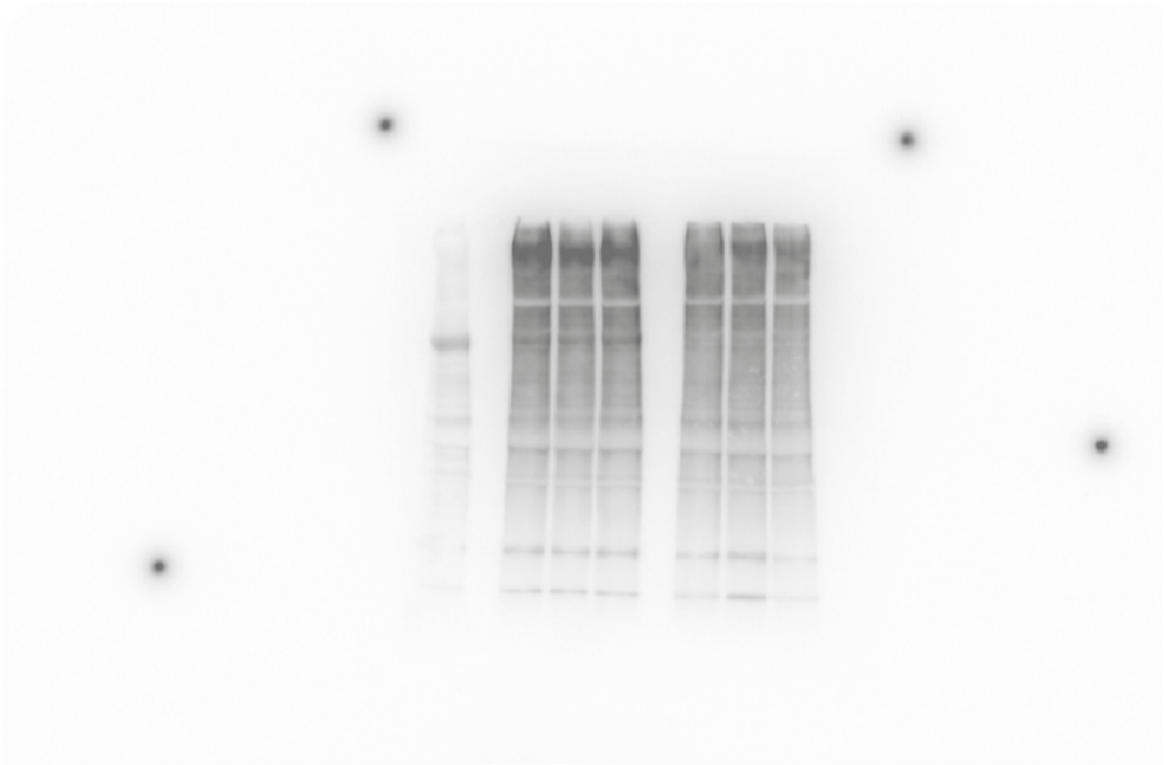

source data Figure S10B

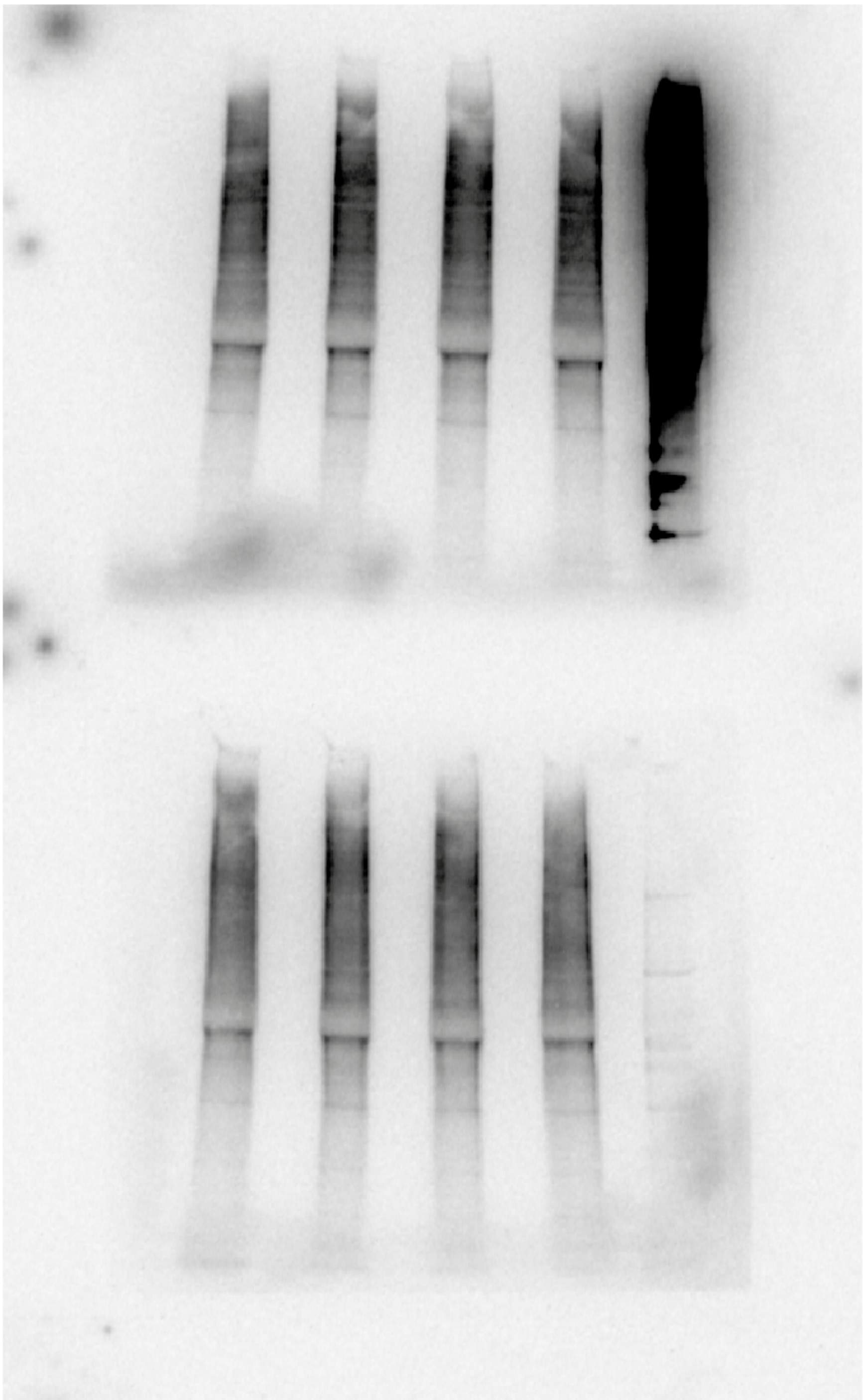

Supplement: Supplementary file 10 — Additional file 10. [file 13059_2021_2298_MOESM10_ESM.pdf]
