# Supplementary material for: SRSF3 and SRSF7 modulate 3′UTR length through suppression or activation of proximal polyadenylation sites and regulation of CFIm levels
Source: Genome Biol. 2021 Mar 11;22:82. doi: 10.1186/s13059-021-02298-y (PMC7948361; doi:10.1186/s13059-021-02298-y)
Supplement: Supplementary file 11 — Additional file 11. [file 13059_2021_2298_MOESM11_ESM.docx]

**Review history**

**First round of review**

**Reviewer 1**

In this manuscript, Schwich et al reported that Srsf3 and Srsf7 regulate alternative polyadenylation (ApA) and dissected the molecular mechanisms. The main findings include: 1) knockdown of Srsf3 and Srsf7 lead to the opposite ApA changes; 2) Srsf3 and Srsf7 preferentially bind to upstream poly(A) sites; 3) Srsf7 binds to Fip1 and CFIm and the phosphorylation status of its RS domain modulates the interaction; 4) Srsf7 levels decrease during P19 cell differentiation; 5) Srsf3 promotes distal PAS usage by maintaining high levels of CFIm; 6) CFIm inhibits proximal PAS through unproductive Fip1 recruitment.

Overall the data and analyses in this manuscript are of high quality and the manuscript was very well written. The results will be of interest to the field. On the other hand, there are several major issues that need to be addressed. Additionally all of the observations in the paper need to be better integrated into a coherent model. My comments are below:

1.     Are the effect of Srsf3 or Srsf7 on ApA direct or indirect? Although Srsf3 knockdown caused more ApA changes, the authors suggest that this effect is through altering CFIm levels, thus making it an indirect effect. As SR proteins are multi-functional proteins involved in splicing, 3' processing, export, and RNA degradation, it is important to demonstrate that Srsf7 or Srsf3 directly modulate poly(A) site selection.
2.     Both Srsf3 and 7 preferentially bind to proximal PAS of the regulated ApA events. Is this due to an enrichment of Srsf3 and 7 consensus binding motifs or are these SR proteins recruited to the proximal sites via interactions with other factors? If their binding motifs are enriched at the proximal sites, they should be mutated/removed to examine the effect on ApA regulation. Additionally the Srsf3 or 7 binding sites should be introduced to heterologous sites and see if they confer sensitivity to Srsf3 or 7.
3.     The mechanistic model is a bit confusing. Srsf7 interacts with both Fip1 and CFIm. As CFIm has been shown to bind to Fip1, are the authors suggesting that Srsf7 competes with CFIm for binding to Fip1 or does it promote CFIm-Fip1 interaction?
4.     The authors argue that Srsf3 promotes dPAS usage by maintaining high levels of CFIm (Fig. 6). If this model is correct, the ApA changes induced by Srsf3 knockdown should be rescued (at least partially) by over-expressing CFIm in Srsf3-depleted cells. This should be tested.

**Reviewer 2**

Schwich et al. reported antagonistical roles between SRSF3 and SRSF7 on 3'UTR length regulation, which has significance for understanding the mechanism of the regulation of 3'UTR length. The different functions of SRSF3 and SRSF7 are mediated by small differences in domain architectures. Authors also illustrated how SRSF3 and SRSF7 engage in the regulation of alternative polyadenylation and their roles in neuronal differentiation. In particular, this work helps to understand how APA sites are chosen based on subtle difference in concentration of poly(A) factors.

However, I am not sure on the point of APA regulation by SRSF3 and SRSF7 is splicing-independent. Although authors proved it by fusion experiments in Fig. 2, SRSF3 regulates splicing of CPSF6 (Fig. 6) and we can find CPSF6 has multiple poly(A) sites in genome browser on ENSEMBL website. However, authors only showed one poly(A) site in Fig. 6C and D (exon 7). Is the APA of CPSF6 also splicing-independent?

Other comments:

1.     Use the full name or point out what kind of protein on the title instead of SRSF3, e.g. SR splicing factor…. Also, it seems it is an over-statement that SRSF3 is strongly regulating APA over SRSF7 because that data is not so strong in terms of RNA binding preferences and the outcome of how many genes are affected by SRSF3 alone.  
2.     Page 2, line 35, "both factors" are which two?
3.     Page 7, line 2, how to remove PCR duplicates? By sequence similarity or unique molecular identifier? Need to address it in method part.
4.     Fig. 1, there are many abbreviations like pPAS, oPAS etc., please annotate them in the legend.
5.     Page 8, line 56, what is "newly mapped" sites? Cryptic poly(A) sites?
6.     Page 9, line 5. Binding of SRSF7 is stronger than SRSF3. However, authors didn't address how they normalized the data. Did they use the same method? In my opinion, normalized to 1X genomic coverage (I think in deepTools has a RPGC option) is better for different IP comparison. Also, statistic test should be applied to test the significance of binding difference between SRSF3 and SRSF7 (also in Fig. 2B, Fig. 5G and Fig. 7F).
7.     Fig. 2B, how did the Z-score show on the figure is not apparent.  The color different is subtle so it's difficult to see.
8.     Page 9, line 14. I cann't understand the lower panel of Fig. S2A and B.  A detailed legend should be provided.
9.     Page 11, line 30. Where do the "binding sites" come from? If they used previously identified binding sites, add a citation.
10.     Page 12, line 49-50, I don't agree with this sentence. Before authors get TetR experiment results, there's no hint showed RS domains are associated with the recruitment of SRSF7.
11.     Page 19, line 23-24, add P values to corresponding figures even though they were not significant.
12.     There are many issues with Fig.8 legend. For example, there is no Lower panel in A. In B, there is too brief annotation of the graph and symbols (what is 5?).
13.     In a number of figures, the "X" was used to mean killing the factors. But it is so thick to cover the words, make it difficult to see the words.

**Authors’ response to reviewers**

Reviewer #1:

In this manuscript, Schwich et al reported that Srsf3 and Srsf7 regulate alternative polyadenylation (ApA) and dissected the molecular mechanisms. The main findings include: 1) knockdown of Srsf3 and Srsf7 lead to the opposite ApA changes; 2) Srsf3 and Srsf7 preferentially bind to upstream poly(A) sites; 3) Srsf7 binds to Fip1 and CFIm and the phosphorylation status of its RS domain modulates the interaction; 4) Srsf7 levels decrease during P19 cell differentiation; 5) Srsf3 promotes distal PAS usage by maintaining high levels of CFIm; 6) CFIm inhibits proximal PAS through unproductive Fip1 recruitment.

Overall the data and analyses in this manuscript are of high quality and the manuscript was very well written. The results will be of interest to the field. On the other hand, there are several major issues that need to be addressed.

Response: We thank the reviewer for the overall positive comments. We now added three more experiments and two more analyses to address the issues raised by the reviewer.


Additionally, all of the observations in the paper need to be better integrated into a coherent model.

Response: We simplified the model and explain it now more comprehensibly in the discussion.


My comments are below:

1. Are the effect of Srsf3 or Srsf7 on ApA direct or indirect? Although Srsf3 knockdown caused more ApA changes, the authors suggest that this effect is through altering CFIm levels, thus making it an indirect effect. As SR proteins are multi-functional proteins involved in splicing, 3' processing, export, and RNA degradation, it is important to demonstrate that Srsf7 or Srsf3 directly modulate poly(A) site selection.

Response: In this manuscript, we describe two different mechanisms how SRSF7 and SRSF3 modulate poly(A) site selection. SRSF7 acts always directly, and SRSF3 acts both directly and indirectly. The conclusion that both proteins can directly regulate poly(A) site selection is based on a number of experimental observations: I) In our iCLIP data, both proteins are enriched upstream of regulated pPASs. II) Our reporter gene experiments suggest that SRSF7 enhances pPAS usage in a concentration and binding-dependent manner. SRSF7 overexpression enhances pPAS usage, while Srsf7 knockdown reduces it. Adding more binding sites for SRSF7 (allSRSF7) enhances pPAS usage, while removing them (allSRSF3) reduces it. III) Our biochemical assays show that SRSF7 directly recruits FIP1. This depends on SRSF7-specific features, namely the Zn knuckle and a 27aa stretch as well as hypophosphorylation of the RS domain. IV) Overexpression of a SRSF7 mimic (SRSF3 containing a Zn knuckle and 27aa stretch) also enhances pPAS usage. V) SRSF3 does not recruit CPA factors. VI) SRSF3 competes with SRSF7 for binding to pPASs and this way inhibits their activation, since increasing SRSF3 binding sites (allSRSF3) reduced pPAS usage while removing them (allSRSF7) enhanced it. We currently do not know whether SRSF3 only prevents pPAS activation, or on top of that actively inhibits their usage by additional mechanisms.

In order to substantiate that both proteins can directly modulate poly(A) site selection, we now performed further reporter gene experiments in which we mutated the binding sites of SRSF3 and SRSF7 in a target 3’UTR. The results are described in more detail below (response to the reviewer’s comment #2) and shown in the new Figure 2G,H.

As pointed out by the reviewer, in addition to the direct effects, we found that SRSF3 also affects pPAS usage indirectly by controlling the levels of CPSF6, which is highly relevant for the understanding of APA regulation. The indirect mode of regulation via CPSF6 is further supported by the new experiments that we performed following the reviewer’s suggestion in comment #4 below. In brief, we transiently overexpressed CPSF6 which allowed to partially rescue the effect of the Srsf3 knockdown for some target genes, supporting that SRSF3 can influence APA indirectly via regulating CPSF6 levels. The new experiments are described in more detail below (response to the reviewer’s comment #4) and shown in the new Figure S8F,G.


2. Both Srsf3 and 7 preferentially bind to proximal PAS of the regulated ApA events. Is this due to an enrichment of Srsf3 and 7 consensus binding motifs or are these SR proteins recruited to the proximal sites via interactions with other factors? If their binding motifs are enriched at the proximal sites, they should be mutated/removed to examine the effect on ApA regulation. Additionally the Srsf3 or 7 binding sites should be introduced to heterologous sites and see if they confer sensitivity to Srsf3 or 7.

Response: We thank the reviewer for this suggestion. We searched for the recognition motifs CNYC (SRSF3) and GAY (SRSF7) in the vicinity of pPASs and dPASs. We found that the recognition motifs of both proteins are enriched upstream of pPASs but not upstream of dPASs. This suggests that both proteins bind directly to pPASs and are not recruited by other factors. SRSF7 motifs are particularly enriched in SRSF3-regulated targets, in line with a competition for binding.

Metaprofiles of the motif enrichment for CNYC (SRSF3) and GAY (SRSF7) are shown in new Figure S2C-H.

We agree with the reviewer that targeted mutations of the binding sites offer essential support for a direct role of both proteins at the regulated pPASs. The original version of the manuscript had included experiments with our LUC-Ddx21 reporter gene harboring the 3’UTR of the target gene Ddx21 (fused to the luciferase gene), in which we removed the binding sites of either protein (now Figure S3E,F). In order to substantiate the findings, we now performed new experiments with analogous mutations in our mCherry-Ddx21 reporter gene. To this end, we converted all SRSF3 recognition motifs upstream of the pPAS to SRSF7 recognition motifs, which significantly enhanced pPAS usage. Similarly, we converted all SRSF7 motifs to SRSF3 motifs and observed the opposite effect, an inhibition of pPAS usage. This antagonistic modulation of pPAS usage was abolished when we changed the strength of the CSE. We generated an additional construct in which we removed all binding sites of SRSF3 and SRSF7, but introduced a UGUA motif, which now favored pPAS usage. Together, these data strongly argue for a direct effect of SRSF7 and SRSF3 binding on the modulation of a subset of pPASs, e.g. those with intermediate CSEs and without UGUA motifs, as seen in Ddx21.

The new reporter experiments are shown in the new Figure 2G,H.

In the future, we hope to be able to design synthetic SRSF3/SRSF7-regulated APA events by heterologous insertions of SRSF3/SRSF7 binding sites in a previously non-regulated reporter gene, as suggested by the reviewer. This will most likely involve a series of reporters to scan for the optimal setup, such as the required number of binding sites and their precise positioning in relation to each other and the pPAS, which we consider beyond the scope of this study.


3. The mechanistic model is a bit confusing. Srsf7 interacts with both Fip1 and CFIm. As CFIm has been shown to bind to Fip1, are the authors suggesting that Srsf7 competes with CFIm for binding to Fip1 or does it promote CFIm-Fip1 interaction?

Response: We agree with the reviewer and are grateful for the interesting ideas. Our experiments indicate that SRSF7 binds directly to FIP1 independently of CFIm. The interaction requires hypophosphorylation of the RS domain of SRSF7. This indicates that SRSF7 is able to recruit FIP1 in its RNA-bound state, e.g. at the pPAS, which usually involves hypophosphorylation of the RS domain (partial dephosphorylation occurs during splicing). We hypothesize that SRSF7 could thereby recruit FIP1 to pPASs that lack a nearby UGUA motif and hence are not bound by CFIm, like in the case of the Ddx21 3’UTR.

The interaction between CFIm and SRSF7 is more puzzling and it seems to be different from the FIP1 interaction. In the case of CFIm, the interaction requires the RNA-binding domain of SRSF7, meaning that it would most likely be blocked from RNA binding, and RS domain hyperphosphorylation. This means that SRSF7 cannot interact with CFIm when it is bound to RNA, and hence cannot recruit CFIm to pPASs. It is important to note that this difference could only be worked out due to our detailed biochemical characterizations and TetR fusion constructs.

Based on these observations, we can evaluate the two options posed by the reviewer how SRSF7 may impact on CFIm and Fip1. It seems that the different molecular context of the two interactions argues against the second option that SRSF7 may promote the CFIm-Fip1 interaction. Following from the direct interaction with Fip1, we hypothesize that SRSF7 can substitute for CFIm to promote pPAS usage in targets like Ddx21, which lack a nearby UGUA motif. We presently do not know whether SRSF7 can outcompete CFIm if both are present. It could be that CFIm will be dominant then but this would have to be tested experimentally.

Given that SRSF7 and CPSF6 share a very similar domain structure, it would also be possible that hyperphosphorylated SRSF7 might form inactive heterotetramers with CPSF6 and CPSF5. Since CFIm primarily acts at the dPAS, this would ultimately also promote pPAS usage. This possibility is, however, highly speculative and would require further investigations.


4. The authors argue that Srsf3 promotes dPAS usage by maintaining high levels of CFIm (Fig. 6). If this model is correct, the ApA changes induced by Srsf3 knockdown should be rescued (at least partially) by over-expressing CFIm in Srsf3-depleted cells. This should be tested.

Response: We thank the reviewer for this excellent suggestion. We have now included experiments in which we transiently overexpressed CPSF6-myc from a plasmid in Srsf3-depleted cells to rescue CPSF6 levels. Indeed, CPSF6 overexpression counteracted the 3’UTR shortening upon Srsf3 knockdown in three out of five tested SRSF3-regulated APA events. These experiments may thus allow us to distinguish direct SRSF3 targets, e.g. Ddx21 and Anp32e, from indirect SRSF3-CPSF6 targets, e.g. Rab11a, Phpln1 and Tnpo3. We note that the effect of CPSF6 overexpression on APA was small and the rescue was incomplete, possibly because ectopic CPSF6 expression did not restore CPSF5 levels in the Srsf3-depleted cells.

The CPSF6 overexpression experiments are shown in the new Figure S8F,G.


 
Reviewer #2:

Schwich et al. reported antagonistical roles between SRSF3 and SRSF7 on 3'UTR length regulation, which has significance for understanding the mechanism of the regulation of 3'UTR length. The different functions of SRSF3 and SRSF7 are mediated by small differences in domain architectures. Authors also illustrated how SRSF3 and SRSF7 engage in the regulation of alternative polyadenylation and their roles in neuronal differentiation. In particular, this work helps to understand how APA sites are chosen based on subtle difference in concentration of poly(A) factors.

Response: We thank the reviewer for the overall positive comments.


However, I am not sure on the point of APA regulation by SRSF3 and SRSF7 is splicing-independent. Although authors proved it by fusion experiments in Fig. 2, SRSF3 regulates splicing of CPSF6 (Fig. 6) and we can find CPSF6 has multiple poly(A) sites in genome browser on ENSEMBL website. However, authors only showed one poly(A) site in Fig. 6C and D (exon 7). Is the APA of CPSF6 also splicing-independent?

Response: We agree with the reviewer that our claim that APA regulation by SRSF3 and SRSF7 is splicing-independent is not entirely correct. We have revised this in the text. We now state that the direct modulation of pPAS usage by SRSF7 and by SRSF3 is splicing-independent. This conclusion is based on our experiments with reporter genes that do not contain introns but show APA modulation by SRSF3 and SRSF7 similar to endogenous transcripts.

However, the indirect effect of SRSF3 on APA is splicing-dependent, as SRSF3 controls the levels of CPSF6 via alternative splicing. Specifically, we show that SRSF3 binds massively to exon 6 of Cpsf6 and enhances its inclusion. When Srsf3 is depleted, exon 6 is skipped and the resulting transcripts contain premature stop codons and are highly unstable. Low levels of functional Cpsf6 transcripts reduce the levels of CPSF6 and CPSF5 proteins, since both proteins stabilize each other, so that the entire CFIm complex is limiting.

Following the reviewer’s suggestion we investigated whether SRSF3 regulates Cpsf6 levels additionally via APA. Please note that this cannot be evaluated from Figure 6C which only shows RNA-Seq data on exons 5 to 7 and does not reach up to the end of the Cpsf6 transcript in exon 10. In order to make it more clearly visible that the transcript continues beyond exon 7, we now added the start of the flanking intron in the figure.

Next, we used our MACE-seq data to assess APA of Cpsf6 transcripts in P19 cells (see Reviewer Figure I below). Indeed, we detected the usage of multiple PASs in the terminal exon, several more than annotated in GENCODE gene annotation (version M18, shown above). Some of these PASs seemed to be mildly modulated by Srsf7 or Srsf3 knockdown, but none of them passed the thresholds of our DaPars analysis. Thus, to verify whether SRSF3 regulates CPSF6 levels via APA and, if so, whether this is splicing-independent would require further investigation.

Reviewer Figure I: Cpsf6 harbors multiple poly(A) sites (PASs) in the terminal exon. Genome browser view shows coverage of MACE-seq reads in exon 8 of the Cpsf6 gene (minus strand) in control conditions (Ctrl, grey) and upon knockdown of Srsf3 (blue) or Srsf7 (orange) in P19 cells. PAS positions inferred from the MACE-seq data are shown below (dPAS, distal PAS. pPAS, proximal PAS. oPAS, other PAS). Annotated transcripts from GENCODE gene annotation (version M18) are shown above for comparison.


Other comments:

1. Use the full name or point out what kind of protein on the title instead of SRSF3, e.g. SR splicing factor…. Also, it seems it is an over-statement that SRSF3 is strongly regulating APA over SRSF7 because that data is not so strong in terms of RNA binding preferences and the outcome of how many genes are affected by SRSF3 alone.

Response: We changed the title accordingly.


2. Page 2, line 35, "both factors" are which two?

Response: We rephrased this part of the abstract.


3. Page 7, line 2, how to remove PCR duplicates? By sequence similarity or unique molecular identifier? Need to address it in method part.

Response: The MACE-seq data included unique molecular identifiers (UMIs) to remove PCR duplicates. The UMIs stem from a MACE-Kit from GeneXPro, with which our MACE-seq libraries were generated (https://genxpro.net/macekit/). We now included this information in the Methods section. The full analysis pipeline is described in Müller et al, 2014, Database (Oxford), doi: http://doi.org/10.1093/database/bau076.


4. Fig. 1, there are many abbreviations like pPAS, oPAS etc., please annotate them in the legend.

Response: We carefully revised Figure 1 and all following figures to ensure that all abbreviations are introduced in the associated legends.


5. Page 8, line 56, what is "newly mapped" sites? Cryptic poly(A) sites?

Response: “Newly mapped” referred to all poly(A) sites that we mapped in our analysis. The term was used to distinguish them from poly(A) sites that were retrieved from databases. We changed it to “our mapped” poly(A) sites.


6. Page 9, line 5. Binding of SRSF7 is stronger than SRSF3. However, authors didn't address how they normalized the data. Did they use the same method? In my opinion, normalized to 1X genomic coverage (I think in deepTools has a RPGC option) is better for different IP comparison. Also, statistic test should be applied to test the significance of binding difference between SRSF3 and SRSF7 (also in Fig. 2B, Fig. 5G and Fig. 7F).

Response: We agree with the reviewer that normalization of the iCLIP data is critical to compare the binding signals of both proteins.

As mentioned by the reviewer, the RPGC method normalizes to 1x genomic coverage, which is defined as: (mapped reads x fragment length) / effective genome size. Since iCLIP has a single-nucleotide resolution, the fragment length in the formula can be ignored, meaning that RPGC would effectively normalize to the number of mapped reads.

From our experience with many iCLIP datasets for different proteins and conditions, we developed a similar but slightly modified approach. In essence, we use crosslinked nucleotides rather than crosslink events (which would be equivalent to mapped reads) and normalize the signals to the number of genomic positions with at least one crosslink event. As the vast majority of genomic positions have very few crosslink events (the background mainly shows 1 crosslink event per position), our procedure closely approaches the number of mapped reads, but reduces the bias from a small set of regions with very high signal. These are often found in highly expressed genes such as some lncRNAs, ribosomal proteins etc. which can otherwise dominate the metaprofiles.

Following the reviewer’s suggestion, we tested the significance of the binding difference using two-proportion z-tests for each position of the analyzed windows. We labelled all positions with a significant binding difference (FDR ≤ 0.01) in Figure 2A and S9C.


7. Fig. 2B, how did the Z-score show on the figure is not apparent. The color different is subtle so it's difficult to see.

Response: The z-score reflects the binding difference between SRSF3-regulated and non-regulated transcripts in a nucleotide-wise manner. We agree with the reviewer that the color gradient was hard to resolve by eye. We now uniformly marked all positions with FDR ≤ 0.01 in Figures 2B, 5G, 7F and S9D.


8. Page 9, line 14. I cann't understand the lower panel of Fig. S2A and B. A detailed legend should be provided.

Response: We extended the figure legend of Figure S2A,B to provide more details on both panels and the described analysis steps.


9. Page 11, line 30. Where do the "binding sites" come from? If they used previously identified binding sites, add a citation.

Response: The reviewer is correct. We have changed “binding sites” to “binding motifs”. The motifs were identified from our iCLIP data in a previous study (Müller-McNicoll, 2016). We now provide more details on the motifs including citations in the previous section when describing the newly included motif search (see also comment #2 of reviewer #1).


10. Page 12, line 49-50, I don't agree with this sentence. Before authors get TetR experiment results, there's no hint showed RS domains are associated with the recruitment of SRSF7.

Response: We agree with the reviewer that this statement appeared to come out of nowhere. We changed the text accordingly.


11. Page 19, line 23-24, add P values to corresponding figures even though they were not significant.

Response: We apologize if this was unclear. The Venn diagram in Figure 5E shows the overlap of significant APA changes upon Srsf7 knockdown and P19 differentiation. This is based on the thresholds of our DaPars analysis, i.e. a difference in distal poly(A) site usage index [|ΔPDUI|] ≥ 0.05 and false discovery rate [FDR] ≤ 0.1. Due to the small overall number of detected SRSF7 targets, the overlap appears rather small. However, when looking at the detected APA changes in differentiation, irrespective of whether they pass the thresholds upon Srsf7 knockdown, we observe a very consistent trend towards longer 3’UTRs as shown in Figure 5F. Hence, in this figure, we used the above thresholds only for the differentiation dataset, but omitted them for the Srsf7 knockdown dataset.

We rephrased the respective sentence in the text and extended the figure legend to make clear which thresholds were applied for which dataset.


12. There are many issues with Fig.8 legend. For example, there is no Lower panel in A. In B, there is too brief annotation of the graph and symbols (what is 5?).

Response: We have corrected this.


13. In a number of figures, the "X" was used to mean killing the factors. But it is so thick to cover the words, make it difficult to see the words.

Response: We adjusted the line width of X in all figures as requested.

**Second round of review**

**Reviewer 1**

The authors have addressed my concerns satisfactorily.

**Reviewer 2**

The authors addressed my concerns.
